# Evolution of the vertebrate goose-type lysozyme gene family

**DOI:** 10.1186/s12862-014-0188-x

**Published:** 2014-08-29

**Authors:** David M Irwin

**Affiliations:** Department of Laboratory Medicine and Pathobiology, Faculty of Medicine, University of Toronto, 1 King’s College Circle, Toronto, Ontario M5S 1A8 Canada; Banting and Best Diabetes Centre, University of Toronto, Toronto, Ontario Canada

**Keywords:** Lysozyme g, Vertebrates, Gene duplication, Genome evolution, Phylogeny

## Abstract

**Background:**

Lysozyme *g* is an antibacterial enzyme that was first found in the eggs of some birds, but recently has been found in additional species, including non-vertebrates. Some previously characterized lysozyme *g* sequences are suggested to have altered secretion potential and enzymatic activity, however the distribution of these altered sequences is unknown. Duplicated copies of the lysozyme *g* gene exist in some species; however, the origins of the duplicates and their roles in altered function are unclear.

**Results:**

We identified 234 lysozyme *g* sequences from 118 vertebrate species, including 181 sequences that are full or near full length representing all vertebrate classes except cartilaginous fish. Phylogenetic analysis shows that most lysozyme *g* gene duplicates are recent or lineage specific events, however three amplification events are more ancient, those in an early amniote, an early mammal, and an early teleost. The older gene duplications are associated with changes in function, including changes in secretion potential and muramidase antibacterial enzymatic activity.

**Conclusions:**

Lysozyme *g* is an essential muramidase enzyme that is widespread in vertebrates. Duplication of the lysozyme *g* gene, and the retention of non-secreted isozymes that have lost enzymatic activity indicate that lysozyme *g* has an activity other than the muramidase activity associated with being an antibacterial enzyme.

**Electronic supplementary material:**

The online version of this article (doi:10.1186/s12862-014-0188-x) contains supplementary material, which is available to authorized users.

## Background

Lysozyme is an antibacterial enzyme that has been a model system for understanding enzymology [1,2], protein structure [3–5], and gene regulation [6,7]. The best-characterized lysozyme is lysozyme *c* (chicken-type or conventional lysozyme), which is typically secreted into body fluids (e.g., blood, sweat, tears, and milk) of mammals and is found in the egg whites of many birds [8–11]. Protein and gene sequences for lysozyme *c* have been characterized from a diverse array of vertebrate and non-vertebrate species [8–11]. It has long been known that lysozyme *c* is a member of a gene family, with the two other well-characterized members being lactalbumin and calcium-binding lysozyme [8–10]. Genome sequence data has led to the realization that the lysozyme *c* gene family is much larger than expected, with 10 genes identified in the human genome and similar numbers in other mammals [12]. A number of additional types of lysozymes that show limited or no significant similarity to lysozyme *c* have been identified [13,14]. The antibacterial lysozyme isolated from goose eggs, lysozyme *g*, was found to be larger than and have no significant sequence similarity to lysozyme *c* [9,11,13,14]. Lysozyme *g* is also found in other vertebrates and a few non-vertebrate species [9,11]. Additional types of lysozyme have been found in invertebrates (lysozyme *i*) [11,15], plants [16], bacteria [17], and bacteriophage [18]. These different forms of lysozyme share limited or no sequence similarity; however, protein crystal structures suggest they share similar structures raising the possibility that they have a common ancestor [14,19,20].

Lysozyme *g* was initially identified from the egg white of the goose and some other bird species [21,22]. Low levels of lysozyme *g* were also detected in a few other tissues of the goose [23]. The first lysozyme *g* gene to be cloned was from the chicken, a species that does not express this enzyme in eggs [24]. Chicken lysozyme *g* was found to be specifically expressed in cells of the bone marrow and in the lung [24]. In contrast to birds, lysozyme *g* appears to have a broader expression pattern in fish [25–28]. Fish lysozyme *g* retains antibacterial properties, and its expression is often induced in response to bacterial infection [26–28]. Many fish lysozyme *g* sequences do not predict signal peptides, suggesting they may have an intracellular function [26,28,29], however some do contain signal peptides due to the presence of an alternative 5′ spice acceptor in the second coding exon [30]. In mammals, a pair of lysozyme *g* genes has been identified, however little is known about their function [29].

Multiple lysozyme *g* genes have been identified in several species such as mammals [29], chicken [31], zebrafish [29], and urochordates [32]. A phylogenetic analyses conducted with the limited number of sequences available about 10 years ago indicated that the duplicated lysozyme *g* genes in mammals, zebrafish and urochordates were products of independent gene duplications [29,32]. Since recent analyses of vertebrate genomes have indicated that the lysozyme *c* gene family is much larger than previously appreciated [12], and a large number of vertebrate genome sequences are now available, we hypothesized that the vertebrate lysozyme *g* gene family may show a similar increase in size. Here we show that there is indeed a family of lysozyme *g* genes, where an ancestral amniote (i.e., ancestor of birds, mammals, and reptiles) had three distinct lysozyme *g* genes, and that the previously characterized bird egg white lysozyme *g* genes are not orthologous to the mammalian lysozyme *g* genes.

## Results

### Number of lysozyme *g* genes in vertebrate genomes

To determine the number of lysozyme *g* genes in the genomes of diverse vertebrate species, we used *BLAST* [33] to search the *Ensembl*, *Pre!Ensembl*, and *NCBI* databases [34–36]. Genes were given names (see Additional files 1 and 2: Tables S1 and S2) based on their orthology-paralogy relationships derived from phylogenetic analysis, sequence similarity, and genomic location as discussed below. The numbers of species searched and sequences found are listed in Table 1. As expected, only two genomic sequences that predict sequences similar to lysozyme *g* were found in the human genome, the sequences that encode the known lysozyme *g*1 (*LYG1*) and lysozyme *g*2 (*LYG2*) genes [30] (here now called *LYGA1* and *LYGA2* to better reflect the diversity of lysozyme *g* genes – see below) (Additional files 1 and 2: Tables S1 and S2). Previous analyses had suggested that a duplication of the lysozyme *g* gene had occurred on the mammalian lineage, leading to the duplicated lysozyme *g* genes in the human, mouse, and rat genomes [29]. Here, our screen of a large number of mammalian genomes in the *Ensembl*, *Pre!Ensembl*, and *NCBI* databases [34–36] found that most species had two genomic sequences similar to lysozyme *g* (Table 1 and Additional files 1 and 2: Tables S1 and S2) consistent with this conclusion. The number of lysozyme *g* genes in a few mammalian species differed from 2 (Table 1 and Additional files 1 and 2: Tables S1 and S2). Several species were suggested to have only one lysozyme *g* gene (e.g., alpaca, camel, and killer whale; Additional files 1 and 2: Tables S1 and S2), however, this may simply be a consequence of incomplete genome sequences or assembly (i.e., a second gene exists in a gap in these genome assemblies). Several species (e.g., cow, sheep, pig, and dolphin; Additional file 1: Table S1) had two genomic sequences that were similar to lysozyme *g*, but only one of them was annotated as an intact lysozyme *g* gene, with the second sequence failing to predict a complete open reading frame (therefore not annotated as a gene). Some of the un-annotated genes may reflect partial gene sequence, due to gaps in the genome, but several (e.g., cow, sheep, and pig) appear to be genuine pseudogenes that have accumulated mutations that prevent translation (see Additional file 3: Figure S1). A few mammalian species had more than two lysozyme *g* genes (Table 1 and Additional file 1: Table S1). The little brown bat (microbat) has three genomic sequences similar to lysozyme *g*, however one of these only predicts part of a coding sequence (Additional file 1: Table S1). Whether this sequence represents an additional gene, or is an assembly artifact is unclear. The hedgehog had three genomic scaffolds with similarity to lysozyme *g*, however it is possible that two of these (scaffolds 182805 and 371836) are fragments of a single gene as they are non-overlapping (Additional file 1: Table S1). The rat was found to have 4 segments of its genome that had similarity to lysozyme *g* (Additional file 1: Table S1), however only two lysozyme *g* coding sequences (CDS) are in the *NCBI* database (Additional file 2: Table S2). Examination of the rat genomic sequences indicates that the sequence contains a potential segmental duplication (i.e., long genomic sequences that are nearly identical in sequence [37]) containing duplicated lysozyme *g* genes, thus there are two nearly identical copies of a pair of lysozyme *g* genes in the rat genome (see Additional file 4: Figure S2).

**Table 1:** **Numbers of lysozyme** ***g*** **genes found in diverse vertebrates**

	**Species** ^**a**^	**Genes/CDS** ^**b**^	**Range**	**Intact** ^**c**^
Mammals	63	125	1 - 4	100
Birds	13	22	1 - 3	21
Reptiles	6	22	1 - 6	17
Amphibians	3	4	1 - 2	3
Lobe-finned fish	1	2	2	2
Bony fish	30	57	0 - 11	37
Cartilaginous fish	1	0	0	0
Jawless fish	1	2	2	1
Total	118	234	0 - 11	181

^a^Number of species with identified genes or searched (if zero genes found).

^b^Number of unique genes or coding sequences found.

^c^Number of complete or near-complete open reading frames.

A single lysozyme protein had previously been identified in many birds [9,11,29], although a second lysozyme *g* gene had been identified in the chicken [31]. Unexpectedly, we found three genomic sequences encoding sequences similar to lysozyme *g* in the chicken genome, as well as several other bird species (e.g., turkey, duck, and pigeon) (Table 1 and Additional files 1 and 2: Tables S1 and S2). Genomic sequences similar to lysozyme *g* in some bird species (e.g., duck) failed to predict an intact coding sequence, thus are potential pseudogenes (Additional file 1: Table S1). Reptiles have a variable number of genes, with five intact lysozyme *g* genes identified in the genomes of the Chinese soft-shelled turtle and the Chinese alligator (which also contains an additional lysozyme *g* pseudogene; Additional file 2: Table S2) and as few as one in the anole lizard and Burmese python (Table 1 and Additional files 1 and 2: Tables S1 and S2). In the anole lizard the single copy lysozyme *g* gene is separated into 5′ fragment and 3′ fragments (Addition file 1: Table S1), but an intact coding sequence (supported by EST sequences such as accession number FG795243.1) spanning these segments was found in the NCBI database (Additional file 2: Table S2). In the Chinese alligator, a predicted CDS was found in the NCBI database that was derived from the genome data that predicted an open reading frame containing duplicated lysozyme *g*-like sequences (Additional file 2: Table S2). An examination of the genomic sequence suggests that instead, this open reading frame is an artifact caused by the merger of two distinct lysozyme *g* genes (*LygB2* and *LygC*) that are arranged in tandem (Additional file 5: Figure S3). In the subsequent analyses we used our two predicted lysozyme *g* coding sequences instead of the predicted merged lysozyme *g* sequence.

Lysozyme *g* sequences were found in only three amphibian species, a group of species with limited genomic representation (Table 1 and Additional files 1 and 2: Tables S1 and S2). The *Xenopus tropicalis* genome contains two lysozyme *g* sequences, one of which predicts a complete coding sequence (Additional file 1: Table S1), while a single lysozyme *g* sequence was found in the other two amphibian species (Additional file 2: Table S2). Similarly, a pair of lysozyme *g* genes was found in the coelacanth genome (Table 1 and Additional file 1: Table S1), a lobe-fined fish that is more closely related to tetrapods than to other fish [38,39]. Like birds, the number of lysozyme *g* genes found in bony fish is variable (Table 1 and Additional files 1 and 2: Tables S1 and S2). While a single lysozyme *g* gene was found in many species, including several species with near complete genomes, some had several lysozyme *g* genes (e.g., zebrafish and medaka) while the cod may have as many as 11 (Additional files 1 and 2: Tables S1 and S2). Some of the cod lysozyme *g* genes are distributed on small genomic contigs, thus it is difficult to determine how many genes these fragments represent, and whether they encode intact protein coding sequences. We failed to identify a lysozyme *g* gene in the genomes of two bony fish with near complete genome sequences, the gar and the talipia, which may be due to gene loss or gaps in their genome assemblies (Additional file 1: Table S1). There are few genomic resources for cartilaginous fish. Recently a more complete version of the genome of a cartilaginous fish, the elephant shark, has become available [40]. Searches of the elephant shark genome failed to identify a lysozyme *g*-like gene (Table 1 and Additional file 1: Table S1), although it is possible it may reside in a gap that remains in this genome, or may exist in the genomes of other species of cartilaginous fish. The lamprey, a jawless fish, has two lysozyme *g* genes (one nearly complete), each on small genomic contigs (Table 1 and Additional file 1: Table S1).

### Organization of the lysozyme *g* gene cluster in vertebrate genomes

**Figure 1:**
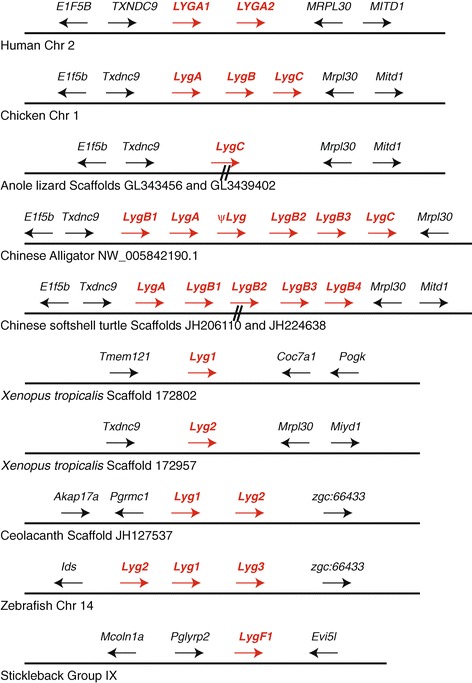
**Genomic organization of genes near lysozyme** ***g*** **genes of representative vertebrate species**. The relative organization and orientation of genes near lysozyme *g* genes in representative diverse vertebrate species. Species and chromosomes (or scaffolds or sequence accessions) are from *Ensembl* [34,35] or *NCBI* [36] (see Additional files 1 and 2: Tables S1 and S2). Lysozyme *g* genes are labeled in red. The Anole lizard and Chinese soft-shelled turtle genomic neighborhoods are composed of two scaffolds that are likely adjacent. In *Xenopus tropicalis*, the lysozyme *g* genes are on two different, likely unlinked, scaffolds. See Additional files 1 and 2: Tables S1 and S2, for details on genomic locations. Gene sizes and distances between genes are not to scale. Arrowheads indicate direction of transcription. Gene symbols are: *Lyg*, lysozyme *g*; *E1f5b*, Eukaryotic translation initiation factor 5B; *Txndc9*, Thioredoxin domain containing 9; *Mrpl30*, Mitochondrial ribosomal protein L30; *Mitd1*, Microtubule interacting and transport, domain containing 1; *Tmem121*, Transmembrane protein 121; *Cox7a1*, Cytochrome c oxidase subunit VIIa polypeptide 1; *Pogk*, Pogo transposable element with KRAB domain; *Akap17a*, A kinase (PRKA) anchor protein 17A; *Pgrmc1*, Progesterone receptor membrane component 1; *zgc:66433*, predicted zebrafish gene; *Ids*, Iduronate 2-sulfatase; *Mcoln1a*, Mucolipin 1a; *Pglyrp2*, Peptidoglycan recognition protein 2; *Evi5l*, Ecotropic viral integration site 5-like.

The multiple lysozyme *g* sequences frequently found in vertebrate genomes tend to co-localize to single locations in these genomes (Additional file 1: Table S1). Previous work [29] had shown that the human, mouse, and rat lysozyme *g* genes are arranged in tandem as shown for the human genes in Figure 1. In the human genome, the lysozyme *g* genes (*LYGA1* and *LYGA2*) are separated by about 30 kb, orientated in the same transcriptional orientation, and flanked by the *TCND9* and *MRPL30* genes (Figure 1 and Additional file 6: Figure S4). A similar organization of the pair of lysozyme *g* genes, including flanking genes, was found in the genomes of most mammals (including mammals where the *LygA1* gene is a pseudogene), with the distance between the genes varying to a small extent (Additional files 1 and 6: Table S1 and Figure S4). A few exceptions to this organization were found. A few species (e.g., kangaroo rat, hedgehog, lesser hedgehog tenrec, hyrax, and wallaby) had lysozyme *g* genes on different genomic sequence, however all of these were short and likely represent unassembled genomic sequence and not a reorganized sequence. As mentioned above, only a single lysozyme *g* gene was identified in a few mammalian (e.g., alpaca, sloth, and platypus), however it is possible that a second gene may exist in a gap in these genome assemblies. In addition, a few species had more than two lysozyme *g* genes (e.g., little brown bat and rat). In the little brown bat, two of the lysozyme *g* genes are co-localized, while the third genomic sequence that encodes part of a gene was on a different genomic sequence (Scaffold AAPE02064623) (Additional files 1 and 6: Table S1 and Figure S4). Since all other bats have only two lysozyme *g* genes (Additional files 1 and 2: Tables S1 and S2) we suspect that this additional sequence is an assembly artifact. The rat has four lysozyme *g* genes (see above and Additional file 1: Table S1). The lysozyme *g* genes though, are co-localized with a *lygA1* gene adjacent to a *lygA2* gene, with this pair then duplicated (Additional file 6: Figure S4). The consistency of the organization of the lysozyme *g* genes, including flanking genes, in placental and marsupial mammals suggests this order originated before the divergence of these two groups of mammals.

A similar clustering of lysozyme *g* genes was found in the several species of birds (i.e., chicken, turkey, duck, and flycatcher, see Chicken in Figure 1) that have multiple lysozyme *g* sequences in their genomes (Additional file 1 and 5: Table S1 and Figure S3). *Txdn9* and *Mrpl30* flank the lysozyme *g* genes of birds, like in mammals (Figure 1 and Additional file 7: Figure S5). Similar genomic neighborhoods are also found in reptiles (Figure 1 and Additional file 7: Figure S5). While the anole lizard and Chinese soft-shelled turtle genomes are incomplete, and the gene neighborhoods are split between two genomic sequence scaffolds, contiguous organizations were found for the Burmese python and the painted turtle, species that are closely related to these two species (Additional file 7: Figure S5). This suggests that the conserved genomic neighborhood has existed since the amniote common ancestor of mammals, birds, and reptiles, and since each of these groups contain species that have multiple lysozyme *g* genes in this location (see Figure 1 and Additional files 1, 2, and 7: Tables S1 and S2 and Figure S5), and raises the possibility that the common ancestor had several lysozyme *g* genes.

The genome of the amphibian *Xenopus tropicalis* contains two lysozyme *g* genes that, unlike other tetrapods, are not clustered (Figure 1, Additional file 1: Table S1). The genes neighboring the *Xenopus tropicalis Lyg2* gene are similar to those flanking lysozyme *g* genes in other tetrapods, indicating that it is located in a conserved genomic neighborhood (Figure 1). The genes that flank the second *Xenopus tropicalis* lysozyme *g* gene, *Lyg1*, show no similarity to genes near lysozyme *g* genes in any other species (Figure 1). Within fish, genes near the lysozyme *g* genes do not show any similarity with those flanking the lysozyme *g* genes in tetrapods (Figure 1 and Additional file 8: Figure S6). In the lobe-fined fish, coelacanth, a pair of lysozyme *g* genes are found, which are clustered (Figure 1 and Additional file 1: Table S1). In some fish, two or more lysozyme *g* genes were identified (e.g., zebrafish, cavefish, medaka, takifugu, tetraodon, and cod; Additional files 1 and 8: Table S1 and Figure S6). Within fish, two general patterns of gene neighborhoods were observed. The most common neighborhood is that illustrated by the zebrafish in Figure 1. In zebrafish and cavefish multiple copies of the lysozyme *g* gene are found within this genomic neighborhood, while most other species only had one lysozyme *g* gene (Figure 1 and Additional file 8: Figure S6). In medaka, tetraodon, and takifugu, their lysozyme *g* genes are not clustered into a single location (as seen in zebrafish and cavefish), but rather have two genomic neighborhoods each containing one or more lysozyme *g* genes (Figure 1 and Additional file 8: Figure S6). Some similarity in the genomic neighborhoods flanking the lysozyme *g* genes in the coelacanth and some fish species (e.g., zebrafish) is seen, with a zgc:66433 gene being 3′ to the lysozyme *g* genes in both groups (Figure 1 and Additional file 8: Figure S6). The genomic neighborhood around the second type (see “Phylogeny of Vertebrate Lysozyme *g* Genes” section below) of fish lysozyme *g* gene, (illustrated by the medaka *LygF2a* and *LygF2b* and the tetraodon *LygF2* genes, Additional file 8: Figure S6) shares no similarity with other lysozyme *g* genes. The lamprey lysozyme *g* genes were on short genomic sequences (Additional file 1: Table S1) that did not contain any genes with similarity with genes near lysozyme *g* genes in any other species, suggesting that the genomic neighborhoods is not conserved across all vertebrates (Additional file 8: Figure S6).

### Expression of lysozyme *g* genes

The expression patterns of lysozyme *g* genes in diverse vertebrates were estimated from EST (expressed sequence tag) data collected from the *NCBI* UniGene database [41]. As previously reported [29], both mammalian lysozyme *g* genes are expressed, with *LygA1* expressed in diverse tissues and *LygA2* found in the skin (Additional file 9: Table S3). In the chicken all three lysozyme *g* genes have EST evidence supporting expression, suggesting they are functional, with expression in multiple and overlapping tissues (Additional file 9: Table S3), including tissues identified for two of these genes in previous analyses [24,31]. The anole lizard, a species with limited EST data, has two ESTs encoding lysozyme *g* that suggest expression in the testis (Additional file 9: Table S3), but more importunely these ESTs provide evidence that the two genomic scaffolds represent a single lysozyme *g* gene (Additional file 1: Table S1). Only one of the two *Xenopus tropicalis* lysozyme *g* genes (*Lyg2*) had ESTs supporting expression (Additional file 9: Table S3), with expression in diverse tissue, although the lack of ESTs for the second gene may simply reflect more restricted expression that was not sampled by the EST database. Restricted expression may explain why one of the two lamprey lysozyme *g* genes did not have EST data (Additional file 9: Table S3). Zebrafish, medaka, and catfish lysozyme *g* genes have EST clones that support the broad expression pattern (Additional file 9: Table S3) seen in previous studies [25–28].

### Phylogeny of vertebrate lysozyme *g* genes

**Figure 2:**
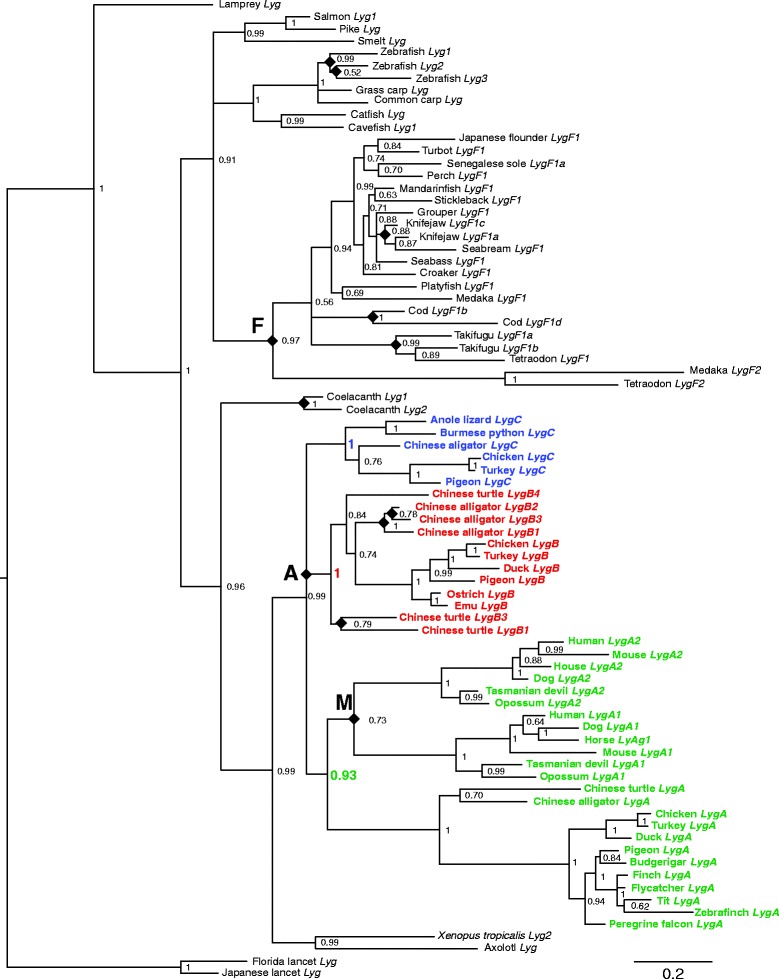
**Phylogeny of vertebrate lysozyme** ***g*** **sequences.** Phylogeny of lysozyme *g* sequences from diverse vertebrate species generated by Bayesian methods. A similar phylogeny was generated by maximum likelihood methods (see Additional file 11: Figure S8). Phylogeny was rooted with sequences from lancets. Numbers at the nodes indicate posterior probabilities. Branch lengths are proportional to the inferred amount of change, with the scale bar at the bottom right. Diamonds indicate gene duplication events. A refers to the duplication in the ancestor of mammals, birds and reptiles (amniotes), M to the duplication on the mammalian lineage, and F the duplication in an early teleost fish lineage. Orthologs in amniotes of the chicken *LygA* gene are labeled in green, chicken *LygB* in red, and chicken *LygC* in blue.

Multiple lysozyme *g* genes exist in diverse vertebrate lineages (Figure 1, Table 1 and Additional files 1 and 2: Tables S1 and S2) raising the possibility that they have an ancient origin. Alternatively, the multiple sequences in different lineages may have independent parallel origins, as was suggested when fewer sequences were available [29]. To resolve this question, and establish names for the genes that reflect their orthology-paralogy relationships, we established a phylogeny of vertebrate lysozyme *g* gene sequences. A total of 181 full and near full-length (those missing only a small portion of their N-terminal sequences, see Table 1 and Additional file 10: Figure S7) coding sequences for lysozyme *g* sequences were identified in vertebrates, with 100 from mammals, 21 from birds, 17 from reptiles, 3 from amphibians, 2 from lobe-finned fish, 37 from bony fish, and 1 from a jawless fish (Table 1). The lysozyme *g* coding sequences were aligned with Mafft [42] and trimmed to remove unreliably aligned codons using Guidance [43]. Shown in Figure 2 is a phylogeny of the diverse vertebrate lysozyme *g* gene sequences established using Bayesian methods [44], with a very similar tree identified using maximum likelihood methods [45] (Additional file 11: Figure S8), or the neighbor-joining distance method (results not shown), using sequences from two species of lancets from the sister subphylum Cephalochrodata of vertebrates to root the tree [46]. Similar trees were obtained by Bayesian, maximum likelihood, and neighbor-joining methods if only the lamprey lysozyme *g* sequence, instead of the lancet sequences, or if other non-vertebrate sequences were used as the outgroup (results not shown).

The two different types of mammalian lysozyme *g* sequences, *LygA1* and *LygA2*, are more closely related to each other than they are to lysozyme *g* sequences of any other vertebrate species (Figure 2 and Additional files 11 and 12: Figures S8 and S9), suggesting that they are derived form a single ancestral gene that duplicated on the mammalian lineage (Duplication labeled as M in Figure 2 and Additional files 11 and 12: Figures S8 and S9). All mammalian *LygA1* genes are orthologous to each other, as are *LygA2* to each other, with these two classes of genes being paralogous. Despite the monophyly of the mammalian lysozyme *g* sequences, the divergence of *LygA1* and *LygA2* occurred prior to the divergence of marsupial and placental mammals (Figure 2 and Additional files 11 and 12: Figures S8 and S9). The partial platypus lysozyme *g* sequence (Additional file 1; Table S1) showed greater similarity to other mammalian *LygA2* sequences, and tended to group with them in phylogenetic trees of the partial sequences (results not shown) suggesting that the duplication that generated these two mammalian lysozyme *g* genes occurred prior to the monotreme – placental mammal divergence.

Like mammals, lysozyme *g* sequences from bony fish (Class Osteichthyes) are monophyletic (Figure 2 and Additional files 11 and 13: Figures S8 and S10). Many of the available fish lysozyme *g* sequences were obtained from cDNA clones (Additional file 2: Table S1), rather genomic sequences (Additional file 1: Table S1) thus for many species the true numbers of genes in these genomes is unknown. Among those species with available near complete genome sequences, two genes were found in tetraodon and cavefish, three in zebrafish, takifugu, and medaka and as many as 11 in the cod (Additional file 1: Table S1). Some lysozyme *g* genes, such as those of zebrafish (*Lyg1*, *Lyg2*, and *Lyg3*) and cavefish (*Lyg1* and *Lyg2*), as well as two (*LygF2a* and *LygF2b*) of the three medaka genes, are found arranged in tandem in their genomes (Figure 1 and Additional files 1 and 8: Table S1 and Figure S6), and thus have an organization similar to that seen for the mammalian genes. The phylogenetic analysis indicates that the lysozyme *g* genes in tandem in the zebrafish (*Lyg1*, *Lyg2*, and *Lyg3*) and medaka (*LygF2a* and *LygF2b*) genomes are products of recent independent gene duplications (Additional file 13: Figure S10). The incomplete cavefish *Lyg2* sequence is most similar to the cavefish *Lyg1* sequence, suggesting another independent parallel gene duplication (results not shown). Many of the cod lysozyme *g* sequences are clustered in the genome (Additional file 1: Table S1), although the complete organization of these genes is uncertain as they reside on several short incomplete genomic contigs, but the two complete lysozyme *g* sequences (cod *LygF1b* and *LygF1d*) are most closely related to each other (Additional file 13: Figure S10), suggesting that they also are products of independent gene duplications. Both medaka (*LygF1* compared to *LygF2a* and *LygF2b*) and tetraodon (*LygF1* compared to *LygF2*) have lysozyme *g* genes that map to distinct locations in the genome (Additional file 1: Table S1), with both residing in distinct genomic neighborhoods (Figure 1 and Additional file 8: Figure S6). Phylogenetic analysis (Additional file 13: Figure S10) suggests that these distinct lysozyme *g* genes in medaka and tetraodon are distantly related to the paralogous copies of this gene in their genomes (with their duplication labeled as F in Figure 2 and Additional files 11 and 13: Figures S8 and S10). These observations suggest that there were both ancient (medaka and tetraodon) and recent (zebrafish, cavefish, medaka, and cod) duplications of the lysozyme *g* gene in fish. The ancient fish-specific gene duplication is incorporated into our proposed names for the lysozyme *g* genes, with the *LygF1* genes being the paralog present in many species and the *LygF2* being found in only a few (see Figure 1 and Additional files 1, 2, 11, and 13: Tables S1 and S2 and Figures S8 and S10). Lysozyme *g* genes in fish species that diverged before the ancient fish-specific gene duplication do not have F in their name. Lineage specific duplicates are distinguished by numbers (in species without the fish-specific duplication) or by letters (with fish-specific duplication); however, the numbers or letters do not infer orthology relationships (see Figure 1 and Additional files 1, 2, 11, and 13: Tables S1 and S2 and Figures S8 and S10).

In contrast to mammals and fish, lysozyme *g* genes from reptiles and birds are found in three different phylogenetic groups (Figure 2 and Additional files 11 and 14: Figures S8 and S14). In each of the three phylogenetic groups, some bird lysozyme *g* gene were more closely related to sequences from reptiles than to other lysozyme *g* genes in their own genome, (Figure 2 and Additional files 11 and 14: Figures S8 and S14). Chicken, turkey, pigeon, and Chinese alligator lysozyme *g* genes were found in all three groups (Figure 2 and Additional files 12 and 15: Figures S9 and S15), suggesting that the triplication of the lysozyme *g* gene occurred prior to the earliest divergence of birds and reptiles in an early amniote ancestor (labeled A in Figure 1 and Additional files 11 and 14: Figures S8 and S11). Consistent with this hypothesis, the mammalian lysozyme *g* genes were most closely related to one of the three phylogenetic groups of amniotic lysozyme *g* genes (Figure 2 and Additional files 11 and 14: Figures S8 and S11). The early amniote duplications yielded three paralogous lysozyme *g* genes that we have names *LygA*, *LygB*, and *LygC*, with any subsequence lineage-specific duplication distinguished by numbers (see Figure 2 and Additional files 1, 2, 11, and 14: Tables S1 and S2 and Figures S8 and S11). The numbers in the names of only the mammalian genes (i.e., *LygA1* and *LygA2*) infer an orthology relationship (see above).

## Discussion

### Structure of lysozyme *g* genes

The structure of lysozyme *g* genes has been conserved throughout vertebrate evolution. Previously characterized bird, fish, and mammalian lysozyme *g* genes contain 5 coding exons, and as many as two additional 5′ untranslated exons [11,24,25,29–31]. We did not attempt to identify untranslated exons in the lysozyme *g* genes, as these sequences typically evolve faster than coding sequence, thus are harder to detect using sequence conservation. In contrast to vertebrates, variation in the structure of the lysozyme *g* gene is seen in non-vertebrate species, with the gene often having fewer exons [32]. All of the newly identified lysozyme *g* genes found in genomic sequences (see Additional file 1: Table S1), including those from reptiles, amphibians, and jawless fish, which had not previously been sampled, are distributed over 5 coding exons, and conserve intron locations and phases, however the sizes of the introns vary greatly (results not shown). These results suggest that a common gene structure has been retained for lysozyme *g* genes since very early in vertebrate evolution.

Initially characterized lysozyme *g* sequences from fish were found to lack a signal peptide, with their genes having shorter coding exon 2 lengths [25–28]. Characterization of the lysozyme *g* gene in the salmon, however, identified alternative splicing of coding exon 2, with a splice variant that yields an exon of similar length to coding exon 2 of the chicken lysozyme *g* gene and predicted a signal peptide [30]. Analysis of genomic sequences of other fish lysozyme *g* genes available at that time suggested that some of these might also encode a similar isoform that could be secreted [30]. Our alignments of genomic sequences of lysozyme *g* genes from diverse fish indicates that the alternatively spliced part of coding exon 2 of the salmon lysozyme *g* gene is not well conserved, with many genomic sequences (e.g., cod *LygF1b* and *LygF1d*, takifugu *LygF1a* and *LygF1b*, and zebrafish *Lyg1* and *Lyg3*) containing inframe stop codons in this region, however, a few genes (e.g., zebrafish *Lyg2*, cavefish *Lyg1*, and medaka *LygF1*) retained an open reading frame, potential splice acceptor sequences and predict potential signal peptides (results not shown). This result suggests that alternative splicing of lysozyme *g* genes is not found in all fish, and that many of the lysozyme *g* sequences do not encode a signal peptide (see Additional file 15: Table S4).

### Duplications of the lysozyme *g* gene

Duplicated lysozyme *g* genes had previously been identified in several vertebrate species, including mammals, zebrafish, and chicken [29,31]. Here we have shown that duplicated lysozyme *g* genes are widespread in vertebrates, with duplicates existing in species in all classes of vertebrates examined except the cartilaginous fish (Additional files 1 and 2: Tables S1 and S2, no sequence similar to lysozyme *g* was identified in the only cartilaginous fish, the elephant shark, which has an available genome sequence). The existence of multiple lysozyme *g* genes in diverse vertebrate species can be explained by either ancient gene duplications generating multiple genes in the common ancestor of vertebrates or parallel recent duplications of lysozyme *g* genes in diverse lineages. An earlier analysis, based on a very small number of lysozyme *g* sequences, concluded that there were independent duplication on the zebrafish and mammalian lineages [29]. The arrangement of lysozyme *g* genes in tandem arrays in most species (Figure 1 and Additional files 4, 5, 6, 7 and 8: Figures S2, S3, S4, S5 and S6) is consistent with recent duplications, however does not exclude the possibility of more ancient duplications. Our phylogenetic analysis of the lysozyme *g* sequences (Figure 2 and Additional files 11, 12, 13 and 14: Figures S8, S9, S10 and 11) demonstrate that there were multiple relatively recent (or lineage specific) parallel duplications of the lysozyme *g* gene, such as those on the lineages leading to the rat (*LygA1a*/*LygA1b* and *LygA2a*/*LygA2b*), alligators (*LygB1*-*LygB3*), turtles (*LygB1*-*LygB4*), coelacanth (*Lyg1*/*Lyg2*), cod (*LygF1b*/*LygF1d*), zebrafish (*Lyg1*-*Lyg3*), and takifugu (*LygF1a*/*LygF1b*) (Figure 2 and Additional files 4 and 11, 12, 13 and 14: Figures S2 and S8, S9, S10 and S11). The reasons for these lineage-specific lysozyme *g* gene expansions are unknown, but potentially could allow specialization in expression or function. However, recent gene duplications cannot explain all of the multiple genes, as at least three relatively ancient amplification events also occurred, those on an ancestral amniote (ancestor of mammals, birds and reptiles), early mammalian, and an early teleost fish lineage (labeled as A, M, and F, respectively in Figure 2 and Additional file 11: Figure S8). The amplification on the early amniote lineage generated three genes, the *LygA*, *LygB*, and *LygC* genes, while those on the early mammalian and teleost lineages yielded 2 genes, the *LygA1* and *LygA2*, and the *LygF1* and *LygF2* genes, respectively.

A pair of duplications (labeled A in Figure 2 and A1 and A1 in Additional files 11 and 14: Figures S8 and S11) must have occurred in the ancestral amniote lineage as three types of paralogous lysozyme *g* genes (*LygA*, *LygB*, and *LygC*) are found in birds and reptiles. In contrast to birds and reptiles, only one (*LygA*) of these three types of paralogous lysozyme *g* genes was retained on the mammalian lineage (Figure 2 and Additional files 8 and 14: Figure S8 and S11). Our phylogenetic analysis did not clearly resolve the order of the two duplication events, likely due to the short time between the duplications, but strongly indicate that both duplications occurred prior to the divergence of the avian, reptilian and mammalian classes. In agreement with previous analysis [29] a duplication (labeled M in Figure 2 and Additional files 11, 12, and 14: Figures S8, S9, and S11) of the lysozyme *g* gene occurred in an early common ancestor of mammals. Phylogenetic analysis suggests that this duplication occurred prior the divergence of marsupial and placental mammals, but after divergence of mammals from reptiles (Figure 2 and Additional file 11: Figure S8). Considerable divergence between the *LygA1* and *LygA2* forms of the mammalian lysozyme *g* genes exists, as illustrated by the phylogenetic analysis (Figure 2 and Additional files 11, 12, and 14: Figures S8, S9, and S11), and sequence comparisons suggest that the partial platypus lysozyme *g* gene sequence is most similar to the mammalian *LygA2* sequences (data not shown), which would be consistent with a duplication of the lysozyme *g* gene occurring soon after the divergence of mammals and reptiles. An ancient duplication (labeled F in Figure 2 and Additional files 11 and 13: Figures S8 and S10) is also inferred in fish. The duplication of the fish lysozyme *g* gene was likely not due to the fish-specific genome duplication [47], as this duplication event is nested within teleost fish and species such as salmon and zebrafish diverged prior to the duplication of the lysozyme *g* gene (Figure 2 and Additional files 11 and 13: Figures S8 and S10). Gene products of all three ancient amplification events have been retained in multiple species suggesting that these gene duplications lead to lysozyme *g* proteins that have been retained for different functions, however, there have also been some notable gene losses.

### Loss of lysozyme *g* genes

While all three gene products of the ancient amniote gene duplications (*LygA*, *LygB*, and *LygC*) have been retained in diverse reptiles and birds, no sequences similar to the *LygB* or *LygC* genes were found in mammals (Figure 2 and Additional files 1, 2, and 11: Tables S1 and S2 and Figure S8). *LygB* encodes lysozyme *g* protein sequences that were initially identified in the eggs of birds [9,11]. As mammals do not lay eggs, this gene may not be necessary and could have been lost. The functions of *LygA* and *LygC* are unknown, thus the consequence of the loss of *LygC* in mammals is unclear, but the duplication of the *LygA* gene on the mammalian lineage may represent a potential compensation for the loss of this gene. The Mammalian *LygA1* and *LygA2* genes are maintained in most mammals, but recently the *LygA1* gene has been pseudogenized in a number of artiodactyl species (see Additional file 3: Figure S1). Whether the loss of *LygA1* in artiodactyls is associated with the amplification of the lysozyme *c* (Lyz) gene in many of these species [9,12] is an intriguing possibility that needs further investigation.

The fish-specific *LygF2* paralog was found in only a few species, while the *LygF1* paralog was retained in a large number of fish species (Additional files 1 and 2: Tables S1 and S2). Only 2 full-length *LygF2* sequences (medaka *LygF2* and tertaodon *LygF2*) were found (Figure 2 and Additional files 11 and 13: Figures S8 and S10), although the partial takifugu *LygF2* sequence also likely belongs to this clade based on sequence similarity and shared genomic neighborhoods (results not shown). The differential loss of the *LygF1* and *LygF2* genes suggests that these two genes have different functions, with the *LygF1* gene having a near essential function, thus preventing loss, while the *LygF2* gene has a function that is not universally essential that can be lost.

### Evolution of lysozyme *g* function

The observation of relatively ancient duplications of the lysozyme *g* genes in the ancestral amniote lineage parallels the duplications of the lysozyme *c*-like genes that predate mammalian radiation [12]. Duplication of the lysozyme *c* gene resulted in proteins that now have very different functions [8–12] raising the possibility that a similar diversification of lysozyme *g* protein function may have also occurred. Lysozyme *g* was first identified as an anti-bacterial enzyme in the eggs of birds, where it presumably helps protect against bacterial infection [21,22]. To confer this activity, lysozyme *g* is secreted from cells and possesses a catalytic active site allowing the cleavage of peptidoglycan [9,11], although a non-enzymatic antibacterial activity has also been described for fragments of the goose lysozyme *g* protein sequence [48]. As previously noted, some lysozyme *g* sequences (e.g., many fish *LygF1* sequences and chicken *LygC* (this lysozyme is named *g*2 in [31]) do not have classical signal peptides [29–31]. We used the SignalP 4.1 server [49] to predict signal peptides in our lysozyme *g* sequences. As expected, most fish lysozyme *g* sequences lack a signal peptide, as did the chicken lysozyme *LygC* sequence (*Lyg2* in [31]) (Additional file 15: Table S4). *LygC* orthologs produced by the amnoite lineage duplication (duplication A in Figure 2) lack signal peptides, as do the two coelacanth paralogs (Additional file 15: Table S4). Lack of a signal peptide does not necessarily prevent secretion, as proteins can be secreted using a non-classical pathway [50], a pathway that may be used by the chicken lysozyme *g* isoform that does not have the signal peptide [31]. To examine the possibility that lysozyme *g* sequences that lack a signal peptide use the non-classical pathway we used the Secretome 2.0 server [51] to examine their secretion potential. While the chicken *LygC* lysozyme, was suggested to have the potential to be secreted by the non-classical pathway, only a few other lysozyme *g* sequence (e.g., Chinese soft-shelled turtle *LygB3*, platyfish *LygF1*, tongue sole *LygF1*, cod *LygF1b*, tetraodon *LygF2*, and takigufu *LygF1b*) were suggested to potentially use this pathway, while the majority of lysozyme *g* sequences that lack a signal peptide did not obtain scores consistent with secretion (Additional file 15: Table S4). This result would suggest that most, if not all, of the lysozyme *g* sequences that lack signal peptides are not secreted, but does not exclude the possibility that they have intracellular antibacterial function.

Some members of the mammalian lysozyme *c* gene family potentially have lost their bacteriolytic activity, as they do not have muramidase enzymatic activity that cleaves the glycosidic bonds in peptidoglycan in bacterial cell walls [8–12]. The best-characterized example of a mammalian lysozyme gene family that has lost antibacterial function is lactalbumin, a protein that now has an essential function in lactose formation [8,10]. Several other members of the mammalian lysozyme *c* gene family have changes in the active site amino acid residues that should prevent enzymatic function against glycosidic bonds, thus are also suggested to have novel functions [12,52,53]. Despite showing limited sequence similarity, lysozyme *g* shares a similar muramidase catalytic mechanism with that of lysozyme *c* [11,14,19,54]. Crystal structures of goose lysozyme *g* identified glutamate residue 73 (numbering from the mature goose sequence) as being the catalytic sites [14,19], which was confirmed by site-directed mutagenesis [54]. Glutamate 73 is analogous to glutamate 35 in chicken lysozyme *c*, with aspartic acid residues at sites 86 or 97 (in the goose sequence) suggested to be analogs of aspartate 52 of chicken lysozyme *c* [14,19]. Structural [55] and site-directed mutagenesis [56] studies suggest that aspartate-97 is the most likely analog. The results of the site-directed mutagenesis study might suggest that aspartate-86 can replace aspartate-97 if it is changed [56]. Earlier studies have identified a few lysozyme *g* sequences that have changes in the active site residues that likely prevent muramidase activity [11,12], suggesting, like the lysozyme *c* paralogs, that some lysozyme *g* proteins have additional non-bacteriolytic functions, although non-enzymatic bacteriolytic function [48] has not been excluded.

Our alignments of lysozyme *g* protein sequences from diverse vertebrate species identify a large number of sequences that have changes at the putative active site residues (Additional file 15: Table S4). A few of these changes may reflect sequencing errors, since they come from draft genome sequences, but many of the mutations are shared among phylogenetic closely related species strongly suggesting that these are true evolutionary changes. A total of 28 of the 181 sequences had changes at the position orthologous to glutamate-73 in the goose sequence, where 8 of those replacements were to glutamine, which has been shown by site-directed mutagenesis to abolish activity, and only one possesses aspartate, which was shown to dramatically reduce activity [54]. The majority of the remaining variants have lysine or glycine replacements (Additional file 15: Table S4), which were not tested by site-directed mutagenesis [54], but likely abolish activity. Intriguingly, all but one of the species that possesses a lysozyme *g* that has a mutation at glutamate-73 has at least one additional lysozyme *g* gene, one that has glutamate-73 in their sequence (Additional file 15: Table S4). The one exception is the tit; a bird that likely has additional uncharacterized genes. Single amino acid replacement at either aspartate-86 or −97 (goose numbering) may not fully abolish muramidase activity, as was shown by site-directed mutagenesis [56], but likely will reduce enzymatic activity. Of the 22 and 24 sequences that have replacements at sites orthologous to aspartate-86 and −97, respectively, only 7 had both sites replaced (Additional file 15: Table S4), and thus these seven enzymes have likely completely lost all enzymatic activity. As was seen for glutamate-73, all seven sequences (mouse *LygA1*, rat *LygA1a* and *LygA1b*, opossum *LygA1*, Tasmanian devil *LygA1*, medaka *LygF2*, and tetraodon *LygF2*) that lost both aspartic acid residues (86 and 97) are in species that have two (or more) lysozyme *g* genes, and each of these species retains a copy of the gene that has intact active site residues. Of these seven sequences with mutations at aspartate-86 and −93, three (Tasmanian devil *LygA1*, medaka *LygF2*, and tetraodon *LygA2*) also have mutations at glutamate-73 (Additional file 15: Table S4). While a large number of lysozyme *g* sequences likely do not have muramidase activity, due to loss of key active site residues, they likely all exist within species that retain a lysozyme *g* that posses intact active site residues. These results suggest that lysozyme *g* muramidase activity is likely essential in most, if not all, vertebrates, but that duplicate copies of this enzyme can loose muramidase activity and are retained for an unknown function.

## Conclusions

**Figure 3:**
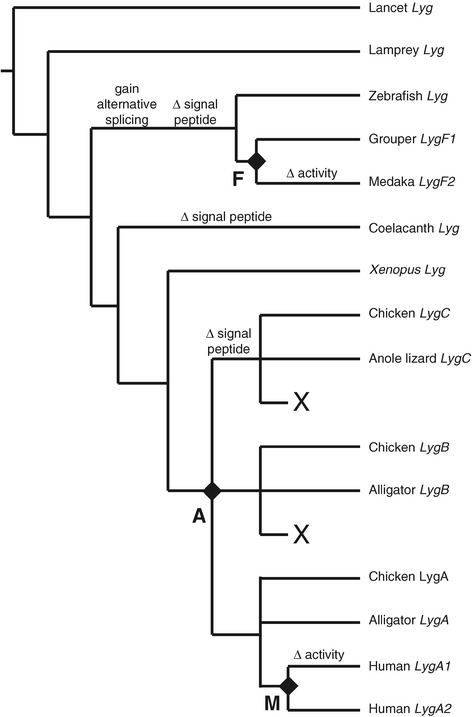
**Evolution of vertebrate lysozyme** ***g*** **sequences.** Schematic model of the key evolutionary events in the evolution of vertebrate lysozyme *g* genes. Phylogeny of representative lysozyme *g* sequences is based on Figure 2. Gene duplication events, indicated by diamonds, which occurred in the amniote ancestor, on the early mammalian lineage, and an early teleost lineage are labeled A, M, and F, respectively. Large Xs refer to gene loss on the mammalian lineage after the gene duplications in the amniote ancestor. Loss of the signal peptide is indicated by “Δ signal peptide” and the loss of muramidase activity by “Δ activity”. Loss of the signal peptide in fish is associated with the gain of alternative splicing in these species.

Our survey of lysozyme *g* sequences in diverse vertebrate species shows that this gene is well conserved and there likely is a lysozyme *g* with antibacterial muramidase activity in almost all vertebrate species, indicating that it must have a very important function. Duplication of the lysozyme *g* gene has been an ongoing process, with multiple parallel duplication of the gene (Figures 1 and 2). While the majority of duplications have been relatively recent (or lineage specific) several more ancient events appear to have led to the evolution of new functions for lysozyme *g*. In Figure 3 we illustrate the key events in the evolution of lysozyme *g*. Lysozyme *g* has an ancient origin and is found in both vertebrate and non vertebrate species [11], and we found this gene in all vertebrate classes except cartilaginous fish (Table 1). Ancestrally, lysozyme *g* appears to be a secreted protein, like lysozyme *c* [11], however this property has been lost on several vertebrate lineages, those leading to teleost fish, lobe-finned fish, and one of three paralogs (*LygC*) found in amniotes (Figure 3 and Additional file 15: Table S4). In fish, the loss of the signal peptide seems to be associated with the gain of alternative splicing [30]. It is suggested that the lack of a signal peptide may not prevent secretion of all lysozyme *g* sequences [31], as an alternative secretion may be used [50], the majority of lysozyme *g* sequences that do not have signal peptides do not show evidence of being able to use the non-classical secretion pathway (Additional file 15: Table S4). This suggests that the majority of lysozyme *g* sequences that lack a signal peptide may have an intracellular function.

Loss of muramidase activity, and potentially bacteriolytic activity, is associated with duplication of the lysozyme *g* gene (Figure 3). Relatively old duplications of the lysozyme *g* gene occurred (1) on the lineage leading to the common ancestor or birds, reptiles and mammals, (2) on the early mammalian lineage and (3) on an early teleost lineage (Figures 2 and 3). In two of these three amplification events, the ones on the mammalian and teleost lineages, one of the products of this duplication (*LygA1* in mammals and *LygF2* in fish) acquired mutations that should prevent enzymatic activity (Figure 3 and Additional file 15: Table S4). In fish, only two full length sequences that represent one product of the ancient lysozyme *g* gene duplication were identified, however both had lost all three active site residues (Additional file 15: Table S4), which contrasts to the lysozyme *g* that has antibacterial activity [25–28] that has been retained in diverse array of fish (Figure 2). This observation suggests that the lysozyme *g* that has lost murimadase activity has only been retained by a few species, implying that it had a less important, or is retained by the lysozyme *g* that has enzyme activity. Only a few species require the separation of these two activities. In mammals, *LygA1* has acquired mutations that should prevent muramidase activity in diverse species (Additional file 15: Table S4), yet has been retained by most species (with artiodactyls being a notable exception, see Additional file 3: Figure S1). Again this implies that sub-functionalization of lysozyme *g* may have occurred, with *LygA2* retaining the antibacterial role and *LygA1* retaining the unknown function. Like lysozyme *c* [12], lysozyme *g* likely has roles other than being only an antibacterial enzyme.

## Methods

### Database searches

The molecular sequence databases maintained by the National Center for Biotechnology Information (*NCBI*) [36] were searched in January 2014 for lysozyme *g*-like sequences. We initially searched the database using the *tBLASTn* algorithm [33] using previously characterized human and bird lysozyme *g* sequences as queries. Subsequent *tBLASTn* searches used putative lysozyme *g*-like protein sequences identified in our earlier searches. Similar searches were conducted using the *Ensembl* and *Pre!Ensembl* genome databases [34,35]. We also searched the elephant shark (the sole representative of cartilaginous fish with a genome sequence) genome generated by the Elephant Shark Genome Project [40]. All sequences that had E-scores below 0.01 were examined. Sequences identified by *BLAST* searches were used in reciprocal *BLASTx* searches of the human and chicken proteomes to ensure that their best matches were lysozyme *g*-like sequences. Searches of the *NCBI* nr database identified lysozyme *g* sequences from the American and Chinese alligators that were derived from genomic sequence. These alligator lysozyme *g* coding sequences were used to identify the genomic region encoding these genes from the *NCBI* genome database [36]. Since the anole lizard lysozyme *g* gene was distributed over two genomic scaffolds we also searched for EST sequence data in the *NCBI* UniGene database [41] for cDNA sequences that could link the two genomic sequences. To provide addition support for the existence of a single lysozyme *g* gene in lizards and snakes we also searched for lysozyme *g* genes in the Burmese python genome data [57] maintained in the *NCBI* genome database [36]. Several lysozyme *g* gene sequences were either not annotated, or incorrectly annotated, in the genome databases (see Additional file 1: Table S1). To better annotate these sequences we used previously published methods [12] to predict lysozyme *g*-like genes. Lancet lysozyme *g* sequences, used as outgroups for the phylogenetic analysis (see below), were identified by searches of the *NCBI* database [36]. Genes were named (see Additional files 1 and 2: Tables S1 and S2) to reflect their orthology-paraology relationships, based on phylogenetic analysis (see below) and sequence similarity, with the *LygA*, *LygB*, and *LygC* representing paralogous genes found in diverse amniotes, *LygA1* and *LygA2* being paralogous genes in mammals, and *LygF1* and *LygF2* being paralogous genes found in teleost fish (see results and discussion for details).

To examine genomic neighborhoods near lysozyme *g* genes, genomic comparisons were conducted using *PipMaker* and *MultiPipMaker* [58–60]. Genes neighboring the lysozyme *g*-like genes were identified from the genome assemblies at *Ensembl* [34] and *Pre!Ensembl* [35]. The organization of genes adjacent to the lysozyme *g*-like genes was used to determine whether the genes of interest reside in conserved genomic neighborhoods. Expression data for lysozyme *g* genes was inferred from the *NCBI* UniGene database [41].

Signal peptides were predicted in the protein sequences using the *SignalP* 4.1 server [49]. The potential of a protein to be secreted using the non-classical secretion pathway was predicted using the *SecretomeP* 2.0 server [51].

### Phylogenetic analysis

Phylogenies of vertebrate lysozyme *g*-like gene coding sequences were generated with near full-length lysozyme *g* sequences from diverse vertebrate (see Additional files 1 and 2: Tables S1 and S2) and outgroups (Additional file 2: Table S2). Lysozyme *g*-like coding sequences were aligned using *MAFFT* [42] as implemented at the *Guidance* web server site [43], using default parameters. Similar results were obtained if *Clustal W* [60] was used as the alignment program. DNA sequence alignments were based on codons to retain protein alignments. The reliability of the alignments was examined using *Guidance* [43] and trimmed alignments using sites that had values above the default cut-off of 0.93 were generated.

Phylogenetic trees of the sequences were generated using Bayesian methods with *MrBayes* 3.2 [61], maximum likelihood with *PhyML* [62], and neighbor-joining distance approaches with *MEGA*5.1 [63]. Bayesian trees were generated from coding sequences with *MrBayes* 3.2 using parameters selected by hierarchical likelihood ratio tests with *ModelTest* version 3.8, as implemented on the FindModel server [64,65]. *MrBayes* was run for 2,000,000 generations with four simultaneous Metropolis-coupled Monte Carlo Markov chains sampled every 100 generations. The average standard deviation of split frequencies dropped to less than 0.02 for all analyses. The first 25% of the trees were discarded as burn-in with the remaining samples used to generate the consensus trees. Trace files generated by *MrBayes* were examined by *Tracer* [66] to verify if they had converged. Bootstrapped maximum likelihood trees, 100 replications, were generated with *PhyML* [62] on the *PhyML* webserver [67] using parameters for the substitution model suggested by *ModelTest*. The maximum likelihood search was initiated from a tree generated by *BIONJ* and the best tree was identified after heuristic searches using the nearest neighbor interchange (NNI) algorithm. *MEGA5.1* [63] was used to construct bootstrapped (1000 replications) neighbor-joining distance trees, using either Maximum Composite Likelihood distances for the DNA sequences or JTT distances for the proteins sequences. Similar results were obtained, but with lower confidence (bootstrap or posterior probabilities) intervals if alternative outgroups (e.g., sequences from *Ciona intestinalis* or from bivalves) were used (results not shown).

With respect to orthology-paralogy issues, choice of outgroup, alignment method (*MAFFT* [42] or *Clustal* [68]), or the use of full-length or trimmed (based on *Guidance* scores [43]) alignments had little influence on the key findings of these analyses. Methods that relied on shorter sequences (*i.e*., trimmed alignments or protein sequences) or simpler models of sequence evolution (*i.e*., neighbor-joining or parsimony) tended to yield weaker support for the earlier diverging lineages, but none of our analyses were in significant conflict with the key inferences of the phylogeny presented in Figure 2 or Additional file 11: Figure S8.

## Availability of supporting data

The data set supporting the results of this article is available in the Dryad Digital Repository, http://datadryad.org/review?wfID=32088&token=6693e830-ad1d-4be1-8b72-bc3f6d82f11f [69] and included within the article’s additional files (see Additional file 10: Figure S7).

## Additional files

Additional file 1: Table S1. Genomic locations of lysozyme *g* genes in sequenced genomes from the *Ensembl* database. Additional file 2: Table S2. Accession numbers of lysozyme *g* coding sequences from the NCBI database. Additional file 3: Figure S1. Artiodactyl lysozyme *g*1 genes are pseudogenes. Additional file 4: Figure S2. Segmental duplication of the rat lysozyme *g* genes. Additional file 5: Figure S3. Chinese alligator predicted transcript XM_006026344 contains two lysozyme *g* genes. Additional file 6: Figure S4. Genomic organization near lysozyme *g* genes of representative mammals. Additional file 7: Figure S5. Genomic organization near lysozyme *g* genes of representative bird and reptile species. Additional file 8: Figure S6. Genomic organization near lysozyme *g* genes of representative fish species. Additional file 9: Table S3. EST data in support of expression of lysozyme *g* genes. Additional file 10: Figure S7. Coding sequences for lysozyme *g* from diverse vertebrates. Additional file 11: Figure S8. Phylogeny of vertebrate lysozyme *g* genes. Additional file 12: Figure S9. Phylogeny of mammalian lysozyme *g* genes. Additional file 13: Figure S10. Phylogeny of fish lysozyme *g* genes. Additional file 14: Figure S11. Phylogeny of tetrapod lysozyme *g* genes. Additional file 15: Table S4. Secretion and conservation of active sites in lysozyme *g* sequences.

## References

[1] Karplus M, Post CB, Jollès P (1996). Simulations of Lysozyme: Internal Motions and the Reaction Mechanism. Lysozymes: Model Enzymes in Biochemistry Andmolecular Biology.

[2] Fukamizo T (2000). Chitinolytic enzymes: catalysis, substrate binding, and their application. Curr Protein Pept Sci.

[3] Strynadka NC, James MN, Jollès P (1996). Lysozyme: A Model Enzyme in Protein Crystallography. Lysozymes: Model Enzymes in Biochemistry and Molecular Biology.

[4] Matagne A, Dobson CM (1998). The folding process of hen lysozyme: a perspective from the ‘new view’. Cell Mol Life Sci.

[5] Merlini G, Bellotti V (2005). Lysozyme: a paradigmatic molecule for the investigation of protein structure, function and misfolding. Clin Chim Acta.

[6] Bonifer C, Huber MC, Faust N, Sippel AE (1996). Regulation of the chicken lysozyme locus in transgenic mice. Crit Rev Eukaryot Gene Expr.

[7] Bonifer C, Jägle U, Huber MC (1997). The chicken lysozyme locus as a paradigm for the complex developmental regulation of eukaryotic gene loci. J Biol Chem.

[8] McKenzie HA, White FH (1991). Lysozyme and alpha-lactalbumin: structure, function, and interrelationships. Adv Protein Chem.

[9] Prager EM, Jollès P, Jollès P (1996). Animal Lysozymes *c* and *g*: An Overview. Lysozymes: Model Enzymes in Biochemistry and Molecular Biology.

[10] Qasba PK, Kumar S (1997). Molecular divergence of lysozymes and alpha-lactalbumin. Crit Rev Biochem Mol Biol.

[11] Callewaert L, Michiels CW (2010). Lysozymes in the animal kingdom. J Biosci.

[12] Irwin DM, Biegel JM, Stewart CB (2011). Evolution of the mammalian lysozyme gene family. BMC Evol Biol.

[13] Jollès P, Jollès J (1984). What’s new in lysozyme research? Always a model system, today as yesterday. Mol Cell Biochem.

[14] Weaver LH, Grütter MG, Remington SJ, Gray TM, Isaacs NW, Matthews BW (1984–1985). Comparison of goose-type, chicken-type, and phage-type lysozymes illustrates the changes that occur in both amino acid sequence and three-dimensional structure during evolution. J Mol Evol.

[15] Van Herreweghe JM, Michiels CW (2012). Invertebrate lysozymes: diversity and distribution, molecular mechanism and in vivo function. J Biosci.

[16] Beintema JJ, Terwisscha Van Scheltinga AC, Jollès P (1996). Plant Lysozymes. Lysozymes: Model Enzymes in Biochemistry and Molecular Biology.

[17] Höltje JV, Jollès P (1996). Bacterial Lysozymes. Lysozymes: Model Enzymes in Biochemistry and Molecular Biology.

[18] Fischetti VA (2005). Bacteriophage lytic enzymes: novel anti-infectives. Trends Microbiol.

[19] Grütter MG, Weaver LH, Matthews BW (1983). Goose lysozyme structure: an evolutionary link between hen and bacteriophage lysozymes?. Nature.

[20] Monzingo AF, Marcotte EM, Hart PJ, Robertus JD (1996). Chitinases, chitosanases, and lysozymes can be divided into procaryotic and eucaryotic families sharing a conserved core. Nat Struct Biol.

[21] Canfield RE, McMurry S (1967). Purification and characterization of a lysozyme from goose egg white. Biochem Biophys Res Commun.

[22] Prager EM, Wilson AC, Arnheim N (1974). Widespread distribution of lysozyme *g* in egg white of birds. J Biol Chem.

[23] Hindenburg A, Spitznagel J, Arnheim N (1974). Isozymes of lysozyme in leukocytes and egg white: evidence for the species-specific control of egg-white lysozyme synthesis. Proc Natl Acad Sci U S A.

[24] Nakano T, Graf T (1991). Goose-type lysozyme gene of the chicken: sequence, genomic organization and expression reveals major differences to chicken-type lysozyme gene. Biochim Biophys Acta.

[25] Hikima J, Minagawa S, Hirono I, Aoki T (2001). Molecular cloning, expression and evolution of the Japanese flounder goose-type lysozyme gene, and the lytic activity of its recombinant protein. Biochim Biophys Acta.

[26] Yin ZX, He JG, Deng WX, Chan SM (2003). Molecular cloning, expression of orange-spotted grouper goose-type lysozyme cDNA, and lytic activity of its recombinant protein. Dis Aquat Organ.

[27] Sakai M, Kono T, Savan R (2005). Identification of expressed genes in carp (*Cyprinus carpio*) head kidney cells after in vitro treatment with immunostimulants. Dev Biol (Basel).

[28] Zheng W, Tian C, Chen X (2007). Molecular characterization of goose-type lysozyme homologue of large yellow croaker and its involvement in immune response induced by trivalent bacterial vaccine as an acute-phase protein. Immunol Lett.

[29] Irwin DM, Gong Z (2003). Molecular evolution of vertebrate goose-type lysozyme genes. J Mol Evol.

[30] Kyomuhendo P, Myrnes B, Nilsen IW (2007). A cold-active salmon goose-type lysozyme with high heat tolerance. Cell Mol Life Sci.

[31] Nile CJ, Townes CL, Michailidis G, Hirst BH, Hall J (2004). Identification of chicken lysozyme *g*2 and its expression in the intestine. Cell Mol Life Sci.

[32] Nilsen IW, Myrnes B, Edvardsen RB, Chourrout D (2003). Urochordates carry multiple genes for goose-type lysozyme and no genes for chicken- or invertebrate-type lysozymes. Cell Mol Life Sci.

[33] Altschul SF, Madden TL, Schäffer AA, Zhang J, Zhang Z, Miller W, Lipman DJ (1997). Gapped BLAST and PSI-BLAST: a new generation of protein database search programs. Nucleic Acids Res.

[34] ᅟ: *Ensembl Genome Browser.* ᅟ [http://www.ensembl.org/index.html]

[35] ᅟ: *Ensembl Pre-release Genome Browser.* ᅟ [http://pre.ensembl.org/index.html]

[36] ᅟ: *National Center for Biotechnology Information.* ᅟ [http://www.ncbi.nlm.nih.gov/]

[37] Bailey JA, Eichler EE (2006). Primate segmental duplications: crucibles of evolution, diversity and disease. Nat Rev Genet.

[38] Meyer A, Wilson AC (1990). Origin of tetrapods inferred from their mitochondrial DNA affiliation to lungfish. J Mol Evol.

[39] Gorr T, Kleinschmidt T, Fricke H (1991). Close tetrapod relationships of the coelacanth Latimeria indicated by haemoglobin sequences. Nature.

[40] Venkatesh B, Lee AP, Ravi V, Maurya AK, Lian MM, Swann JB, Ohta Y, Flajnik MF, Sutoh Y, Kasahara M, Hoon S, Gangu V, Roy SW, Irimia M, Korzh V, Kondrychyn I, Lim ZW, Tay BH, Tohari S, Kong KW, Ho S, Lorente-Galdos B, Quilez J, Marques-Bonet T, Raney BJ, Ingham PW, Tay A, Hillier LW, Minx P, Boehm T (2014). Elephant shark genome provides unique insights into gnathostome evolution. Nature.

[41] ᅟ: *NCBI UniGene Database.* ᅟ [http://www.ncbi.nlm.nih.gov/unigene]

[42] Katoh K, Misawa K, Kuma K, Miyata T (2002). MAFFT: a novel method for rapid multiple sequence alignment based on fast Fourier transform. Nucleic Acids Res.

[43] Penn O, Privman E, Ashkenazy H, Landan G, Graur D, Pupko T (2010). GUIDANCE: a web server for assessing alignment confidence scores. Nucleic Acids Res.

[44] Huelsenbeck JP, Ronquist F, Nielsen R, Bollback JP (2001). Bayesian inference of phylogeny and its impact on evolutionary biology. Science.

[45] Felsenstein J (1981). Evolutionary trees from DNA sequences: a maximum likelihood approach. J Mol Evol.

[46] Cameron CB, Garey JR, Swalla BJ (2000). Evolution of the chordate body plan: new insights from phylogenetic analyses of deuterostome phyla. Proc Natl Acad Sci U S A.

[47] Meyer A, Van de Peer Y (2005). From 2R to 3R: evidence for a fish-specific genome duplication (FSGD). Bioessays.

[48] Thammasirirak S, Pukcothanung Y, Preecharram S, Daduang S, Patramanon R, Fukamizo T, Araki T (2010). Antimicrobial peptides derived from goose egg white lysozyme. Comp Biochem Physiol.

[49] Petersen TN, Brunak S, Von Heijne G, Nielsen H (2011). SignalP 4.0: discriminating signal peptides from transmembrane regions. Nat Methods.

[50] Nickel W (2010). Pathways of unconventional protein secretion. Curr Opin Biotechnol.

[51] Bendtsen JD, Jensen LJ, Blom N, Von Heijne G, Brunak S (2004). Feature-based prediction of non-classical and leaderless protein secretion. Protein Eng Des Sel.

[52] Mandal A, Klotz KL, Shetty J, Jayes FL, Wolkowicz MJ, Bolling LC, Coonrod SA, Black MB, Diekman AB, Haystead TA, Flickinger CJ, Herr JC (2003). SLLP1, a unique, intra-acrosomal, non-bacteriolytic, *c* lysozyme-like protein of human spermatozoa. Biol Reprod.

[53] Zhang K, Gao R, Zhang H, Cai X, Shen C, Wu C, Zhao S, Yu L (2005). Molecular cloning and characterization of three novel lysozyme-like genes, predominantly expressed in the male reproductive system of humans, belonging to the *c*-type lysozyme/alpha-lactalbumin family. Biol Reprod.

[54] Kawamura S, Ohno K, Ohkuma M, Chijiiwa Y, Torikata T (2006). Experimental verification of the crucial roles of Glu73 in the catalytic activity and structural stability of goose type lysozyme. J Biochem.

[55] Hirakawa H, Ochi A, Kawahara Y, Kawamura S, Torikata T, Kuhara S (2008). Catalytic reaction mechanism of goose egg-white lysozyme by molecular modelling of enzyme-substrate complex. J Biochem.

[56] Helland R, Larsen RL, Finstad S, Kyomuhendo P, Larsen AN (2009). Crystal structures of g-type lysozyme from Atlantic cod shed new light on substrate binding and the catalytic mechanism. Cell Mol Life Sci.

[57] Castoe TA, De Koning AP, Hall KT, Card DC, Schield DR, Fujita MK, Ruggiero RP, Degner JF, Daza JM, Gu W, Reyes-Velasco J, Shaney KJ, Castoe JM, Fox SE, Poole AW, Polanco D, Dobry J, Vandewege MW, Li Q, Schott RK, Kapusta A, Minx P, Feschotte C, Uetz P, Ray DA, Hoffmann FG, Bogden R, Smith EN, Chang BS, Vonk FJ (2013). The Burmese python genome reveals the molecular basis for extreme adaptation in snakes. Proc Natl Acad Sci U S A.

[58] ᅟ: *PipMaker and MultiPipMaker.* ᅟ [http://pipmaker.bx.psu.edu/pipmaker/]

[59] Schwartz S, Zhang Z, Frazer KA, Smit A, Riemer C, Bouck J, Gibbs R, Hardison R, Miller W (2000). PipMaker–a web server for aligning two genomic DNA sequences. Genome Res.

[60] Schwartz S, Elnitski L, Li M, Weirauch M, Riemer C, Smit A, Green ED, Hardison RC, Miller W, NISC Comparative Sequencing Program (2003). MultiPipMaker and supporting tools: alignments and analysis of multiple genomic DNA sequences. Nucleic Acids Res.

[61] Ronquist F, Teslenko M, van der Mark P, Ayres DL, Darling A, Höhna S, Larget B, Liu L, Suchard MA, Huelsenbeck JP (2012). MrBayes 3.2: efficient Bayesian phylogenetic inference and model choice across a large model space. Syst Biol.

[62] Guindon S, Dufayard JF, Lefort V, Anisimova M, Hordijk W, Gascuel O (2010). New algorithms and methods to estimate maximum-likelihood phylogenies: assessing the performance of PhyML 3.0. Syst Biol.

[63] Tamura K, Peterson D, Peterson N, Stecher G, Nei M, Kumar S (2011). MEGA5: molecular evolutionary genetics analysis using maximum likelihood, evolutionary distance, and maximum parsimony methods. Mol Biol Evol.

[64] Posada D, Crandall KA (2001). Selecting the best-fit model of nucleotide substitution. Syst Biol.

[65] ᅟ: *Find Model Server.* ᅟ [http://www.hiv.lanl.gov/content/sequence/findmodel/findmodel.html]

[66] Rambaut A, Drummond AJ: *MCMC Trace Analysis Package, Version 1.5.* ᅟ [http://tree.bio.ed.ac.uk/software/tracer/]

[67] ᅟ: *PhyML 3.0: new Algorithms, Methods and Utilities.* ᅟ [http://www.atgc-montpellier.fr/phyml/]

[68] Thompson JD, Higgins DG, Gibson TJ (1994). CLUSTAL W: improving the sensitivity of progressive multiple sequence alignment through sequence weighting, position-specific gap penalties and weight matrix choice. Nucleic Acids Res.

[69] Irwin DM: **Data from: evolution of the vertebrate goose-type lysozyme gene family.** *Dryad Reposit* ᅟ, **ᅟ:**ᅟ [doi:10.5061/dryad.681sn] 10.1186/s12862-014-0188-x PMC4243810 25167808

