# Supplementary material for: Evolution of the vertebrate goose-type lysozyme gene family
Source: BMC Evol Biol. 2014 Aug 29;14:188. doi: 10.1186/s12862-014-0188-x (PMC4243810; doi:10.1186/s12862-014-0188-x)
Supplement: Additional file 3: Figure S1. — Artiodactyl lysozyme g1 genes are pseudogenes. [file 12862_2014_188_MOESM3_ESM.pdf]

```
Dog_gA1     ATG TCT GTG CTG TGG CTG CTT CTG GGG CTT CTG GCC CTT ACT GAT TCA TCT GAA AGC AGC AAC TGG GGA TGC TAT GGA AAC ATC CGA AAC
Cow_gA1     ... ... ... ... ... ... ... ... ..C ..C ..T .T. ... ..A .C. ... ..G ... ... ... .GT ... ... ... .G. ... G.. ... T.G GC.
Sheep_gA1   ... ... ... ..A ... ... ... ..A ..C ..C ..T .T. ... ..A .XG ... ..A ... ... ... .GT ... ... ... CG. ... G.. ... ..G .C.
pig_gA1     ... ... ... ... ... ... ... ... A.A A.C ..C ... ... CT. ..C ... ..A ... ... ... .G. A.. ... ... ... ... G.. .AG .AG .C.

Dog_gA1     GTT GAG ACC CCT GGG GCG TCC TGT GGG ATT GGC AAG CGT CAT GGC CTG AAC TAC TGT GGA GTT CGT GCT TCT GAA AGG CTG GCT GAA ATA
Cow_gA1     C.C C.C ... ... ... ... ..T ... ..C ..C ..A .G. ... ..A ... ... .G. CT. ..C A.. ... ... ... ... ... ... ... ... ... ..T
Sheep_gA1   C.C C.C ... ... ... .T. ..T ... ..C ..C ..A .G. ... ..A ... ... .G. ... ..C ... ... .A. ... ... ... ..A ... ... ... ...
pig_gA1     C.G ..C G.. ... .C. ..A ..T G.. A.. ..G C.G .G. ... .CA ... TCC .G. .T. ..C X.. ... T.. ... ... ... ..A .A. ... .T. .C.

Dog_gA1     GAC ATG CCC TAC CTC CTG AGA TAC CAG --- CCG GTG ATT CAC ACT GTT GGC CAG AAG TAC TGT GTG GAT CCT GCA GTG ATC GCT GGT GTC
Cow_gA1     ... ... ..G .C. ... ... ... ..T --- --- --- A.C ..A .GT ... ... ... ... ... ... ..C C.. ..C ... ..T ... ... ... ... ...
Sheep_gA1   ... ... ..G .C. ... ... ... ..T TGA --- ..T A.C ..A ..T ... ... ... ... ... ... ..C C.. ..C ... ..T ... ... A.. ... ...
pig_gA1     ... ... ..A A.. ... ... ... .GT ..A AAX ..C A.. ..G TGT C.A ..C T.. ..A G.. C.. CAC A.. ..C ... ..T ..C ..G ... ... ...

Dog_gA1     TTG TCC AGG GAG TCT CAT GGC AGC AAC GCT ATG GTC AAT GTG GGC AAC ACG GGC AAC --- --- GGC ATC GGG --- --- --- GAC CCT GGT
Cow_gA1     ... ... ... C.. ... ... ... ... ... .TC C.. .C. ... ... .A. GGT GT. ..T G.T --- --- ..A G.. A.. GTG GTA CAG ... ... ...
Sheep_gA1   ... ... ... C.. ... ... ... ... ... .TC C.. .C. ... ... .A. GGT GT. ..T G.T --- --- ..G G.. A.. GTG GTA CAG ... ... ...
pig_gA1     .CA G.. G.. ..A ... ..C A.T G.. ... .TC CA. ... ..C A.. .A. .GT GT. ACT C.T GAG CAX TGA G.. A.X --- --- --- ... .T. ..A

Dog_gA1     TTT TAT GCT CCC ACA TCC TGG ATC AGC GAG TCT CAA GTT TCT CAG ATA ACT GAG GTC CTT ACT GTT AGG ATC AAA GAA ATT CAA AGG AGG
Cow_gA1     C.. ... .TX ... ... ... ... ... ..T ... G.C ..G ... ..C TGA C.G ... ..A ... ..G ... ..A ..A ... ... ... .CC ..G ... ...
Sheep_gA1   C.. ... ..X ... ... ... ... T.. ..T ... G.C ..G ... ..C .GA G.G ... ..A ..A ..G .A. ..A ..A ... ... ... .CC ..G ... ...
pig_gA1     C.. G.. ..X ... ... ... --- X.. ... A.. G.C ..G ..G .TC ..C ..G G.. .C. ... ..G .T. ... ..A ... .G. ... ..C ..G ... .A.

Dog_gA1     TTT CCA ACT TGG ACC TCT GAC CAG CAC CTG AAA GGT GGA CTC TGT GCC TAT GCT GGA GGT CCT GGC TAC ATC AGA AGC AGC CAG GAC CTG
Cow_gA1     ..C ... ..C TAG ... C.. ... ... T.. ... .G. ... ... ... ... ... ..C A.G ... ... ... ... ... ... CA. ... ... ... ... ...
Sheep_gA1   ..C ... ..C TAG ... C.. ... ... T.. ... .G. ... ... ... ... ... ..C A.G ... ... G.. ... ... ... C.. ... ... ... ... ...
pig_gA1     .G. ... ..C ... ... C.. ... ... T.T ... ... ... ..C T.. ... ... ..C AAG ... ... G.. ... A.. ... ... ... ... ... ... ...

Dog_gA1     AGC TGT GAC TTC TGC AAT GAC GTC CTT GCA CGA GCC AAA TAC TTC AAG AGA CAT GGC TTC TAA
Cow_gA1     ... ... ... ... ... ..C ..T ... ... ... .A. ... ..G ... ... ... ... ..A ... ... G..
Sheep_gA1   ... ... ... ... ... ... ..T ... ... ... .A. ... ..G ... ... ... ... ..A ... ... ...
pig_gA1     .A. ... ... ..T G.. ... ..T ... ... ... ... AT. ... ... ... ... ... ..G ... ... ...
```

Figure S1. Artiodactyl *LygA1* genes are pseudogenes. Alignment of predicted cow, sheep and pig *LygA1* sequences with the intact dog *LygA1* open reading frame. Nucleotide differences from the dog sequence are illustrated, with identical bases shown as dots. Xs, in red, refer to gaps that cause frameshifts. Dashes (-) indicate gaps that are in frame. Codons highlighted in red introduce premature stop codons.
