# Supplementary material for: Evolution of the vertebrate goose-type lysozyme gene family
Source: BMC Evol Biol. 2014 Aug 29;14:188. doi: 10.1186/s12862-014-0188-x (PMC4243810; doi:10.1186/s12862-014-0188-x)
Supplement: Additional file 4: Figure S2. — Segmental duplication of the rat lysozyme g genes. [file 12862_2014_188_MOESM4_ESM.pdf]

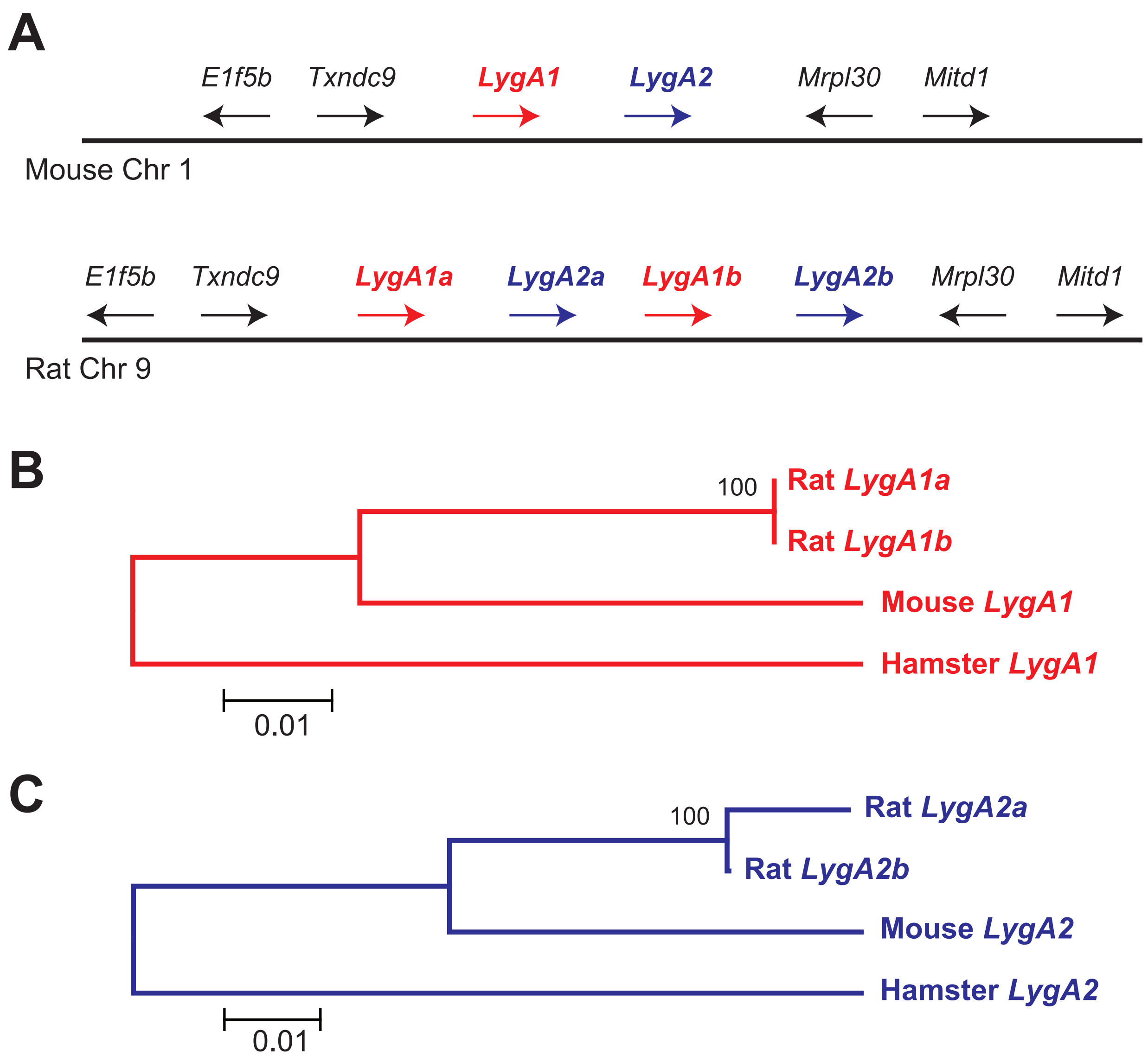

Figure S2. Segmental duplication of the rat lysozyme *g* genes. **A**. Comparison of the genomic neighborhoods of the mouse and rat lysozyme *g* genes. The organization of the mouse genes is similar to that of the human genes (see Fig. 2), while the rat has 4 lysozyme *g* genes at this location. **B** and **C**. Bootstrapped neighbor-joining trees generated from aligned *LygA1* (**B**) and *LygA2* (**C**) coding sequences from mouse, rat, and hamster. Hamster was used to root the tree.
