# Supplementary material for: Evolution of the vertebrate goose-type lysozyme gene family
Source: BMC Evol Biol. 2014 Aug 29;14:188. doi: 10.1186/s12862-014-0188-x (PMC4243810; doi:10.1186/s12862-014-0188-x)
Supplement: Additional file 5: Figure S3. — Chinese alligator predicted transcript XM_006026344 contains two lysozyme g genes. [file 12862_2014_188_MOESM5_ESM.pdf]

**A**

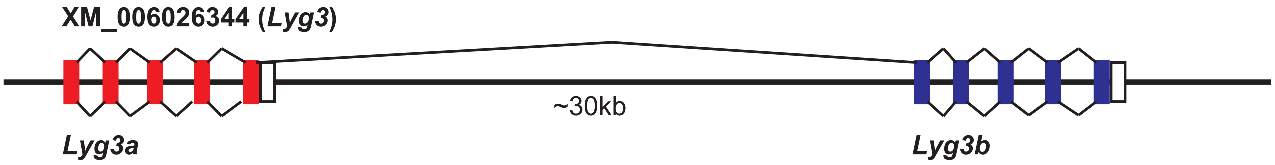


**B**

```
XM_006026344   MHLPLIILGL TAFIDISFSQ SICYRNINGV DTTGASCQTA RSEGLIYCGV PASEKIAEGD
LygB2          MHLPLIILGL TAFIDISFSQ SICYRNINGV DTTGASCQTA RSEGLIYCGV PASEKIAEGD
LygC           ---------- ---------- ---------- ---------- ---------- ----------

XM_006026344   LTRMNKYKGI IKSAGRKLCV DPAVIAGIVS RESHAGAALT HDGWDERRNG FGLMQIDRRS
LygB2          LTRMNKYKGI IKSAGRKLCV DPAVIAGIVS RESHAGAALT HDGWDERRNG FGLMQIDRRS
LygC           ---------- ---------- ---------- ---------- ---------- ----------

XM_006026344   YPQLTRPWNS EAHLIQGTQI LVKMITAIQR KFPRWTKEQQ LKGGISAYNA GPRNVQTYAD
LygB2          YPQLTRPWNS EAHLIQGTQI LVKMITAIQR KFPRWTKEQQ LKGGISAYNA GPRNVQTYAD
LygC           ---------- ---------- ---------- ---------- ---------- ----------

XM_006026344   MDIGTTHNDY ANDVVARAQF RGHISHNELK VFWKHANLSE HYGKLMDVDT TGASIETGKQ
LygB2          MDIGTTHNDY ANDVVARAQF YKKNGY---- ---------- ---------- ----------
LygC           ---------- ------MCRF RGHISHNELK VFWKHANLSE HYGKLMDVDT TGASIETGKQ

XM_006026344   EGLSYGGVTA SEKIAERDLK NLQKYETKIK NVGKKLGVDP ALIAAIISRE SHGGIVLKDG
LygB2          ---------- ---------- ---------- ---------- ---------- ----------
LygC           EGLSYGGVTA SEKIAERDLK NLQKYETKIK NVGKKLGVDP ALIAAIISRE SHGGIVLKDG

XM_006026344   WGDRGNGFGL MQVDKRHHEI VGTWDSEEHI TQGTEILCGM IKDIQKKFPQ WTREQQLKGG
LygB2          ---------- ---------- ---------- ---------- ---------- ----------
LygC           WGDRGNGFGL MQVDKRHHEI VGTWDSEEHI TQGTEILCGM IKDIQKKFPQ WTREQQLKGG

XM_006026344   ISAYNAGPKN IQSYERMDIG TTKNDYANDV VARAKFYKRN GY
LygB2          ---------- ---------- ---------- ---------- --
LygC           ISAYNAGPKN IQSYERMDIG TTKNDYANDV VARAKFYKRN GY
```

**Figure S3. Chinese alligator predicted transcript XM_006026344 contains two lysozyme *g* genes**. **A**. The organization of a lysozyme *g* gene predicted by transcript XM_006026344 is shown above genomic sequence from genomic accession NW_00542190.1 that predicts tandem lysozyme *g* coding sequences. Below are two alternative predicted gene structures that each contains a single lysozyme *g* coding sequence. **B**. Alignment of the full-length protein sequence predicted by XM_006026344 with the two single lysozyme *g* coding sequences (*LygB2* (red) and *LygC* (blue)).
