# Supplementary material for: Evolution of the vertebrate goose-type lysozyme gene family
Source: BMC Evol Biol. 2014 Aug 29;14:188. doi: 10.1186/s12862-014-0188-x (PMC4243810; doi:10.1186/s12862-014-0188-x)
Supplement: Additional file 6: Figure S4. — Genomic organization near lysozyme g genes of representative mammals. [file 12862_2014_188_MOESM6_ESM.pdf]

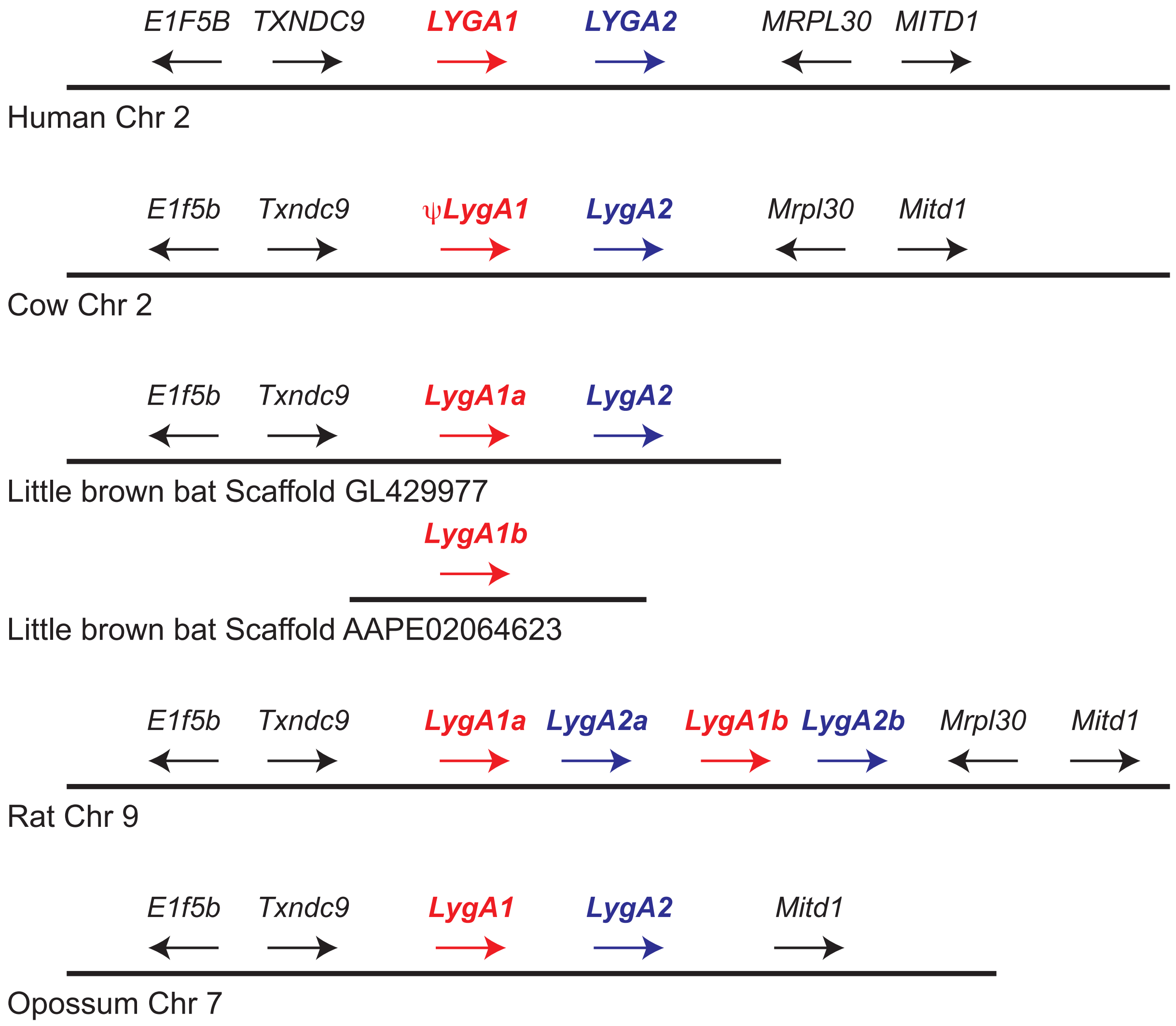

Figure S4. Genomic organization of genes near lysozyme *g* genes of representative mammalian species. The relative organization and orientation of genes near lysozyme *g* genes in representative diverse vertebrate species. Species and chromosomes (or scaffolds) are from Ensembl [34,35]. The Little brown bat lysozyme *g* genes are on two different genomic scaffolds. *LygA1* genes are labeled in red and *LygA2* genes in blue. See Additional files 1 and 2: Tables S1 and S2, for details on genomic locations. Gene sizes and distances between genes are not to scale. Arrowheads indicate direction of transcription. Gene symbols are from Ensembl.
