# Supplementary material for: Evolution of the vertebrate goose-type lysozyme gene family
Source: BMC Evol Biol. 2014 Aug 29;14:188. doi: 10.1186/s12862-014-0188-x (PMC4243810; doi:10.1186/s12862-014-0188-x)
Supplement: Additional file 7: Figure S5. — Genomic organization near lysozyme g genes of representative bird and reptile species. [file 12862_2014_188_MOESM7_ESM.pdf]

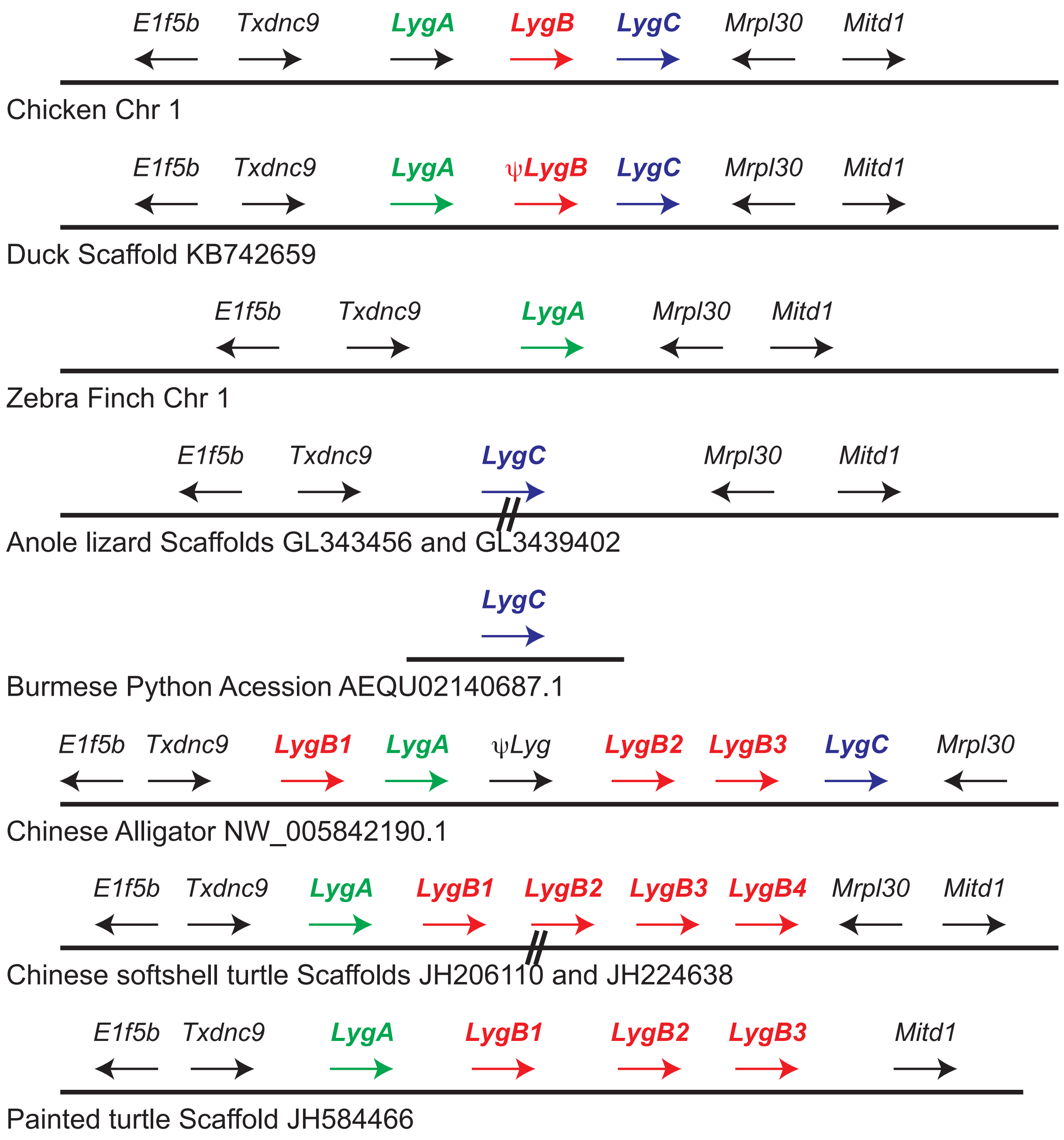

Figure S5. Genomic organization of genes near lysozyme *g* genes of representative bird and reptile species. The relative organization and orientation of genes near lysozyme *g* genes in birds and reptiles. Species and chromosomes (or scaffolds or sequence accessions) are from Ensembl [34,35] or NCBI [36]. The Anole lizard and Chinese softshell turtle genomic neighborhoods are composed of two scaffolds that are likely adjacent. Orthologs of the chicken *LygA* genes are labeled in green, chicken *LygB* genes in red, and chicken *LygC* genes in blue. See Additional files 1 and 2: Tables S1 and S2, for details on genomic locations. Gene sizes and distances between genes are not to scale. Arrowheads indicate direction of transcription. Gene symbols are from Ensembl.
