# Supplementary material for: Evolution of the vertebrate goose-type lysozyme gene family
Source: BMC Evol Biol. 2014 Aug 29;14:188. doi: 10.1186/s12862-014-0188-x (PMC4243810; doi:10.1186/s12862-014-0188-x)
Supplement: Additional file 8: Figure S6. — Genomic organization near lysozyme g genes of representative fish species. [file 12862_2014_188_MOESM8_ESM.pdf]

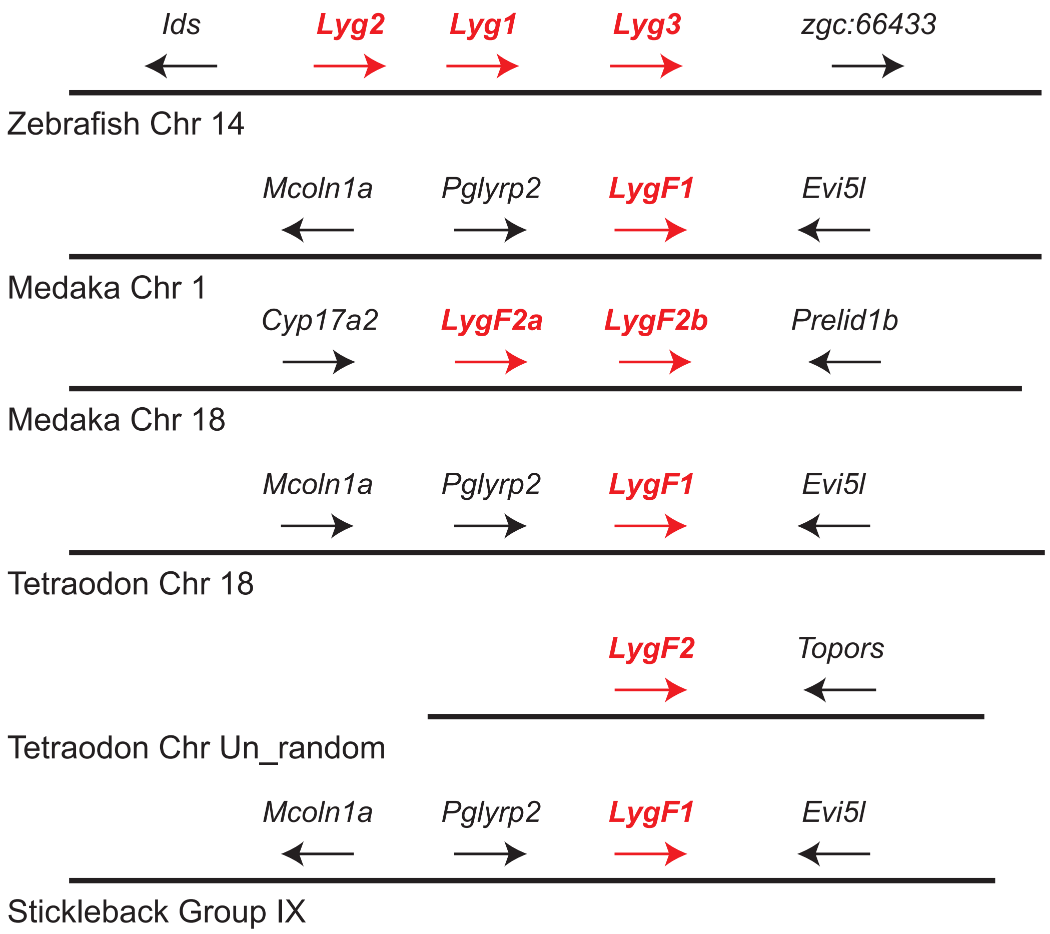

Figure S6. Genomic organization of genes near lysozyme *g* genes of representative fish species. The relative organization and orientation of genes near lysozyme *g* genes in birds and reptiles. Species and chromosomes are from Ensembl [34,35]. The Medaka genomic neighborhoods are composed of two unlinked scaffolds. Lysozyme *g* genes are labeled in red. See Additional files 1 and 2: Tables S1 and S2, for details on genomic locations. Gene sizes and distances between genes are not to scale. Arrowheads indicate direction of transcription. Gene symbols are from Ensembl.
