# Supplementary material for: Evolution of the vertebrate goose-type lysozyme gene family
Source: BMC Evol Biol. 2014 Aug 29;14:188. doi: 10.1186/s12862-014-0188-x (PMC4243810; doi:10.1186/s12862-014-0188-x)
Supplement: Additional file 10: Figure S7. — Coding sequences for lysozyme g from diverse vertebrates. [file 12862_2014_188_MOESM10_ESM.docx]

>Human_LYGA1

ATGTCTGCATTGTGGCTGCTGCTGGGCCTCCTTGCCCTGATGGACTTGTCTGAAAGCAGCAACTGGGGATGCTATGGAAACATCCAAAGCCTGGACACCCCTGGAGCATCTTGTGGGATTGGAAGACGTCACGGCCTGAACTACTGTGGAGTTCGTGCTTCTGAAAGGCTGGCTGAAATAGACATGCCATACCTCCTGAAATATCAACCCATGATGCAAACCATTGGCCAAAAGTACTGCATGGATCCTGCCGTGATCGCTGGTGTCTTGTCCAGGAAGTCTCCCGGTGACAAAATTCTGGTCAACATGGGCGATAGGACTAGCATGGTGCAGGACCCTGGCTCTCAAGCTCCCACATCCTGGATTAGTGAGTCTCAGGTTTCCCAGACAACTGAAGTTCTGACTACTAGAATCAAAGAAATCCAGAGGAGGTTTCCAACCTGGACCCCTGACCAGTACCTGAGAGGTGGACTCTGTGCCTACAGTGGGGGTGCTGGCTATGTCCGAAGCAGCCAGGACCTGAGCTGTGACTTCTGCAATGATGTCCTTGCACGAGCCAAGTACCTCAAGAGACATGGCTTCTAA

>Human_LYGA2

ATGTTATCCTCCGTGGTGTTTTGGGGACTAATTGCCCTCATTGGCACTTCCAGGGGCTCATACCCCTTCAGTCACTCAATGAAGCCTCACCTACATCCACGCCTGTACCACGGCTGCTATGGGGACATCATGACCATGAAGACCTCTGGGGCCACTTGTGATGCAAACAGTGTGATGAACTGCGGGATCCGTGGTTCTGAAATGTTTGCTGAGATGGATTTGAGGGCCATAAAACCTTACCAGACTCTGATCAAAGAAGTCGGGCAGAGACATTGCGTGGACCCTGCTGTCATCGCAGCCATCATCTCCAGGGAAAGCCATGGCGGATCTGTCCTGCAAGACGGCTGGGACCACAGGGGACTTAAATTTGGCTTGATGCAGCTTGATAAACAAACGTACCACCCTGTCGGTGCCTGGGATAGCAAAGAGCACCTTTCACAGGCTACTGGGATTCTAACAGAGAGAATTAAGGCAATCCAGAAAAAATTCCCCACGTGGAGTGTTGCTCAGCACCTCAAAGGTGGTCTCTCAGCTTTTAAGTCAGGAATTGAAGCGATTGCCACCCCATCGGACATAGACAATGACTTCGTCAATGATATCATTGCTCGAGCTAAGTTCTATAAAAGACAAAGCTTCTAG

>Pygmy_Chimpanzee_LygA2

ATGTTATCCTCCGTGGTGTTTTGGGGACTAATTGCCCTCATTGGCACTTCCAGGGGCTCGTACCCCTTCACTCACTCAATGAAGCCTCACCTACATCCACGCCTGTACCATGGCTGCTACGGGGACATCATGACCATGAAGACCTCTGGGGCCACTTGTGATGCAAACAGTGTGATGAACTGCGGGATCCGTGGTTCTGAAATGTTTGCTGAGATGGATTTGAGGGCCATAAAACCTTACCAGACTCTGATCAAAGAAGTCGGGCAGAGACATTGCGTGGACCCTGCTGTCATCGCAGCCATCATCTCCAGGGAAAGCCATGGCGGATCTGTCCTGCAAGACGGCTGGGACCACAGGGGACTTAAATTTGGCTTGATGCAGCTTGATAAACAAACGTACCACCCTGTCGGTGCCTGGGACAGTAAAGAGCACCTTTCACAGGCTACTGGGATTCTAACAGAGAGAATTAAGGCAATCCAGAAAAAATTCCCCACGTGGAGTGTTGCTCAGCACCTCAAAGGTGGTCTCTCAGCTTTTAAGTCAGGAATTGAAGCGATTGCCACCCCATCGGACATAGACAATGACTTTGTCAATGATATCATTGCTCGAGCTAAGTTCTATAAAAGACAAAGCTTCTAG

>Chimpanzee_LygA1

ATGTCTGCATTGTGGCTGCTGCTGGGCCTCCTTGCCCTGATGGACTTGTCTGAAAGCAGCAACTGGGGATGCTATGGAAACATCCAAAGCCTGGACACCCCTGGAGCATCTTGTGGGACTGGAAGACGTCACGGCCTGAACTACTGTGGAGTTCGTGCTTCTGAAAGGCTGGCTGAAATAGACATGCCATACCTCCTGAAATATCAACCCATGATGCAAACCATTGGCCAAAAGTACTGCGTGGATCCTGCCGTGATTGCTGGTGTCTTGTCCAGGAAGTCTCCCGGTGACAAAATTCTGGTCAACATGGGCGATAGGACTAGCATGGTGCAGGACCCTGGCTCTCAAGCTCCCACATCCTGGATTAGTGAGTCTCAGGTTTCCCAGACGACTGAAGTTCTGACTACTAGAATCAAAGAAATCCAGAGGAGGTTTCCAACCTGGACCCCTGACCAGTACCTGAGAGGTGGACTCTGTGCCTACAGTGGGGGTGCTGGCTATGTCCGAAGCAGCCAGGACCTGAGCTGTGACTTCTGCAATGATGTCCTTGCACGAGCCAAGTACCTCAAGAGACACGGCTTCTAA

>Chimpanzee_LygA2

ATGTTATCCTCCGTGGTGTTTTGGGGACTAATTGCCCTCATTGGCACTTCCAGGGGCTCGTACCCCTTCACTCACTCAATGAAGCCTCACCTACATCCACGCCTGTACCATGGCTGCTACGGGGACATCATGACCATGAAGACCTCTGGGGCCACTTGTGATGCAAACAGTGTGATGAACTGCGGGATCCGTGGTTCTGAAATGTTTGCTGAGATGGATTTGAGGGCCATAAAACCTTACCAGACTCTGATCAAAGAAGTCGGGCAGAGACATTGCGTGGACCCTGCTGTCATCGCAGCCATCATCTCCAGGGAAAGCCATGGCGGATCTGTCCTGCAAGACGGCTGGGACCACAGGGGACTTAAATTTGGCTTGATGCAGCTTGATAAACAAACGTACCACCCTGTCGGTGCCTGGGACAGTAAAGAGCACCTTTCACAGGCTACTGGGATTCTAACAGAGAGAATTAAGGCAATCCAGAAAAAATTCCCCACGTGGAGTGTTGCTCAGCACCTCAAAGGTGGTCTCTCAGCTTTTAAGTCAGGAATTGAAGCGATTGCCACCCCATCGGACATAGACAATGACTTTGTCAATGATATCATTGCTCGAGCTAAGTTCTATAAAAGACAAAGCTTCTAG

>Gorilla_LygA1

ATGTCTGCATTGTGGCTGCTGCTGGGCCTCCTTGCCCTGATGGACTTGTCTGAAAGCAGCAACTGGGGATGCTATGGAAACATCCAAACCCTGGACACCCCTGGAGCATCTCGTGGGATTGGAAGACGTCACGGCCTGAACTACTGCGGAGTTCATGCTTCTGAAAGGCTGGCTGAAATAGACATGCCATACCTCCTGAAATATCAACCCATGATGCAAACCATTGGCCAAAAGTACTGCGTGGATCCTGCCATGATCGCTGGTGTCTTGTCCAGGAAGTCTCCCGGTGACAAAATTCTGGTCAACGTGGGCGATAGGACTAGCATGGTGCAGGACCCTGGATCTCATGCTCCCACATCCTGGATTAGTGAGTCTCAGGTTTCCCAGACGACTGAAGTTCTGACTACTAGAATCAAAGAAATCCAGAGGCGGTTTCCAACCTGGACCTCTGACCAGTACCTGAGAGGTGGACTCTGTGCCTACAGTGGGGGTGCTGGCTATGTCCGAAGCAGCCAGGACCTGAGCTGTGACTTCTGCAATGGTGTCCTTGCACGAGCCAAGTACCTCAAGAGACACGGCTTCTAA

>Gorilla_LygA2

ATGTTATCCTCCATGGTGTTTTGGGGACTAATTGCCCTCATTGGCACTTCCAGGGGCTCGTACCCCTTCACTCACTCAATGAAGCCTCACCTACATCCACGCCTGTACCATGGCTGCTACGGGGACATCATGACCATGAAGACCTCTGGGGCCACTTGTGATGCAAACAGTGTGATGAACTGCGGGATCCGTGGTTCTGAAATGTTTGCTGAGATGGATTTGAGGGCCATAAAACCTTACCAGACTCTGATTAAAGAAGTCGGGCAGAGACACTGCGTGGACCCTGCTGTCATCGCAGCCATCATCTCCAGAGAAAGCCATGGCGGATCTGTCCTGCAAGACGGCTGGGACCACAGGGGACTTAAATTTGGCTTGATGCAGCTTGATAAACAAACGTACCACCCTGTCGGTGCCTGGGACAGCAAAGAGCACCTTTCACAGGCTACTGGGATTCTAACAGAGAGAATTAAGGCAATCCAGAAAAAATTCCCCACGTGGAGTGTTGCTCAGCACCTCAAAGGTGGTCTCTCAGCTTTTAAGTCAGGAATTGAAGCGATTGCCACCCCATCGGACATAGACAATGACTTCGTCAATGATATCATTGCTCGAGCTAAGTTCTATAAAAGACAAAGCTTCTAG

>Gibbon_LygA1

ATGTCTGCATTGTGGCTGCTGCTGGGCCTCCTTGCCCTGATGGACTTGTCTGAAAGCAGCAACTGGGGATGCTATGGAAACATCCAAACCCTGGACACCCCTGGAGCATCTTGTGGGATTGGAAGACGTCACGGCCTGAACTACTGTGGAGTTCGTGCTTCTGAAAGGCTGGCTGAAATAGACATGCCATACCTCCTGAAATATCAACCCACGATGCAAACCATTGGCCAAAAGTACTGCGTGGATCCTGCCGTGATCGCTGGTGTCTTGTCCAGGAAGTCTCCCGGTGACAAAATTCTGGTCAACGTGGGCGATAGGACTAGCATGGTGCAGGACCCTGACTCTCATGCTCCCATATCCTGGATTAGTGAGTCTCAGGTTTCCCAGACGACTGAAGTTCTGACTACTAGAATCAAAGAAATCCAGCGGAGGTTTCCAACCTGGACCCCTGACCAGTACCTGAGAGGTGGACTCTGCGCCTACAGTAGGGGTGCTGGCTATGTCCGAAGCAGTCAGGACCTGAGCTGTGACTTCTGCAATGATGTCCTTGCACGAGCCAAGTACCTCAAGAGACACGGCTTCTAA

>Gibbon_LygA2

ATGTTATCCTCTGTGGTGTTTTGGGGACTAATTGCCCTCATTGGAACTTCCAGGGGCTCATACCCCTTCACCCACTCAATGAAGCCTCACCTACATCCACGCCTGTACCACGGCTGCTACGGGGACATTATGACCATGAAGACCTCTGGGGCCACTTGTGATGCAAACAGTGTGATGAACTGCGGGATCCGTGGTTCTGAAATGTTTGCTGAGATGGATTTGAGGGCCATAAAACCTTACCAGACTCTGATCAAAGAAGTCGGGCAGAGACATTGTGTGGACCCTGCTGTCATCGCAGCCATCATCTCCAGGGAAAGCCATGGCGGATCTGTCCTGCAAGATGGCTGGGACCACAGGGGACTTAAATTTGGCTTGATGCAGCTTGATAAACAAACGTACCACCCTGTTGGTGCCTGGGACAGCAAAGAGCACCTTTCACAGGCTACTGGGATTCTAACAGAGAGAATTAAGGCAATCCAGAAAAAATTCCCCACGTGGAGTGTTGCTCAGCACCTCAAAGGTGGTCTCTCAGCTTTTAAGTCAGGAATTGAAGCGATTGCCACCCCATCGGACATAGACAATGACTTCGTCAATGATATCATTGCTCGAGCTAAGTTCTATAAAAGACAAAGCTTTTAG

>Squirrel_Monkey_LygA1

ATGTCTGCATTGTGGCTGCTGCTGGGCCTCCTTGCCCTGGCGGATTTGTCTGAAAGCAGCAACTGGGGATGCTATGGAAACATTCAAACCCTGGACACCCCCGGAGCATCTTGTGGGACTGGAAGACGTCACGGCCTGAACTACTGTGGAGTTCGTGCTTCTGAAAGGCTGGCTGAAATAGACATGCCATACCTCCTGAAATATCAGCCCATGATGCAAACCATTAGCCAAAAGTACTGCATGGATCCTGCTGTGATCGCTGGTGTCTTGTCCAGGAAGTCTCCCGGTGACAACATTCTGGTTAACGTGGGCGATAGGACTAGTGTGGTGCAGGACCCTGGCTCTCATGCTCCCACATCCTGGATTAGTGAGTCTCAGGTTTCTCAAATGACTGAGGTTCTGACTACTAGAATCAAAGAAATCCAGAGGAGGTTTCCAACCTGGACCCCTGACCAGTACTTGAGAGGTGGACTCTGTGCCTACAGTGGGGGTGCTGGCTATGTCCAAAGCAGCCAGGACCTGAGCTGTGACTTCTGCAATGATGTTCTTGCACGAGCCAAGTACCTCAAGAGACATGGCTTCTAA

>Squirrel_Monkey_LygA2

ATGCTGTCCTCTGTGGTGTTCTGGGGACTAATTGCCCTCATTGGCACTTCCAGGGGCTCGTACCCCTTCACTCACTCAATGAACCCTCACCTACATCCGCGCCTTTACCACGGCTGCTACGGGGACATCATGACCATGAAGACCTCTGGAGCCACCTGTGATGCAAACAGTGTGATGAACTGTGGGATCCGTGGTTCTGAAATGTTTGCTGAGATGGATTTGAGGGCCATAAAACCTTACCAGACTCTGATCAAGGAAGTCGGGCAGAAACACTGTGTGGACCCTGCTATCATCGCAGCCATTATCTCCAGGGAAAGCCATGGTGGAGCTGTCCTGCAAGATGGCTGGGACCACAGGGGGCTTAAATTTGGCTTGATGCAGCTTGACAAACAAAAGTACTCCCCTGTTGGTACCTGGGACAGCAAAGAACACCTGTCACAGGCTGCTGGGATTCTAACAGATGGAATTAAGGCAATCCAGAAAAAATTCCCCATGTGGAGTGTGGCTCAGCACCTCAAAGGTGGTCTCTCAGCTTTTAAGTCAGGAGTTGAAGCAATTGCCACTCCGGCAGACATAGACAATGACTTCGTCAATGATATCGTTGCCCGAGCTAAGTTCTATAAACGACAAAGCTTCTAG

>Vervet_monkey_LygA1

ATGTCTGCATTGTGGCTGCTGCTGGGCCTCCTTGCCCTGACGGACTTGTCTGAAAGCAGCAACTGGGGATGCTATGGAAACATCCAAACCCTGGACACCCCTGGAGCATCTTGTGGGATTGGAAGACATCACGGCCTGAACTACTGTGGAGTTCGTGCTTCTGAAAGGCTGGCTGAAATAGACATGCCATACCTCCTGAAACATCAACCCACGATGCAAACCATCGGCCAAAAGTACTGCATGGATCCTGCCGTGATCGCTGGTATCTTGTCCAGGAAGTCTCCTGGTGACAAAATTCTGGTCAATGTGGGCGATAGGACTAGCATGGTGCAGGAACCTGGCTCTCACGCTCCCGCAGACTGGATTAGTGAGTCTCAGGCTTCCCAGATGACTGAAGCTCTGGCTACTAGAATCAAAGAAATCCAGAGGAGGTTTCCAACCTGGAGCCCTGACCAGTACCTGAGAGGTGGACTCTGCGCCTACAGTGGGGGTGCTGGCTATGTCCAAAGCAGCCAGGACCTGAGCTGTGACTTCTGCAATGACGTCCTTGCACGAGCCAAGTACCACAAGAGACATGGCTTC

>Vervet_monkey_LygA2

ATGTTATCCTCTGTGGTGTTTTGGGGACTAATTGCCCTCATTGGCACTTCCAGGGGCTCGTACCCCTTCACCCACTCAATGAACCCTCACCTACATCCGCGCCTGTACCACGGCTGCTACGGGGACATCACGACCATGAAGACCTCTGGGGCCACTTGTGATGCAAACAGTGTGATGAACTGCGGGATCCGTGGTTCTGAAATGTTTGCTGAGATGGATCTGAGGGCCATAAAACCCTACCAGACTCTGATCAAAGAAGTCGGGCAGAGACACTGCGTGGACCCTGCTGTCATTGCAGCCATCATCTCCAGGGAAAGTCATGGCGGATCTGTCCTGCAAGATGGCTGGGACCACAGGGGGCTTAAATTTGGCTTGATGCAGCTTGATAAACAAGCGTACCACCCTGTTGGTGCCTGGGACAGCAAAGAGCATCTTTCACAGGCTACTGGGATTCTAACAGAGAGAATTAAGGCAATCCAGAAAAAATTCCCCACGTGGAGTGCTGCTCAGCACCTCAGAGGTGGTCTCTCAGCTTTTAAGTCAGGAATTGAAGCTATTGCCACCCCAGCGGACATAGACAATGACTTCGTCAATGATATCATTGCCCGAGCTAAGTTCTATAAAAGACAAAGCTTC

>Marmoset_LygA1

ATGCCTGCATTGTGGCTGCTGCTGGGCCTCCTTGCCCTTGTGGATTTGTCTGAAAGCAGCAACTGGGGATGCTATGGAAACATTCAAACCCTGGACACCCCTGGAGCATCTTGTAGGACTGGAAGACGTCATGGCCTGAACTACTGTGGAGTTCGTGCTTCTGAAAGGCTGGCTGAAATAGACATGCCATACCTCCTGAAATATCAGCCCATGATGCAAACCATTAGCCAAAAGTACTGCATGGATCCTGCTGTGATCGCTGGTGTCTTGTCCAGGAAGTCTCCCAGTGACAACATTCTGGTCAACGTGGGCGATAGGACTAGCTTGGTGCAGGACCCTGGCTCTTATGCTCCCACTTCCTGGATTAGTGAGTCTCAGGTTTCTCAGATGACTGAGGTTCTGACTACTAGAATCAAAGAAATCCAGAGGAGGTTTCCAACCTGGACCCCTGACCAGTACCTGAGAGGTGGACTCTGTGCCTACAGTGGGGGTGCTGGCTATGTCCGAAGCAGCCAGGACCTGAGCTGTGACTTCTGCAATGATGTTCTTGCACGAGCCAAGTACCTCAAGAGACATGGCTTCTAA

>Marmoset_LygA2

ATGCTAACTTCTGTGGTGTTCTGGGGACTAATTGCCCTCATTGGCACTTCCAGGGGCTCGTACCCCTTCACTCACTCAATGAACCCTCACCTACATCCGCGCCTATACCACGGCTGCTACGGGGACATCATGACCATGAAGACTTCTGGAGCCACCTGTGATGCAAACAGTGCGATGAACTGTGGGATCCGTGGTTCCGAAATGTTTGCTGAGATGGATTTGAGGGACATAAAACCTTACCAGACTCTGATCAAAGAAGTCGGGCAGAAACATTGTGTGGACCCTGCTATCATCGCAGCCATTATCTCCAGGGAAAGCCATGGCGGAGCTGTCCTGCAAGATGGCTGGGACCACAGGGGGCTTAAATTTGGCTTGATGCAGCTTGACAAACAAAAGTACCCCCCGCTTGGTGCCTGGGACAGCAAAGAACACCTTTCACAGGCTGCTGGGATTCTAACAGATGGAATTAAGGCAATCCAGAAAAAATTCCCCACGTGGAGTGTTGCTCAGCACCTCAAAGGTGGTCTCTCAGCTTTTAAGTCAGGAGTTGAAGCAATTGCCACCCCAGCAGACATAGACAATGACTTCGTCAATGATATCGTTGCCCGAGCTAAGTTCTATAAAAGACAAAGCTTCTAG

>Rhesus_macaque_LygA1

ATGTCTGCATTGTGGCTGCTGCTGGGCCTCCTTGCCCTGACAGACTTGTCTGAAAGCAGCAACTGGGGATGCTATGGAAACATCCAAACCCTGGACACCCCTGGAGCATCTTGTGGGATTGGAAGACATCACGGCCTGAACTACTGTGGAGTTCGTGCTTCTGAAAGGCTGGCTGAAATAGACATGCCATACCTCCTGAAATATCAACCCACGATGCAAACCATCGGCCAAAAGTACTGCGTGGATCCTGCCGTGATCGCTGGTATCTTGTCCAGGAAGTCTCCTGGTGACAAAATTCTGGTCAACGTGGGCGATAGGACTAGCATGGTGCAGGAACCTGGCTCTCACGCTCCCGCATACTGGATTAGTGAGTCTCAGGTTTCCCAGATGACTGAAGCTCTGACTACTAGAATCAAAGAAATCCAGAGGAGGTTTCCAACCTGGACCCCTGACCAGTACCTGAGAGGTGGACTCTGTGCCTACAGTGGGGGTGCTGGCTATGTCCAAAGCAGCCAGGACCTGAGCTGTGACTTCTGCAATGATGTCCTTGCGCGAGCCAAGTACCTCAAGAGACATGGCTTTTAA

>Crab_eating_Macaque_LygA1

ATGTCTGCATTGTGGCTGCTGCTGGGCCTCCTTGCCCTGACAGACTTGTCTGAAAGCAGCAACTGGGGATGCTATGGAAACATCCAAACCCTGGACACCCCTGGAGCATCTTGTGGGATTGGAAGACATCATGGCCTGAACTACTGTGGAGTTCGTGCTTCTGAAAGGCTGGCTGAAATAGACATGCCATACCTCCTGAAATATCAACCCACGATGCAAACCATCGGCCAAAAGTACTGCGTGGATCCTGCCGTGATCGCTGGTATCTTGTCCAGGAAGTCTCCTGGTGACAAAATTCTGGTCAACGTGGGCGATAGGACTAGCATGGTGCAGGAACCTGGCTCTCACGCTCCCGCATACTGGATTAGTGAGTCTCAGGTTTCCCAGATGACTGAAGCTCTGACTACTAGAATCAAAGAAATCCAGAGGAGGTTTCCAACCTGGACCCCTGACCAGTACCTGAGAGGTGGACTCTGTGCCTACAGTGGGGGTGCTGGCTATGTCCAAAGCAGCCAGGACCTGAGCTGTGACTTCTGCAATGATGTCCTTGCGCGAGCCAAGTACCTCAAGAGACATGGCTTTTAA

>Crab_eating_Macaque_LygA2

ATGTTATCCTCTGTGGTGTTTTGGGGACTAATTGCCCTCATTGGCACTTCCAGGGGCTCGTACCCCTTCACCCACTCAATGAACCCTCACCTACATCCGCGCCTGTACCACGGCTGCTACGGGGACATCATGACCATGAAGACCTCTGGGGCCACTTGTGATGCAAACAGTGTGATGAACTGCGGGATCCGTGGTTCTGAAATGTTTGCTGAGATGGATCTGAGGGCCATAAAACCCTACCAGACTCTGATCAAAGAAGTCGGGCAGAGACACTGCGTGGACCCTGCTGTCATTGCAGCCATCATCTCCAGGGAAAGTCATGGCGGATCTGTCCTGCAAGATGGCTGGGACCACAGGGGGCTTAAATTTGGCTTGATGCAGCTTGATAAACAAGCGTACCACCCTGTTGGTGCCTGGGACAGCAAAGAGCATCTTTCACAGGCTACTGGAATTCTAACAGAGAGAATTAAGGCAATCCAGAAAAAATTCCCCACGTGGAGTGCTGCTCAGCACCTCAGAGGTGGTCTCTCAGCTTTCAAGTCAGGAGTTGAAGCGATTGCCACCCCAGTGGACATAGACAATGACTTCGTCAATGATATCATTGCCCGAGCTAAGTTCTATAAAAGACAAAGCTTCTAG

>Baboon_LygA1

ATGTCTGCGTTGTGGCTGCTGCTGGGCCTCCTTGCCCTGATGGACTTGTCTGAAAGCAGCAACTGGGGATGCTATGGAAACATCCAAACCCTGGACACCCCTGGAGCGTCTTGTGGGATTGGAAGACATCACGGCCTCAACTACTGTGGAGTTCGTGCTTCTGAAAGGCTGGCTGAAATAGACATGCCATACCTCCTGAAATATCAACCCACGATGCAAACCGTCGGCCAAAAGTACTGCGTGGATCCTGCCGTGATCGCTGGTATCTTGTCCAGGAAATCTCCTAGTGACAAAATTCTGGTCAACGTGGGTGATAGGACTAGCATGGTGCAGGGACCTGGCTCTCACGCTCCCGCATACTGGATTAGTGAGTCTCAGGTTTCCCAGATGACTGAAGCTCTGACTACTAGAATCAAAGAAATCCAGAGGAGGTTTCCAACCTGGACCCCTGACCAGTACCTGAGAGGTGGACTCTGCGCCTACAGTGGGGGTGCTGGCTATGTCCAAAGCAGCCAGGACCTGAGCTGTGACTTCTGCAATGATGTCCTTGCACGAGCCAAGTACCTCAAGAGACATGGCTTCTAA

>Baboon_LygA2

ATGTTATCCTCTGTGGTGTTTTGGGGACTAATTGCCCTCATTGGCACTTCCAGGGGCTCGTACCCCTTCACCCACTCAATGAACCCTCACCTACATCCGCGCCTGTACCACGGTTGCTACGGGGACATCATGACCATGAAGACCTCTGGGGCCACTTGTGATGCAAACAGTGTGATGAACTGCGGGATCCGTGGTTCTGAAATGTTTGCTGAGATGGATCTGAGGGCCATAAAACCTTACCAGACTCTGATCAAAGAAGTCGGGCAGAGACACTGTGTGGACCCTGCTGTCATTGCAGCCATCATCTCCAGGGAAAGTCATGGCGGATCTGTCCTGCAAGATGGCTGGGACCACAGGGGGCTTAAATTTGGCTTGATGCAGCTTGATAAACAAGCGTACCACCCTGTTGGTGCCTGGGACAGCAAAGAGCATCTTTCACAGGCTACTGGGATTCTAACAGAGAGAATTAAGGCAATCCAGAAAAAATTCCCCACGTGGAGTGCTGCTCAGCACCTCAGAGGTGGTCTCTCAGCTTTTAAGTCAGGAATTGAAGCGATTGCCACCCCAGTGGACATAGACAATGACTTCGTCAATGATATCATTGCCCGAGCTAAGTTCTATAAAAGACAAAGCTTCTAG

>Orangutan_LygA1

ATGTCTGCATTGTGGCTGCTGCTGGGCCTCCTTGCCCTGATGGACTTGTCTGAAAGCAGCAACTGGGGATGCTATGGAAACATCCAAACCCTGGACACCCCCGGAGCATCTTGTGGGACTGGAAGATGTCACGGCCTGAACTACTGTGGAGTTCGTGCTTCTGAAACGCTGGCTGAAATAGACATGCCATACCTCCTGAAATATCAACCCACGATGCAAACCATTGGCCAAAAGTACTGCGTGGATCCTGCCGTGATCGCTCCTGTCTTGTCCAGGAAGTCTCCCGGTGACAAAATTCTGGTCAACATGGGCGATAGGACTAGCATGGTGCAGGACCCTGGCTCTCATGCTCCCACATCCTGGATTAGTGAGTCTCAGGTTTCCCAGATGACTGAAGTTCTGACTACTAGAATCAAAGAAATCCAGAGGAGGTTTCCAACCTGGACCCCTGACCAGTACCTGAGAGGTGGACTCTGCGCCTACAGTGGGGATGGTGGCTATGTCGGAAGCAGCCAGGACCTGAGCTGTGAGTTCTGCAATGATGTCCTTGCACGAGCCAAGTACCTCAGGAGACATGGCTTCTAA

>Orangutan_LygA2

ATGTTATCCTCCGTGGTGTTTTGGGGACTAATTGCCCTCATTGGCACTTCCAGGTGCTCGTACCCCTTCACCCACTCAATGAAGCCTCACCTACATCCGCGCCTGTACCACGGCTGCTACGGGGACATCATGACCATGAAGACCTCTGGGGCCACTTGTGATGCAAACAGTGTGATGAACTGCGGGATCCGTGGTTCTGAAATGTTTGCTGAGATGGATTTGAGGGCCATAAAACCTTACCAGACTCTGATCAAAGAAGTCGGGCAGAGACATTGCGTGGACCCTGCTGTCATCGCAGCCATCATCTCCAGGGAAAGCCACGGTGGATCTGTCCTGCAAGACGGCTGGGATCACAGGGGACTTAAATTTGGCTTGATGCAGCTTGATAAACAAACGTACCACCCTGTTGGTGCCTGGGACAGCAAAGAGCACCTTTCACAGGCTACTGGGATTCTAACAAAGAGAATTAAGGCAATCCAGAAAAAATTCCCCACGTGGAGTGTTGCTCAGCACCTCAAAGGTGGTCTCTCAGCTTTCAAGTCAGGAATTGAAGCGATTGCCACCCCATCGGACATAGACAATGACTTCGTCAATGATATCATTGCTCGAGCTAAGTTCTATAAAAGACAAAGCTTCTAG

>Mouse_lemur_LygA1

ATGTCTGCATTGTGGCTGCTTCTGGGCCTCCTTGCCCTGACTGACTCATCGGAAAGCAGCGACTGGGGATGCTATGGAAACATCCGAACCTTGGTCACCCCTGGGGCATCCTGTGGGATTGGAAGACTCCACGGCCTGAACTACTGTGGAGTTCGTGCTTCTGAAAAGCTGGCTGAAATAGACATGCCATACCTGCTGAAATACCAACCCGTGATGCGAACCGCTGGCCAAAAGTACTGCATGGATCCTGCAGTGATTGCTGGTGTCTTGTCCAGGGAGCTTCAGGGTGGCAACGTTCTGGTCAACGTGGGCAACATGGGCAATAGGATTAAGGTGATCCAGGACGCTGGCTTGTCTGTGCCCACATCCTGGATTAGTGAGTCCCAGGTCTCCCAGAAGACTGAGATTCTTACTACTAGAATCAAAGAAATCCAGAGGAGATTTCCAACCTGGACCCCTGACCAGCACCTGAGAGGTGGACTCTGTGCCTACGGTGGGGGTGCTGGCTATGTCAGAAGCAGCGAGGACCTGAGCTGTGACTTCTGCAATGATGTCCTTGCCCGAGCCAAATACCTCAAGAGACACGGCTTCTGA

>Mouse_lemur_LygA2

ATGCTATCCTCTGTCATGTTTTGGGGACTTATTGCCCTCATTGGCACTTCCAGGGGCTCGTACCCTTTCACTCAGTCAATGAGCCCTCACCTGCAGCCTCGCCTGTACCGTGGCTGCTACGGGGACATCATGACCATGGAGACCTCGGGGGCCGCCTGTGATATAAACAGTTTGATTAGCTGCGGGATCCGTGGTTCTGAAATGTTTGCTGAGATGGATTTGAGGGCCATAAAGCCTTACCAGACTGTGATCAAAGAAGTAGGACAGAGGCATTGCGTCGATCCTGCCATCATTGCGGCCATCATCTCCAGGGAAAGCCATGGCGGAAGTATCCTGAGAGATGGCTGGGACCACAGGGGACTTAAATTTGGCTTGATGCAGCTTGATAAACAAATTTACCACCCTGTTGGTACCTGGGACAGCAAAGAACACCTTTTGCAGGCTGTTGGGGTTCTTACAGACAGAATTAAGGCAATCCAGAAAAAATTCCCCACATGGAACGCAACTCAGCACCTCAAAGGTGGTCTCTCAGCTTTTAGATCAGGAATTGAAGCGATTGGCGCCCCAGAGGACATAGACAATGACTACGTCAATGATGTTCTTGCCCGAGCTCGATTCTATAGAAGACAGGGCTACTAG

>Tree_Shrew_LygA1

ATGTCTGCATTGTGGCTGCTTCTGGGCCTCCTCGCCCTGACTGGCTCATCTGAAAGCAGCAACTGGGGATGCTATGGAAACATCCGGACGGTGGACACCCCTGGAGCGTCCTGTATGATTGGGAGGCGCCATGGCCTGAACTACTGTGGAGTTCGTGCTTCAGAAAGGCTGGCTGAGATCGACATGCCATACCTACTGAGGTTCCAGCCCGTGATCCGCACGGTTGGCCAGAAGTACTGCATGGACCCTGCAGTGATCGCTGGTGTCTTGTCCAGGGAGTCTCCAGGTGGCAACGTCCTGGTCAACGTGGGTGATGGAATTGGGGCAGTGCAGGACTCCAGCTTTTATGCTCCCACATCTTGGATCACTGAGTCCCAGGTTTCCCAGAAGACCGAGGTTCTGACTTCTAAAATCAAGGAAATCCAGAGGAGGTTTCCAACCTGGACCCCTGACCAATGCCTGAGAGGTGGACTCTGTGCCTACGATGGGGGCGTTGGTTATGTCAAAAGCAACCAGGACCTGAGCTGTGACTTCTGTAATGATGTCCTTGCACGAGCTAAATACCTCAAGAGACACGGCTTCTAA

>Tree_Shrew_LygA2

ATGCTACCCTCTGTCGTGTTTTGGGGACTCATTGCCCTCATTGGCACTTCCAGGGGCTCGTACCCTTTCACCCACTCAATGAACCCTCACCTGCATCCTCGCCTATACCACGGCTGCTATGGGGACATCATGACCATGGAGACTTCTGGGGCCACCTGTGACGTGAACAGTTTAATCAGTTGCGGCATCCGTGGTTCTGAAATGTTTGCCGCGATGGATCTGAGGGCCATAAAACCTTACCAGGCTCTGATCAAAGAGGTGGGGCAGAGGCATTGCATCGACCCTGCTATCATTGCAGCCATCATCTCCAGAGAAAGCCACGGCGGAGTCGTCCTGCAACGTGGCTGGGACCACAGAGGACTTAAATTTGGCTTGATGCAGCTTGATAAACAAACTCACCACCCCATTGGTGACTGGGACAGCAAAGAACACCTTTCACAAGCTGTTGGGATTCTAGCAGACAGAATTAAGGCAATCCAGAAAAAATTCCCCACATGGAGTGCGGCTCAACACCTCAAAGGTGGTCTCTCAGCCTATAAGTCAGGAATTGAAGCTATTGTCACCCCTGCGGACATAGACAATGGTTTGGCCAATGATATCATTGCCAGAGCTAAATTCTATAGAAAACATGGCTACTAA

>Dolphin_LygA2

ACTTCTAGGGGCTCATGCTCCTTCACCCTTTCAATGAACCCTCGCCTGCGTTCCTGCCTGTACCGTGGCTGCTATGGTACCATCATGACCATGGAGACCTCTGCTGCTACCTGTGATATCACCGGGGTGATTGCCGGCAGCATCTGTGGTTTTGAAATGTTTGCTGAGATGGATTTGAAGGTCTTCAAGTCTTACATTCTAATCAAAGAAGTCAGGCTGAGGCACTGCATGGACCCTGCTCTCACTGCAGCCATCATTTCCAGAGAAAGCCATGGTGGAACCATCCGGCAAGATGGCTGGGACCACAAAGGACTTAAATTTGGCTTGACGCAGCTTGATAAAAAAAAATATCGACCTGTTGGTACCTGGGACAGCAAAGAACACCTTTTGCAGGCTGTTGGGATTCTCACAGACAGAATTAAAGCAAACCAGAAAAAATTCCCCACGTGGAGTGTGGCTCAATACCTCAAAGGTGGTCTCTCAGGCTTCAAGTCAGGAACTGAAGCCACTGCCACCCCCGCGGACATAGACGATGTCATCAGTGATATTATAGCTCGAGCTAAATTCTATAAGAGACACGGCTTCTAG

>Panda_LygA1

ATGTCTGTGCTGTGGCTGCTTCTGGGTCTTCTTGCCCTTACTGATTCATCTGAAAGCAGCAATTGGGGATGCTATGGAAACATCCGAAACTTTGAGACCCCTGGGGCGTCTTGTGGGATTGGAAAGCGTCATGGCCTGAACTATTGTGGAGTTCGTGCTTCTGAAAGGCTGGCTGAAATAGACATGCCGTACCTCCTGAGATATCAGCCCGTGATTCGTACTGTCGGCCAAAAGTACTGTGTGGACCCTGCAGTGATTGCTGGTGTCTTGTCCAGGGAGTCTCATGGTGGCAACGTTATGGCCAATGTGGGCAACATGGGTGATGGCATCGGGGTTGTGCAGGACCCTGGTCTTTATGCTCCCACATCCTGGATCAGCGAGTCCCAGGTTTCTCAAATAACCGAGGTCCTGACTGTTAGGATCAAAGAAATCCAGAGGAGGTTTCCAACCTGGACCTCTGACCAGTACCTGAGAGGCGGACTGTGTGCCTATGCTGGAGCTCCCGGCTACATCCGAAGCAGCCAGGACCTGAGTTGTGACTTCTGCAATGATGTCCTTGCACGAGCCAAATACTTCAGGAGACATGGCTTCTAA

>Panda_LygA2

ATGCAATCCTCTATCCTGTTTTGGGGACTTGTTGCACTCATTGGCACTTCAAGGGGCTCGTACCCTTTCACTCACTCAATGAGCCCTCACCTGCATCCCCGCCTGTACCATGGCTGCTATGGTGACATCATGACCATGGAGACCTCTGGTGCCGCCTGTGATATAACCAGGTTGATGAACTGTGGGATCCGTGGTTCTGAAATGTTTGCTGAGATGGATTTGAAGGCCTTAAAAACTTACCAGATTCTGATCAAAGAAGTTGGGCTGAGATATTGTGTGGACCCTGCTCTCATCGCAGCCATCATCTCCAGAGAAAGCCACGGTGGAAGTGTCCTGCAAGATGGCTGGGACCACAGGGGACTTAAATTTGGCTTGATGCAGCTGGATAAAAAAATTCATCACCCTATTGGTACGTGGGACAGCAAAGAACACCTTTTGCAGGCTGTTGGGATTCTAGCAGACAAAATTAAGGCAATCCAGAAAAAATTCCCCACGTGGAGTGTGGCTCAACACCTCAAAGGTGGTCTCTCGGCATTTAAGTCAGGAACCAATGCCATTGTCACCCCCACGGACATAGACAATGACTTGGTCAATGATCTTCTTGCCCGAGCTAAATTCTATAAAAGACATGGCTTCTAG

>Squirrel_LygA1

ATGTCTGCTTTGTGGCTGTTGCTGGGCCTCTTGGCCTTGACTGACTTATCTGAAAGCAGCAATTGGGGATGTTACGGAAACATCCGAACCCTGGACACCCCAGGAGCATCCTGTGGGGTTGGAAGACGTCACGGCCTGAACTACTGTGGAGTTCGTGCTTCCGAAAGGCTGGCTGAAATAGACATGCCCTATCTCGTGAGATATCAACCCATGATGCGAACCGTTGGCCAGAGGTACTGTGTGGATCCTGCAGTGATCGCTGGTGTCTTGTCCAGGCAGTCCCAAGGGGGCAACTTTCTGGTCAACGTGGGCAACACAGGCAACGTGGGCAACATGGAAGATGGAGTCAGGGTGCTGCAGGACCCAAATCTTTATGCTCCCTCATCCTGGATCACTGAATCCCAGGTTTCCCAGATGACTGGGGTTCTGACTTCTAGAATCAAAGAAATCCAGAGGCGATTTCCTACCTGGACCCCCGACCAGTACCTGAGAGGTGGACTCTGTGCCTACAGTGGGGGCCCTGGCTTTGTCAGAAGCAGCCAGGACCTGGGCTGTGACTTCTGCAATGATGTCCTTGCACGAGCCAAGTACCTCAAGAGACATGGCTTCTAG

>Squirrel_LygA2

ATGCTACCCTCTGTTGTGTTTTTGGGACTTACAGTCCTCATTGGCACTTCCAGGGGTTCCTACCCGTTCACTCATTCCATGGACAGTCACCTGCATCCTCGCCTGTACCACGGCTGCTATGGGGACATTATGACCATGGAGGCTTCCGGGGCCCCCTGTGACATAGACAATTTGATTAACTGCGGGATCCGTGGCTCTGAAATGTTTGCTGAGATGGACTTGAAGGCCATAAAGCCTTATCAGACGCTGATCAAGGAAGTGGGGCAGAGGTACTGCATTGACCCTGCTATCATTGCAGCCATCATCTCCCGAGAAAGCCATGGTGGCACTGTTCTTCAAAAAGGCTGGGACCACCGGGGACTTAAATTTGGCCTGATGCAGCTCGATAAACAAACCCACCACCCTGTTGGTTCCTGGGACAGCAAAGAGCACCTTTTGCAAGGCGCTGGTATTCTAACAGATAGAATTAAAGCCATCCAGAGAAAATTCCCTAAGTGGAGTGCTGCTCAACACCTGCAAGGTGGTCTCTCTGCCTTTAAGTCAGGAATCGAAACCATTGTCACCCCTGCAGACATAGACACTGACCTCGTCAATGATCTTCTTGTCCGAGCTAAATTCTATAGAAGACATGGCTTCTAG

>Cat_LygA1

ATGTCTGTGCTGTGGCTGCTTCTGGGTCTCCTTGCCCTTACTGATTTATCTGAAAGCAGCAACTGGGGATGTTATGGAAACATCCGAAACTTCAACACCCCTGGAGCATCTTGTGGGATTGGAAAACGTCAAGGCCTGAACTACTGTGGAGTTCGTGCTTCTGAAAGGCTGGCTGAAATAGACATGCCATACATCCTGAGATATCAGCCTGTGATTCGTACTGTTGGCCAAAAGTACTGCATGGATCCTGCAGTGATCGCTGGTGTCTTGTCCAGGGAGTCTCATGGTGGCAACATTATGGTCAATGTGGGCAATGTGGGTGATGGAATCGGGGTTGTTCAGGACCCTGGCCTTTATTCTCCCACATCCTGGATCAGTGAGTCCCAGGTTTCTCAGATAATCGAGGTCCTGACACTTAGGATCAAAGAAATCCAGACCAGGTTTCCAACCTGGACCCCTGACCAGTACCTGAGAGGTGGACTCTGTGCCTATGCTGGAGGTCCTGGCTACATCAGAAGCAGCCAAGACCTGAACTGTGACTTCTGCAATGATGTCCTTGCACGAGCCAAATACTTCAAGAGACACGGCTTCTAA

>Cat_LygA2

ATGCTATCCTCTATCCTGTTTTGGGGACTTGTTGCTCTCATTGGCACTTCTAGGGGCTCATACCCTTTCACTCAGTCAATGAGCCCTCACCTGCATCCCCGCCTGTACCACGGCTGCTACGGTGATATCATGACCATGGAGACCTCTGGTGCTTCTTGTGATATAACCAGGTTGATTAACTGTGGGATCCGTGGTTCTGAAATGTTTGCTGAGATGGATTTGAGGGCCTTAAAGCCTTACCAGATTCTGATCAAAGAAGTTGGGCTGAGGCATTGTGTGGACCCTGCTCTCATTGCAGCCATCATCTCCAGAGAAAGCCATGGTGGAGCAGTCCTGCAAGATGGCTGGGACCACAGAGGACTTAGATTTGGCTTGATGCAGCTTGATAAAAAAATTCATCACCCTGTCGGTACCTGGGACAGCAAAGAACACCTATTGCAGGCTGTTGGGATTCTAACAGACAAAATAAAGGCAATCCAGAAAAAGTTCCCCACGTGGAGTGTGGCTCAACACCTCAAAGGTGGTCTCTCGGCCTTTAAGTCAGGAATCGATGCTATTGCAACCCCAGTGGACATAGACAATGACTTGGTCAATGATCTTCTCGCCCGAGCTAAATTCTATAAAAGACATGGCTTCTAG

>Star_nosed_Mole_LygA1

ATGTCTGTACTGTGGCTGTTACTGGGCCTCCTTGCCCTTACTGACTCATCTGAAAGCAGCAACTGGGGATGCTATGGGAACATCCGAACTCTCAGCACTCCTGGGGCATCTTGTGGGTTTGGACGCCGCCGAGGCCTCAGCTACTGTGGAGTCCGTGCTTCCGAAAGACTGGCTGAAATAGACATGCCATACTTACTGAGATATCAACCCATGATGCGTACTGTTGGTCAAAAGTACTGCATGGATCCTGCAGTGATCGCTGGTGTCCTGTCCAGGGAGTCTAACGGTGGCAACATTCTGGTCAATGTGGGCAATGTGGATGATGGAACCAGGATGGTGCAGGATTCTAGCTATTATGTTCCCACATCCAGGATAAGCCAATCTCAGCTTTCACAAATGACCGAAAACTTGACTTTTAGAATCAAAGAAGTCCAGAGGAGGTTTCCAACCTGGACCCCCGACCAATATCTGAGAGGTGGACTCTGTGCCTACAGTGGAGGAGATGGCTATGTCAGAAGTACCCAGGATCTGAGCTGTGACTTCTGCAATGATGTCCTTGCACGAGCTAAATACCTCAAGAGACATGGCTTCTAA

>Star_nosed_mole_LygA2

ATGATATCCTCTGTCCTATTTTTGGGACTTATTTCTCTCATTGGTACTTCTAAGGGCTTGTACTCTTTAACTCACTCTATGAATCCTCACCTGCACCCTCGCCTGTACCATGGCTGCTATGGTGACATCCTGACCATGGAAACCCCTGGGGCCTCCTGTGATATAACAAGGTTGATTAATTGTGGGATCCGTGGTTCTGAAATGTTTGCTGAGATGGATTTGAGGGCCATAAAGCCTTACCAAACTCTGATCAAAGAAGTTGGGCTGAGGTATTGTGTGGACCCAGCTCTCATTGCAGCCATCATCTCCAGAGAAAGCCATGGTGGAACTGTCCTGAAAGATGGCTGGGACCACAGGGGACATAAGTTTGGCTTGATGCAGCTTGATAAACAAATTTACAAACCTGTTGGTGCCTGGGACAGCAAAGAACACCTTTTGCAGGCTGTTGGGATTCTCACAGACAGAATTAAGATGATCCAGAAAAAATTCCCCACGTGGACTGTGGCTCAACACCTCAAAGGTGGTCTCTCGGCCTATAAGTCAGGAGCTGATGCAATTGTCACCCCCATGGACATAGACAATGACTTGGTCAATGATCTTCTTGCCAGAGCTAAATTCTATAAAAGACATAGCTTCTAG

>Dog_LygA1

ATGTCTGTGCTGTGGCTGCTTCTGGGGCTTCTGGCCCTTACTGATTCATCTGAAAGCAGCAACTGGGGATGCTATGGAAACATCCGAAACGTTGAGACCCCTGGGGCGTCCTGTGGGATTGGCAAGCGTCATGGCCTGAACTACTGTGGAGTTCGTGCTTCTGAAAGGCTGGCTGAAATAGACATGCCCTACCTCCTGAGATACCAGCCGGTGATTCACACTGTTGGCCAGAAGTACTGTGTGGATCCTGCAGTGATCGCTGGTGTCTTGTCCAGGGAGTCTCATGGCAGCAACGCTATGGTCAATGTGGGCAACACGGGCAACGGCATCGGGGACCCTGGTTTTTATGCTCCCACATCCTGGATCAGCGAGTCTCAAGTTTCTCAGATAACTGAGGTCCTTACTGTTAGGATCAAAGAAATTCAAAGGAGGTTTCCAACTTGGACCTCTGACCAGCACCTGAAAGGTGGACTCTGTGCCTATGCTGGAGGTCCTGGCTACATCAGAAGCAGCCAGGACCTGAGCTGTGACTTCTGCAATGACGTCCTTGCACGAGCCAAATACTTCAAGAGACATGGCTTCTAA

>Dog_LygA2

ATGCTATCCTCTATCCTGCTTTGGGGACTTATTGCCCTCATTGGCACTTCTAGGGGCTCGTACCCTTTCACTCACTCAATGAAGCCCCACCTGCATCCCCGCCTATACCATGGCTGCTATGGTGACATCATGACCATGGAGACCTCTGGTGCCGCCTGTGATATAACCAGGTTGATGAACTGTGGGATCCGTGGTTCTGAAATGTTTGCTGAGATGGATTTGAGAGCCTTAAAGACTTACCAGATTCTGATCAAAGAAGTTGGGCTGAGGCATTGTGTAGACCCTGCACTCATTGCTGCCATCATCTCCAGAGAAAGCCATGGTGGAGCCGTCCTGAAAGATGGTTGGGACCACAGGGGACTTAAATTTGGCTTGATGCAGCTTGATAAAAAAATTCATCACCCTGTTGGTACCTGGGACAGCAAAGAACACCTTCTGCAGGCTGTTGGGATTCTCACAGACAAAATTAAGGCAATCCAGAAAAAATTCCCCACGTGGAATGTGGCTCAACACCTCAAAGGTGGTCTCTCGGCCTATAAGTCAGGAATCGATGCCATTGTCACCCCAGTGGATATAGACAATGACTTGGTCAATGATCTTCTCGCTCGAGCTAAATTCTATAAAAGACATGGCTTCTAG

>European_shrew_LygA1

ATGCCTGCACTGTGGCTGCTGCTGGGCCTGCTGGCCCTGGCTGGTTCATCAGAAAGTAGCAATTGGGGCTGTTATGGAAACATCCGAACCCTGAGCACTCCTGGGGCATCATGCGGGATTGGACGGCGCCAAGGCCTGAATTACTGTGGAGTTCGTGCCTCTGAGAGGTTGGCTGAGATGGACATGCCGTACCTGGTGAGGTACCAGCCCATGATGCGCACTGTGGGCCAGAAGTACTGCATGGACCCTGCAGTGATTGCTGGTGTCCTGTCCAGGGAGTCTCTGGGTGGCAAGGTGCTGGTCAACGTGGGCAGCGCGGATGATGGAGCCAGGCTGGTACAGGACTCTGGCATTTATGCCCCCACAGTGCTCATCAGTCAGGGGCAGGTTTCCCAAATGACTGAAGACCTCACTCTTAGAATCAAGGAAATCCAGAGACGGTTTCCAACCTGGACCCCTGATCAGTATCTGAGAGGTGGGCTCTGTGCCTACAGTGGAGGTCCTGGCTATGTCAGAGGCACCCAGGACCTGAGCTGTGACTTCTGCAACGATGTCCTCGCACGAGCAAAGTACCTGAAGAGACACGGCTTCTGA

>European_shrew_LygA2

ATGTTATCCTCTATCCTATTTTGGGGACTCGCGGCTCTCATGGGCGCAGCCGAGGGCCTGTACTCCCTGACCCACTCCATGAACCCTCACCTGCACCCGCGCCTGTACCACGGCTGCTATGGGGACATCCTGCACATGGAGACAGCTGGTGCGCCCTGCGACATCTCCCGGCTCTTCAACTGCGGGATCCGTGGTTCGGAGATGTTTGCGGAGATGGATCTGAGAGCGATAAAGCCTTACCAGAATCTGATCAAAGAGGTTGGGCTGAGGTACTGCGTGGACCCTGCTCTGGTGGCTGCCATCATCTCCAGGGAGAGCCATGGTGGAACCGTTCTGCAAGATGGCTGGGACCACACGGGAGCCAAATTCGGCCTGATGCAGCTGGATAAGCAAGTGTATAAACCTGTTGGTGCCTGGGACAGCAAAGAACACCTTCTACAGGGTGTTGGGATTCTAACAAACAATATTAAGGCGATCCAGAAAAAATTTCCCACCTGGAATGTAACTCAGCACCTCAAAGGCGGCCTCTCAGCCTTTAAGTCAGGAGCCGACGCCATTGACTCACCCATGGACATAGACAATGACTTAACCAATGATCTTCTTGCCCGAGCTAAATTCTATAAAAGACACGGCTTTTAG

>Walrus_LygA1

ATGTCTGTGCTATGGCTGCTTCTGGGTCTTCTTGCCCTTACTGATTCCTCTGCAAGCAGCAACTGGGGATGCTATGGAAACATCCGAAACTTTGAGACCCCTGGGGCTTCTTGCGGGGTTGGAAAGCGTCATGGCCTGAACTACTGTGGAGTTCGTGCTTCGGAGAGGCTGGCTGAAATAGACATGCCATACCTCCTGAGATACCAGCCCGTGATTCGTACTGTTGGCCAAAAATACTGTGTGGATCCTGCAGTGATCGCTGGAGTCTTGTCCAGGGGGTCTCATGGCGGCAACGTTATGGTCAATGTGGGCAACATGGGCAATGGCATCGGGGTTGTGCAGGACCCTGGTCTTTATGCTCCCACATCCTGGATCAGCGAGTCCCAGGTTTCTCAGATAATCGAGGTCCTGACTGTTAGGATCAAAGAAATCCAGAGGAGGTTTCCAACCTGGACCTCTGACCAGTACCTGAGAGGTGGACTCTGTGCTTATGCTGGAGCTCCCAGCTACATCAGAAGCAGCCAGGACCTGAGGTGTGACTTCTGCAATGATGTCCTTGCACGAGCCAAATACTTCAAGAGACATGGCTTCTAA

>Walrus_LygA2

ATGCTATCCTCTATCCTGTTTTGGGGACTTATTGCCCTCATTGGCACTTCTAGGGGCTCGTACCCTTTCACTCACTCAATGAACCCTCACCTGCATCCCCGCCTGTACCATGGCTGCTACGGTGACATCATGACCATGGAGACCTCTGGTGCCGCCTGTGATATAACCAGGTTGATGAACTGCGGGATCCGTGGTTCTGAAATGTTTGCTGAGATGGATTTGAGGGCCTTAAAAACTTACCAGACTCTGATCAAAGAAGTTGGGCTGAGATATTGTGTGGACCCTGCTCTCATCGCAGCCATCATCTCGAGAGAAAGCCATGGTGGAGCTGTCCTGCAAGATGGCTGGGACCACAGGGGACTTAAATTTGGCTTGATGCAGCTTGATAAAAAAATTCATCACCCTGTTGGTACCTGGGACAGCAAAGAACACCTTTTGCAGGCTGTTGGGATTCTAACAGACAAAATTAAGGCAATCCAGAAAAAATTCCCCATGTGGAGTGTGGCTCAACACCTCAAAGGTGGTCTCTCAGCCTTTAAGTCAGGAATCGATGCCATTGTCACCCCAACGGACATAGACAAAGACTTGGTCAATGATCTTCTCGCCCGAGCTAAATTCTATAGAAGACATGGCTTCTAG

>Armadillo_LygA1

ATGTCTGCCTTGTGGCTGCTTCTGGGCCTCCTGGCCTTTCCTTCCTCGGCGGAAAGCAGTGACTGGGGATGCTACGGAAACATCCGGACCTTCGACACCCCTGGGGCGTCGTGTGGAATTGGAAGGCGACAAGGGCTGAACTACTGTGGAGTCCGGGCTTCTGAAAGGCTGGCGGAAATTGACATGCCGTACCTGCTGAGGTACCAACCCGTGATGCGCACCGTGGGCCAGAAGTACTGTGTGGACCCGGCAGTCATCGCTGGAGTCCTGTCCCGGCAGTTGCACGGTGGCAAGGTTCTGGTCAACGTGGGGAGTGTGGGCGACAGGGTCGGCATGGTACAGGAACCCAACCTTTATGCTCCCAGCTCCTGGATCAGTGAATCCCAGGTTGCCGAGGTGACCAGAGTTCTCGTTGTTAGAATCAAAGAAATCCAAAGGAGGTTTTCAACCTGGACCCCAGACCAGTACTTGAGAGGTGGACTCTGCGCCTACGGTGGAGGTGTTGGCTATGTCAGGAGCACCCAGGACCTGAGCTGCGACTTTTGTGATGACGTCCTTGCACGAGCCAAATTCTACAAGAGACATGGCTTCTGA

>Armadillo_LygA2

ATGGGCACCAAGCTACCTCCTGTCATAGTTTTGGGACTTCTGGCCCTCGTTGGCTCTTCTAGGGGCTCGCACCCCTTCATTCACCTGGTGACCCCTCCCCTCCATCCCCGCCTGTACCATGGGTGCTACGGGGACATCATGACGATGGAAACGCCTGGCGCGTCCTGTGATAGAAGCATGCTGATTAACTGCGGGATCCGGGGTTCTGAAATGTTTGCTGAGATGGATTTGAGAGCCATAAAGCCTTACCAGACCCTGATCAAAGAAGTGGGACAGAGGCATTGTGTGGACCCCGCGCTCATCGCAGCCATCATCTCCAGAGAAAGCCACGGCGGAACCATCCTGCTTGATGGCTGGAACCACACAGGACTTAAATTTGGCTTGATGCAGCTCGATAAAAAAATGTTTCATCCTGTTGGTGCCTGGGACAGCAAAGAACACCTGTTGCAGGCTGTTGGGATTCTAACAGATAGAATTAAGGCAATGCAGAAAAAGTTCCCCACATGGAGTGCGGTTCAACATCTCAAAGGTGGTCTCTCGGCCTTTAAGTCAGGAGTCGAAGCCATTGTCACCCCTTTGGATGTAGACACTGACTTTTTCAATGATCTTGTTGCACGTGCTAAATTCTATAAAAGACACGGCTTCTAG

>Chinchilla_LygA1

ATGTCTGTGTTGTGGCTGCTTCTGGGCCTCCTGGCCCTGGCTGACTCATCTGAAAGCAGCAACTGGGGATGCTACGGGAACATCCAAACCCTGGACACCCCAGGAGCATCCTGCGGGATTGGAAGACGCCACGGCCTGACCTACTGTGGAGTCCGTGCTTCTGAAAGACTGGCTGAAATAGACCTGCCTTACCTGCTGCGATACCAGCCCATCGTGCGGACGGTCGGCCGGAAGTACTGCGTGGATCCTGCAGTGATCGCTGGGGTTCTGTCCAGGGAGTCTCTCCGGGGCAGTATTCCGATCAACGTGGGTGTTCCGATCAACATGGCGCAGGACACAAACGCTTTTGCTCCCACATCCTGGATCAGCGAGTCCCAGGTTTCCCAGATGACCAAGGTTCTGACTAGTCGAATCAAAGAAATCCAGAGGACATTTCCAACCTGGACCCCCACCCAGTACCTGAGAGGAGGACTCTGTGCCTATGGCGGGGGCGCTGGCTTTGTCAGAAGCAACGAGGACTTGAGCTGTGACTTCTGCAATGATGTTCTTGCGCGTGCCAAATACTTCAAGAGACACGGCTTCTAG

>Chinchilla_LygA2

ATGTTGCCCTCTATCGTGCTCGTGGGACTTACTGCTCTCATTGGCACGAGCAGGGGCTCCTACCCTTTCCCTCATGCGCTGCGGCCCCACCTGCACCCCCGCCTGTACCACGGCTGCTACGGGGACATCACCACCATGGAGACCTCGGGGGTGCGCTGCGACATCACCAACCTGCTCAGCTGCGGGATCCGTGGTTCTGAAATGTTTGCCGACATGGATCTGAACTCCATAAGACCCTACCAGACTCTGATCAAAGAAGTGAGCCAGAGGTACTGCGTGGACCCTGCGCTCATCGCGGCCATCATCTCCAGAGAGAGCCACGGCGGCACCGTCCTGCAGCGTGGCTGGGACCACAAGGGCCTGAAGTTCGGCCTGATGCAGCTGGATAAAAGTATCCACCCGACCGGCGCCTGGGACAGCAAAGAACACCTTTCGCAGGGTGTGGGGATTCTAGCAGAAAACATTAAGGTGATCCAGAGAAAATTCCCCATGTGGAGTGTGGATCAGCACCTCAAAGGTGGTCTCTCTGCCTTTAAGTCAGGAGCCGAAGCTATTGCTGCCCCGGAAGATATTGACTCTAACTACGTAGATGACGTGATTGCTCGAGCCCGATTCTATAAAACCCATGGCTTCTAG

>Little_brown_bat_LygA1

ATGTCTGTGCTGTGGCTGCTTCTGGGCCTCCTTGCCCTCACCGACTCATCTGAAAGTGGCAACTGGGGATGCTACGGAAACATCCGAACCCTCGACACGCCCGGGGCATCTTGTGGGATTGGAAGACGCCGAGGCCTGAACTACTGTGGAGTCCGTGCTTCTGAGAGGCTGGCTGAGATCGATATGCCGTACCTCCTGAGATATCAACCCATGATGCGCACCGTTGGCCAAAAGTACTGTGTGGATCCTGCAGTGATCGCTGGTGTCGTGTCCAGGGGGTATCCTGGCGGCAACCTTCTGGTCCAGGCTGGCAATGTGGATGATGGAGTCAGGGTGGTGCAGGACACTGGCCACTATGCCCCTACTACCTGGATCAGCGAGTCGCAGCTGTCCCAGATGACTGAGGTCCTGACTGTTAGAATCAAGGAAATCCAGAGGAGGTTCCCGACCTGGAGCCCGGACCAGTGCCTGAGAGGGGGGCTCTGTGCCTACGTCGGAGGGCCCAGCTATGTCAGAAGCAGCCAGGACCTGGGCTGTGACTTCTGCAATGATGTCCTTGCACGAGCCAAATACTTCAAGAGACATGGCTTCTAA

>Brant’s_Bat_LygA1

ATGTCTGTGCTGTGGCTGCTTCTGGGCCTCCTTGCCCTCACCGACTCATCTGAAAGTGGCAACTGGGGATGCTACGGAAACATCCGAACCCTCGACACGCCCGGGGCATCTTGTGGGATTGGAAGACGCCGAGGCCTGAACTACTGTGGAGTCCGTGCTTCTGAGAGGCTGGCTGAGATCGATATGCCGTACCTCCTGAGATATCAACCCATGATGCGCACCGTTGGCCAAAAGTACTGTGTGGATCCTGCAGTGATCGCTGGTGTCCTGTCCAGGGGGTATCCTGGCGGCAACCTTCTGGTCCAGGCTGGCAATGTGGATGATGGAGTCAGGGTGGTGCAGGACACTGGCCACTATGCCCCTACTACCTGGATCAGCGAGTCGCAGCTGTCCCAGATGACTGAGGTCCTGACTGTTAGAATCAAAGACATCCAGAGGAGGTTCCCGACCTGGAGCCCCGACCAGTGCCTGAGAGGGGGGCTCTGTGCCTACGTCGGAGGGCCCAGCTATGTCAGAAGCAGCCAGGACCTGGGCTGTGACTTCTGCAATGATGTCCTTGCACGAGCCAAATACTTCAAGAGACACGGCTTCTAA

>Brant’s_Bat_LygA2

ATGTTATCCTCTGTCCTGTTCGGGGGACTCGTTGCCCTCATTGGCACTTCCAGGGGCTCTTATCCTTTTAGTCACTCAGCGAGCCCTTACCTGCACCCCCGCCTGTACCGCGGCTGCTATGGCGACATCATGACCATGGAGACTCCTGGGACTGCCTGTGATATAGCCAGGTTGATTAACTGCGGGATTCGTGGTTCTGAAATGTTTGCTGAAATGGATTTGAGGGCCTTAAAGCCGTACCAGACTCTGATCAAAGAAGTTGGGCGGAGGCATTGCGTGGACCCTGCCCTCATTGCAGCCATCATCTCCAGAGAAAGCCACGGTGGAGCTGTCCTGCAGGATGGCTGGGACCACAGGGGCCTGAAATTTGGCCTGATGCAGCTTGATAAAAAAATGTATTACCCTGTTGGCACCTGGGACAGCAAAGAGCACCTTCTGCAGGCTGTTGGGATTCTGACAGACAGAATTAAGGCAATCCAGAGAAAATTCCCCATGTGGAGTGTGGACCAACACCTCAAAGGTGGTATCTTGGCCTTTAAGTCAGGAGTCGAAGCCATTGCCACCCCCTTGGACATAGAAGCTGACTTGGTCAATGACATTCTCGCCCGAGCTCAGTTCTATAGGAGACACGGCTTCTAG

>Vampire_bat_LygA1

ATGTCTGTGCTGTGGCTGCTTCTGGGTCTCCTTGCCCTTATTGACTCATCTGAAAGCGGCAACTGGGGATGCTATGGAAACATCCGGACCCTGGACACCCCCGGGGCAGCTTGTGGAGTTGGAAGGCGCAGAGGCCTGAACTACTGTGGAGTTCGTGCTTCTGAAAGGATGGCTGAAATCGACCTGCCGTACCTACTGAGATATCAGCCTGTGATGCATACCGTTGGCCAAAAGTACTGTGTGGATCCTGCCGTGATCGCTGGTGTCATGTCCAGGGAGTCTCCTAGTGGCAGTGTCCTGGTCAATGTGGGCACTGTGGACAACGGAATTGGGGTGGTGCAGGACCCTGGCCATTACGTGCCCACAACATGGATCAGTGAGTCCCAGCTTTCTCGGATGACTGAGGTCCTGACTGTTAGAATCAAAGAAATCCAGAGGAGGTTTCCAACCTGGACCCCCGACCAGTACCTGAGAGGTGGACTCTGCGCCTACGTTGGAGGTCCTGGCTATGTCAGAAGCACCCAGGACCTGAGCTGTGACTTCTGCAATGATGTCCTTGCACGAGCCAAATACTTCAAGAGACACGGCTTCTAA

>Vampire_bat_LygA2

ACTATCTTGTCCTCTTTCCCTTGCTCAATGAACCCCCACCTGTATCCCTGCATGTACCATGGCTGCTATGATGACATCATGAATATGGAGACCTCTAGTGCCACCTGTGATATAACCAGGTTGATTAACTACGGGATCCAAGGTTCTGAAATATTTACTGAGATAGACTTGAGGGCATTGAAACCTTACCAGGCTCTGATCAAAGAAGTTGGGCTGAGGCACGTGGACCCAGTCCTTATTGCAACTCTCATCTCCAGAGAAAGCCATGGTGGAGCTGTCGTGCAAGATGGCGACCACCAGAGCCTTAAATTTGGCCTGATACAGCTAGCTAAAAATATTCATCACCCTGTTGGAACCGACAGCAAAGAGCACCTTCTGCAGGCTGTTGGGATTGTAACAGACAGAGTTAAGGCAATCCAGAAAAAATTCTCCATGTGGAGTGTGGCTCAACACCTCAAAGGTGGCCTCTCGGCCTTTAAGTCAGGAATTGAAGCCATTGTCACTCCTGCGGACACAGAGACTTACTTGGTCAATGACCTTCTCACCGCTAAATTTTATAAAAGACATGGTTTCTAG

>Pika_LygA1

ATGTCTGTTCTGTGGCTACTCCTGGGTCTCCTCGCACTGACTGCCTCATCTGAAAGCAGCAACTGGGGATGCTATGGCAACATTCGAACCCTGGACACCCCCGGAGCGTCTTGTGGGATTGGAAGACGTTATGGTCTGCCCTACTGTGGAGTCCGTGCTTCTGAAAGGCTGGCAGAAATAGACATGCCACGCCTCCTGAGATATCAACCAATGATGCGAACTGTGGGCCAAAGATACTGTGTGGATCCTGCAGTCATTGCTGGTGTCTTGTCCCGGGAGTCTCAAGGTAGTAACGTTCTGGTCCATGTGGGCTCCGCAGATGATGGAATTAGAGCGGCGCAGGTAACTGGTCCTGCCCTTTATGCTCCCACATCTTGGATCAGTGAGCCCCAGGTTTCCCAGCTGACTGAGGTCCTGACGGCCAGAATCAAAGAAATCCAGAGGAGATTTCCCACCTGGACTCCTGACCAGCACCTGAGAGGTGGACTCTGTGCCTACAACGGTGGTCCTGCCTATGTCCGAAGCACCCAGGACCTGAGCTGTGAATTCTGCAACGATGTCCTTGCTCGAGCCAAATACCTCAAGAGACATGGCTTCTAG

>Pika_LygA2

ATGCTGTCCCCTGTCATCTTTGTGGGACTTATTGCCCTCATTGGCACTTCCAGGGGCTCGTACCCCTACATACACTCAATAAACCCTCACCTGCATCCTCGCCTGTACCATGGCTGCTACGGGGACATCTTGACCATGGAGACCTCGGGGGCACTCTGTGATATAAATACTTTGCTAAACTGCGGGATTCGTGGTTCAGAGATGTTTGCTGAGATGGACTTGAAGACCATAAGGCCTTACCAGAATCTGATCAAGGAAGTAGGACAGAGACATTGTGTAGACCCTGCTCTCATTGCAGCCATCATCTCCAGAGAAAGCCACGGAGGAGCCGTTCTGCAAAATGGATGGGACTACAGAGGCCTTAAATTTGGCTTGATGCAGCTTGATAAACACATTTACCACCCTGTTGGTTCCTGGGACAGCAAAGAACACCTCTTACAGGGTGTTGGGATATTAACAGACAGAATTAAGGCAATCCAGAAAAAATTTCCTGCCTGGAGCGTGACTCAACACCTCAAAGGTGGTCTCTTGGCCTTTAAGTCAGGAATTGAAGGTGTCATCTCACCTGCAGACATAGAAGCTGACTTCGTCAATGATGTCATTGCCCGAGCTAAATTCTATAAAAAACATGGTTACTAG

>Galago_LygA1

ATGTCTGCATTGTGGCTGCTTCTGGGCCTCCTTGCCCTGACTGACTCGTCTGAAAGCAGCAACTGGGGATGCTATGGAAACATCCGAACCCTGGTCACCCCGGGGGCATCTTGTGGGATTGGAAGACGCCACGGCCTGAACTACTGTGGAGTTCGTGCTTCCGAAAGGCTGGCTGAAATAGACATGCCGTACCTACTGAAATATCAACCGATGATGCAAACTGTTGGCCAAAAGTACTGCATGGATCCTGCAGTGATCGCTGGTCTTTTGTCTAGGGAGTCTCAGGGTGGCAACATGCTGGTCAACGTGGGCAACGTAGGCAATGTGGGCAATGGGATTAAGGTGGTGCAGGATGCTGACTTTTCCACACCCATGTCCTGGATTAGTGAGTCCCGGGTTTCTCAGATAACTGAGACTCTTACCATTAGAATCAAAGAAATCCAGAGGAGGTTTCCAGCCTGGACCCCTGACCAGCACCTGAGGGGAGGACTCTGTGCCTACAGTGGGGGTGCTGGCTATGTCAGAAGCAGCGAGGACCTGAGCTGTGACTTCTGCAATGATGTCCTTGCCAGAGCCAAGTACCTCAAGAGACACGGCTTCTAA

>Ferret_LygA1

ATGTCTGTGCTGTGGCTACTTCTGGGTCTTCTTGCCCTTACTGATTTATCTGAAAGCAGCAACTGGGGATGCTATGGAAATATCCGAAACTTTGAGACCCCTGGGGCTTCTTGCGGGATTGGAAAGCGTCATGGCCTGAACTACTGTGGAGTTCGTGCTTCTGAAAGGCTGGCTGAAATAGACATGCCATACCTCTTGAGATACCAGCCTGTGATTCGTACTGCTGGCCAAAAGTACTGTGTGGATCCTGCAGTGATTGCTGGTGTCTTGTCCAGGGAGTCTCATGGCGGCAACGTTATGACCAATGTGGGCAACATGGGCGATGGCATCGGGGCTGTGCAGGACCTTGGTCTTTATGCTCCAACATCCTGGATCAGCGAGTCCCGGGTTTCTCAAATAACCGAGGTCTTAACTGTTAGGATCAAAGAAATCCAGAGGAGGTTTCCAACCTGGAACTCTGATCAGTATCTAAGAGGTGGACTCTGTGCTTATGCTGGAGCTCCTGAGTACATCAGAAGCAGCCAGGACCTGAGCTGTGATTTCTGCAATGATGTCCTTGCCCGAGCCAAATACTTCAAGAGACATGGCTTCTAA

>Ferret_LygA2

ATGCTGTCCTTTATCCTGTATTGGGGACTTATTGCCCTCATTGGCACTTCTAGGGGCTCGTACCCCTTCACTCACTCAATGAACCCTCACCTGCATCCCCGCTTGTACCATGGCTGCTATGGTGATATCATGACCATGGAGACCTCTGGTGCCACCTGTGATATAACCAGGTTGATGAACTGTGGGATCCGTGGTTCTGAAATGTTTGCTGAGATGGATTTGAGGGCCTTAAAACCTTACCAGATTCTGATCAAAGAAGTTGGGCTGAGGTACTGTGTGGACCCTGCTCTCATCGCAGCCATAATCTCCAGAGAAAGCCATGGTGGAGCTGTCCTACAAGATGGCTGGGACCACAGGGGACTTAAATTTGGCTTGATGCAGCTTGATAAAAAAATTCATCACCCTGTTGGTACCTGGGACAGCAAAGAACACCTTTTGCAGGGTGTTGGAATTCTAGCAGACAAAATTAAGGCAATCCAGAAAAAATTCCCCACGTGGAGTGTGGCTCAACACCTGAAAGGTGGTCTCTCAGCCTTTAAGTCAGGAATTGATTCCATTGTCACCCCAACGGACATAGACAATGACTTGGTCAATGATCTTCTTGCCCGAGCTAAATTCTATAAAAGACATGGCTTCTAA

>Mole_rat_LygA1

ATGTCTGCATTGTGGCTGCTTCTGGGCCTCCTGGCCTTGACTGGCTCATCTGAAAGCAGCAATTGGGGATGCTATGGGAACATCCGCACCCTGGACAGCCCGGGAGCATCCTGTGGGATTGGAAGACGTTATGGCCTGACCTACTGTGGAGTCCGTGCTTCTGAAAGGCTGGCTGAAATAGACCTGCCTTACCTGCTAAGATACCAGCCCATTGTTCGGACAGTTGGCCGGAAGTACTGCATGGATCCTGCAGTGATCGCTGGGGTTCTGTCCAGGGAGTCTCTCCGGAGCAGGGTTCCAGTCAACGTGGCTGAGCAATACTCCATGGTGTATGACATAAATGTTTTTGCTCCCAAATCTTGGATCAGCGAGTCCCAGGTTTCCCAGATGACTAAGGTTCTGACTACTAGAATCAAAGAAATCCAGAGGACATTTCCAACCTGGACCCCCGACCAGTACCTGAGAGCTGGGCTGTGCTCCTACAGCGAGGCTGCTGGCTTTGTCCGAAGGGGCCAGGACCTGAGCTGCGACTTCTGCAATGATGTCCTTGCACGCGCCAAATACTTCAAGAGACATGGATTCTAG

>Mole_rat_LygA2

ATGCTGCCCTCTATCCTGTTTGTGGGACTTACTGCTCTCATCGGCATGAGCTGGGGCTCCTACCCTTTCCCTCACTCCCTTCGGCCTCACCTGCACCCCCGCCTATACCACGGCTGCTATGGGGACATCATGACCATGGAGACCTCCGGGGTGCGCTGCGACATCTCCAATCTGCTCAGCTGTGGGATCCGTGGTTCTGAAATGTTTGCCGATATGGATCTGAATGCCATAAGGACCTACCAGACTCTGATCAAAGAAGTGGGGCAGAGGTACTGCGTGGACCCTGCGCTCATCGCGGCTGTCATCTCCAGAGAGAGCCACGGGGGTGTCGTGCTGCAGCGTGGCTGGGACCACAGGGGACTGAAGTTTGGCCTGATGCAGCTTGATAAAAGTGTTCATCCTACTGGTGCCTGGGACAGCAAAGAACACCTTTCACAGGGTGTTGGGATTCTAGCGGAAAACATTAAGGTGATCCAGAGAAAATTCCCCATGTGGAGCGTGGATCAGCACCTCAAAGGTGGTCTCTCTGCCTTTAAGTCAGGAACAGAAGCCATTGCTGCCCCAGAAAATATCGACTCTGACTACGTGAATGATGTTATTGCCCGAGCTCGATTCTATAAAAGCCATGGCTTCTAG

>Kangaroo_rat_LygA1

ATGTCTGCACTGTGGCTCCTCCTTGGTCTTCTGGCCCTGACTTGCTCATCTGAAAGCAGCAACTGGGGGTGTTATGGAAACATCAGAACCCTGAATACACCTGGAGCATCTTGTGGGATTGGAAGACGTCATGGCCTGACTTATTGTGGAGTTCGAGCTTCTGAAAGGCTGGCTGAAATAGACATGCCATATCTACTGAGATATCAACCCATGATGCGAACCATTGGCCGGAGGTACTGCGTGGATCCTGCCGTGATCGCTGGGGTCTTGTCCAGGGAGTCTCAGGGTGGCAATATTCTGGTCAACGTGGGTGATGGAGACAGGATGGTGCAGGACTCAAGCTTTTACCCCTCCCCATCTTGGATCAACGAGTCCCAGATTTCCCAGGCAACTCAGCTTCTGACTGCTAGAATCAAAGACATTCAGACCAGGTTTCCAACATGGACCCCTGGCCAGTACCTGAGAGGTGGACTCTGTTCCTACAGTGGGGGTCCTGGCTTTGTCAAAAGCAGTCACGACCTGAGTTGTGACTTCTGCAATGATGTCCTGGCAAGAGCCAAATACTTCAAGCAACATGGCTTCTAG

>Kangaroo_rat_LygA2

ACTTCCAGGGCCTCATATTCTTTCACTCATGCCATGAATCCTCGCCTGCATCCTCGCCTGTACCATGGTTGCTACGGAGACATCATGACCATGGAGACCTCTGGGGCCCCCTGTGAGATAAACAATTTGATTAGTTGTGGGATCCGTGGTTCTGAAATGTTTGCTGAGATGGATCTAAAAGCCCTAAAGTCTTACCAGATTGTGATCAAAGAAGTGGGACAAAGATACTGCATCGATCCAGCTGTCATCGCAGCCATCATATCCAGGGAAAGCCATGGTGGAACTGTCCTGCAAAATGGCTGGGACCACAGGGGACTTAAATATGGCTTGATGCAGCTTGATAAACAACTTTACCATCCAGTTGGTGCCTGGGACAGCAAAGAACACCTTTTACAGGGCGTTGGGATTCTAGCAGACAAGATAAAGGCGATCCAGAGAAAATTCCCCACGTGGAGCGTGCCTCAACACTTAAAAGGTGGTCTCTCTGCCTTCAAGTCTGGAATTGACACCATTGATACACCCATGGACATAGATGCTGACTTAGTTAGTGACATTATAGCCAGAGCTAAATTCTTCAAAAAGCATGGTTTCTAG

>Jerboa_LygA1

ATGTCTACACTATGGATGCTTCTGGGCCTCCTGGCCCTGACTGGTTCATCTGAAAGCAGCAGTTGGGGATGCTATGGAAATATCCGGACCCTGAACACCCCTGGAGCATCTTGTGGGATTGGAAGACGTCATGGCTTGTCCTACTGTGGAGTTCGTGCTTCTGAAAGGCTGGCTGAAATAGACATGCCTCATCTTCTGAGATATCAACCCGTGATGCGAACTGTTGGCTGGAAATACTGTGTGGATCCTGCAGTGATCGCTGGTGTCTTGTCCAGAGAGTCTCAAGGAGGCAATGTTCTGGTCAACACAGGCCACGTGGGAGATGGAATCAGGGTGGTTCAGGACCGAAATTTTTATGCTCCTACATCCTGGATGAGTGAGTCCCAGGTCTCCCAGAAAATTGAAGTTCTAACTTCTAGAATCAAAGAAATTCAGACCAGGTTTCCAGCATGGGCCCCTGACCAGTACCTGAGAGGTGGACTTTGTGCCTACAGCAAGGGTACAGGCTTTGTCAGAAGCAGTCAGGATCTGAACTGTGACTTCTGCAATGATGTCCTGGCACGAGCAAAGTACTTCAAGAACCATGGCTTCTAG

>Jerboa_LygA2

ATGCTACCCTCATTCCTGTTTTGGGGACTTATTGCTGTCATTGGCACTTCCAGGGGCTCCTACCCGTTCTCTCACTCCATGAAGCCTCACCTGCATCCTCGCTTGTACCACGGCTGCTATGGGGACATCATGACCATGGAGACCTCTGGGGCCCCCTGTGACATAAACCATCTCATGAGCTGTGGGATCAGTGGTTCCGAAATGTTTGCTGAGATGGATTTGCAGACCCTAAGACCTTACCGGACCATGATCAAGGAAGTGGCACAGAGATACTGCATTGACCCTGCTGTCGTCGCCGCCATCATCTCCAGAGAGAGCCATGCGGGGGCTGTTCTGCACAATGGCTGGGACCACAGGGGACTTAAATTTGGCTTGATGCAGCTTGATAAACAAATTTACCACCCTATTGGTTCCTGGGACAGCAAAGAGCACCTTCTGCAGGGTACTGGAATTCTAGCAGAGAGAATTAAGGCAGTCCAGAAAAAATTCCCCACATGGAGTGTGGCTCAACACCTCAAAGGTGGTCTGACTGCCTTTCAGTCAGGAATGGACACCATTGTCACTCCTGTGGACATAGAAACTGACTTCGTCAATGATATTATTGCCCGAGCTAAATTCTATAAAAGACATGGTTTCTAA

>Degu_LygA1

ATGTCTGCATTGTGGCTGCTCCTGGGCCTGCTGGCCCTGACTGACTCATCTGAAAGCCGCGACGGGGGATGTTACGGGAACATCCAGACCCTGGACACCCCAGGAGCATCCTGTGGAATTGGAAGACGTCACGGCCTGACCTACTGTGGAGTCCGTGCTTCTGAAAGGCTGGCTGAAATCGACCTGCCTCACCTGCTGAAATACCAGCCCATTATGCGGACAGTCGGCCAGAAGTACTGTGTGGATCCTGCAGTGATCGCGGGGGTCCTGTCCAGGGAGTCTCTCCGAGGCAGGGTTCCAATCAACATGGGCATGCCGATCAACATACCGCAGGATACAAATGCTTTTGCTCCCACATCCTGGATCAGTGAGTCCCAGGTTTCCCAGATGACCAACGTCCTGACCAGTACAATCAAAGAAATCCAGAGGACATTTCCAACCTGGACCCCTACCCAGTACCTGAGAGGTGGACTCTGCGCCTACGGCAGGGGTTCTGGCTTTATCAGAAGCAGCCAGGACTTGAGCTGCGACTTCTGCAACGATGTCCTTGCACGTGCCAAATACTTCAAGAGACACGGCTTCTAG

>Degu_LygA2

ATGTGGCCCTCCATCGTGCTTGTGGGACTGACTGTTGTCATTGGCACGAGCAGGGGCTCCTACCCTTTCCCTCATGCCCTGCGGCCCCACCTGCACCCCCGCCTGTACCACGGTTGCTACGGGGACATCACGACCATGGAGACCTCTGGGGTGCGCTGCGACATCACAAACCTGCTCAGCTGCGGGATCCGTGGTTCTGAAATGTTTGCCGACATGGACCTGAACTCTATCAGGCCCTACCAGACTCTGATCAAGGAAGTGGGCCAGAGGTACTGCGTGGATCCCGCGCTCATCGCAGCCGTCATCTCCAGAGAGAGCCATGGCGGCACCGTCCTGCAGCGTGGCTGGGACCACAGGGGACTGAAGTTCGGCCTGATGCAGCTGGATAAAAATATTCACCCAACCGGTGCCTGGGACAGCAAAGAACACCTTTCGCAGGGTGTGGGGATTCTAGCAGAAAACATTAAGGTGATCCAGAGAAAATTCCCCATGTGGAGTGTGGACCAGCACCTCAAAGGTGGTCTCTCTGCCTTCAAGTCAGGAGCTGAAGCCATTGCCGCCCTGGAAGACATTGACTCAGGCTATGTTGACGATGTGATTGCCCGCGCTCGATTCTATAAAACGCATGGCTTCTAG

>Rhinoceros_LygA1

ATGTTTGTGCTGTGGCTGCTTCTGGGCCTCCTTGCTCTTACTGACTCATCTGAAGGCAGCAGTTGGGGATGCTATGGAGACATCCAAACTCTCGACACCCCTGGGGCATCCTGCGGGATTGGAAGACTTCGAGGCCTGAGCTACTGTGGAGTTCGTGCTTCTGAAAGGCTGGCTGAAATAGACAAGCCATACCTACTGAGATATCAACACATTATGCGTACTGTTGGCCAAAAGTACTGTGTGGACCCTGCAGTGATTGCTGGTGTCTTGTCCAGGGAGTCTCATGGTGGCAACGTTCAGGTCAACGTGGACAATGCAGGCGATGGAATCAGGGTGGTGCAGGACCCTGGCCTTTATGCTCCCACATCCCAGATCAGCGAATTCCAGGTTTCCCGGATGATTGAGGTCCTCATTGTTAGAATCAAAGAAATCCAGAGGAGGTTTCCAACCTGGACCCCCGACCAGTACCTGAGAGGTGGACTCTGCGCCTACACTGGAGGTGCAGGCTTTGTCAGAAGCCGCCAGGACCTGAGCTGTGACTTCTGCAATGATGTCCTTGCACGAGCCAAATACTTCAAGAGACATGGCTTCTAA

>Rhinoceros_LygA2

ATGCTCTCCTCTATTCTGTTTTTGGGACTTATTGCCCTTATCGGCACTTCCAGGGGCTTGTACCCTTTCACTCAGTCAATGAACCCTCACCTGCATCCCCGCCTGTACCATGGCTGCTATGGCAACATCATGATCATGGAGACCTCCGGCGCTGCCTGTGATATAACCAAGTTGATTAACTGCGGGATCCGTGGTTCTGAAATGTTTGCTGAGATGGATTTGAGGGCCTTAAAGCCTTACCAGATTCTGATCAAAGAAGTTGGGCTGAGACACTGCATGGACCCTGCTCTCATTGCAGCCATCATCTCCAGAGAAAGCCATGGTGGAACCGTCCTGAAAGATGGCTGGGACCACAAGGGACTTAAATTTGGCTTGATGCAGCTTGATAAAAAAATTCATCACCCTGTTGGTACCTGGGACAGCAAAGAACACCTTTTGCAGGCTGTTGGGATTCTAACAGAGAGAATTAAGGCAATCCAGAAAAAATTCCCCACGTGGAGTGTGGCTGAACACCTCAGAGGTGGTCTCTCGGCCTTTAAGTCAGGAATTGATGCCATTGCCACCCCCGAGGACATAGACAATGACTTGGTCAGTGATATTATTGCCCGAGCTAAATACTATAAAAGACATGGCTACTAG

>Hedgehog_LygA1

ATGTCTGCTTTGTGGCTGCTTCTGGGCCTCCTTGTCCTGACTGATCTATCTGAAAGCAGCAATTGGGGATGCTACGGAAACATCCGAACCCTCGAAACCCCTGGGGCATCTTGTGGGGTTGGAAGACAACGAGGCATCAACTACTGTGGGGTCCGAGCTTCTGAAAGGCTGGCTGAAATGGACATGCCCCTGCTGGTGAGATATCAACCCATCATGCGGACGGTCGGCCGGAAATACTGCGTGGACCCTGCCGTCATCGCCGCTATCTTGTCCAGGCAATCACATGGGGGCAACATTCTGCTCAACGTGGGGAACACGAACGATGGAATCGGGGTGGTGCAGGACTCGGGCCAATACGCTCCCACCTCCTGGATCAGCGAGTCCCAGCTTGCTGAAATGACGGAGGCTCTGACTGTTAGGATCAAAGGGATCCAGAGGAAGTTTCCAACCTGGACCCCAGACCAGTATCTGAGAGGTGGACTGTGTTCCTACAATGGAGGTGTTGGCTACGTCAGAAATAGCCAGGACCTGGGCTGTGACTTCTGCAACGATGTCCTTGCACGAGCCAAATACTATAAGAGACATGGCTTCTAA

>Horse_LygA1

ATGTCTGTGCTGTGGCTGCTTCTGGGCCTCCTTGCCCTTCCTGACTCCTCTGACGGTAGCAGTTGGGGATGCTACGGAGACATCCAAACCTTTGACACCCCTGGGGCATCTTGTGGGATTGGAAGACGTCGAGGCCTGAGCTACTGCGGAGTTCATGCTTCTGAAAGGCTGGCTGAAATAGACAAGCCATACCTATTGAGATATCAACCCATAATGCGTACTGTTGGCCGAAAGTACTGCGTGGACCCTGCAGTGATTGCTGGTGTGTTGTCCAGGGAGTCTCATGGTGGCAACATTGTGGTCAACGTGGACAATGCAGGCGATGGAATCAGGGACCCTGGTCCCTATGCTCCCACATCCCAGATCAGCGAGTCCCAGCTTTCACGGATGACTCACATCCTCATTGTTAGAATCAAAGAAATCCAGAGGAGGTTTCCAACCTGGACCCCTGACCAGTACCTGAGAGGTGGACTCTGTGCCTACATTGGTGGTGCGAGCTACGTTAGAAGCCACCAGGACCTGAGCTGTGACTTCTGCAATGATGTCCTTGCAAGAGCCAAATACTTCAAGAGACATGGCTTCTAA

>Horse_LygA2

ATGCTTTCCTCTATCCTGTTTTTGGGACTTATTGCCGTTATTGGCACTTCCAGGGGCTCGTACCCTTTCACTCAGTCGATGAACCCTCACCTGCATCCCCGCCTGTACCATGGCTGCTATGGCGACATCATGATCATGGAGACGTCTGGCGCCTCCTGTGATGTAACCAAGTTGCTTAACTGTGGGATTCCCGGTTCCGAAATGTTTGCCGAGATGGATTTGAGGGCCTTAAAGCCTTACCGGGTTCTGATCAAAGAAGTCGGGCTGAGGCACTGCGTGGACCCTGCTCTCATTGCAGCCATCATCTCCAGAGAAAGCCATGGAGGAGTCGTCCTGAAAGACGGCTGGGACCACAAGGGACTTAAATTTGGCTTAATGCAGCTTGATAAAAAAAGTCATCACCCCGTTGGTACCTGGGACAGCAAAGAACACCTTTTGCAAGCTGTTGGGATTCTAACAGACCGATTTAAGGCAATCCAGAAAAAATTCCCCACGTGGAGTGTGACTGAACACCTCAAAGGTGGTCTCTCTGCCTTTAAGTCAGGAATTGATGCCATTGCCACCCCCGCAGACATAGACAATGACTTCGTCAATGACATTATTGCCCGAGCTAAATACTATAAAAGACACGGCTACTAG

>Guinea_pig_LygA1

ATGTCCGCATTGTGGCTGCTTCTAGGCCTCCTAGCCCTGATGGACTCATCGGAAAGCAGCAGTTGGGGTTGCTATGGGAACATCGGCACCCTGGACACCCCAGGAGCATCCTGCGGGACTGGAAGACGTTACGGCCTGACCTACTGCGGAGTCCGTGCTTCTGAAAGACTGGCTGAAATACACCTGCCTCACCTGCTGAGATACCAGCCCATCCTGCGGACGATCGGCCGGAAGTATTGCGTGGATCCTGCAGTGATCGCTGGGATCCTGTCCAGGGAGTCTCTCCGGGGCAGTGTTCCAATCAAAGTGGGCGTTCCCATCAATGTGGTGCAGGACACAAACGTCTTCACTCCCACATCCTGGATCAGCGAGCCCCAGGTTTCCCAGGTGACGAAGGTTCTGACCAATAGAATCAAAGAAATCCAGAGGACATTTCCAACCTGGACCCCCAGCCAGTACCTGAGAGGTGGGCTCTGTGCCTACAGCGGGGATGCTGGCCTCGTCAGAAGCAGCCAGGACCTGAGCTGCGACTTCTGCAATGATGTCCTTGCACGTGCCAAATACCTCAAGAGACACGGCTTCTAG

>Guinea_pig_LygA2

ATGCTGCCCTCCATCCTGCTTGTGGGACTTGTTGCTCTCATCGGCAAGAGCAGGAGCTCCTACCCTTTCCCTCATGCCCTGCGGCCTCATCTGCACCCCCGCCTGTACCACGGCTGCTACGGGGACATCATGACCATGGAGACCGCCGGGGTGCGCTGTGACATCAGCAATCTGCTCACCTGCGGGATCCGCGGCTCTGAAATGTTTGCCGACATGGACCTGCGCTCCATAAGGCCTTACCAGACCCTCATCAAAGAAGTGGGACAGAAGTACTGCGTGGACCCTGCCCTCATCGCAGCTGTCATCTCCAGAGAGAGCCACGGGGGCACCGCCCTGCAGCGCGGCTGGGACAGCAGGGGACTGAAGTTTGGCCTGATGCAGGTGGATAAAAGGTTTCAACCCACTGGTGCCTGGGACAGCAAAGAACACCTTTCACAGGCTGTGGATATCCTAGCAGAAAACATTAAAGCGATGCGGAGGAAGTTTCCCACGTGGAGTGTGGATCAACACCTCAAAGGTGGCCTATCTGCCTTTAAGTCAGGAGCCGAAGCCATTGCTACCCTGGAAGACATCGACTCCGACTATGCTGATGATGTGGTTGCCCGAGCTCGATTCTACAGGTCCCACGGCTTCTAG

>Chinese_Hamster_LygA1

ATGTCTCCACTGTGGCTGCTTCTGGGCCTCCTGGCCTTGACCGGTTCCTCTGAAAGCAGCAATTGGGGATGCTACGGAAACATCCGAACCCTGAATACCCCAGGAGCATCTTGCGTGATTGGAAGACGCCATGGCCTGCCTTATTGTGGAGTCCGTGCCTCTGAAAGGCTGGCTGAAATAGACAGGCCATATCTTCTGAGATATCAACCCACGATGAGAATTGTTGGCCGGAAATACTGTATGGATCCTGCAGTGATCGCTGGCGTCTTGTCAAGGGAGTCTCAGGGTGGCAATTTTGTGGTCGACATGGACAACGTGGGCAATGGAATCGGGGTGATACAGGACCCAAACTTCTACCCTCCCACATCCTGGAAGAGTGAGTCCTGGGTTTCCCAGAAAACTGAGATTCTGACATCTAAAATCAAAGAAATCCAGACTAGGTTTCCAACCTGGACCCCCGACCAGTACCTGAGAGGTGGACTCTGTGCCTACAGTAAGGGTCCTAGCTTTGTCCGAAGCAACCAGGATCTGAACTGTGACTTCTGCAATGATGTCCTTGCACGAGCCAAATACTTCAAGAACCACGGCTTCTAG

>Chinese_Hamster_LygA2

ATGCTACCCTCGGCGGTGTTTTGGGGACTTGTTGCCCTCATTGGCACTTCCAGGGGCTCATACCCATTAACACATTCCATGAACCCTCATCTGCATCCCCGACTTTACCATGGCTGCTATGGAGACATCATGACTATGGAAACCTTTGGGGCCCCCTGTGACATAAACAACCTGATGAACTGTGGGATCCACGGTTCGGAAATGTTTGCCGAGATGGACTTGAAAGCCATCAAGTCTTACCGTATCCTCATCAAAGAAGTGGGGCAGAGATACTGCATCGACCCCGCTATCATTGCAGCCATCATCTCCAGAGAAAGCCATGGAGGAGTTGTTCTGCAAAATGGCTGGGACCATAAGGGACAGAAATTTGGCTTGATGCAGCTTGATAAGACACACCATCCTATTGGTTCCTGGGACAGCAAAGAACACCTTCTACAGTCTGTTGGGATTCTAGCAGAAAAAATTAAGGCAATCCAGAGAAAATTTCCCACATGGAATGCGGCTCAGCACCTCAGAGGTGGTCTGGTTGCTTTTAAGTCAGGATTGGAAACCATTGTCACTCCTGCAGACATAGAAGCTGACTTAGTCGATGACATTATTGCCCGAGCTAAATTCTATAAAAGACATGGCTTCTAG

>Rabbit_LygA1

ATGTCTGCACTGTGGCTGCTCCTTGGTCTCCTTGCACTGACTGCCTTAGCTGAAAGCAGCACTTGGGGGTGCTATGGCAACATCCGCACCCTGGACACCCCTGGAGCATCTTGCGGGGTTGGAAGACGTCACGGCCTGAGCTACTGTGGAGTTCGGGCTTCTGAACGGCTGGCGGAAATAGACACGCCGCGCCTGCTGAGACATCAGCCCATGATGCGCGCTGTCGGCCAAAGGTACTGCGTGGATCCCGCGGTGGTCGCTGCCATCGTGTCCCGGGAGTCTCAGGGTGGCGGCGTTCCGGTCAACGTGGGCACCGTGGACGATGGAGTCGGAGAGGTGCAGGACCCTGCCATACATGGTAGCACACCTTGGATCAGCGAGCCCCAGGTCTCCCACATGACCGAGACTCTGACTGCTAGAATCAAAGAAATCCAGAGGAGGTTTCCCACCTGGACTCCTGACCAGTACCTGAGAGGTGGGCTGTGTGCCTATAGTGAGGGTGCTGGACGTGTCCGAAGCAGCCAGGACCTGAGTTGTGATTTCTGCAATGATGTCCTTGCTCGAGCCAAGTACCTCAAGAGGCGTGGCTTCTAG

>Rabbit_LygA2

ATGCTGACCCCTGTCATCCTTTTGGGATTTATTGCCCTCATTGGCACTTCCAGGGCCTCGTCTCCCTTCTTGCACTCAATAAACCCTCACCTGCATCCTCGCCTGTACCACGGCTGCTATGGGGACATCATGACCATGGAGACCTCTGGGGCGCTCTGTAACATAAAGAGTTTGCTTCACTGCGGGATACGTGGTTCAGAAATGTTTGCTGAGATGGATTTGAGGACCTTGAAGCCCTACCAGACTCTGATCAAAGAAGTGGGACAGAGGCATTGCGTAGACCCTGCTCTCGTCGCAGCCATCATCTCCAGAGAAAGCCACGGAGGAGCTGTTCTGCAAAACGGCTGGGACCACAGAGGCCTTAAATTCGGCCTGATGCAGCTTGATAAACAGATTTGCCACCCAATTGGTGCCTGGGACAGCAAAGAACACCTCTTACAGGGTGTTGAGATACTAGCAGACAGAATTAAGGCAATCCAGAAAAAATTCCCCACATGGAATATGGTTCAACATCTCAAAGGTGGACTCTCAGCCTTTAAGTCAGGAATCGAAGGCATTGACACACCTGCAGACATAGATGATGACTACGTCGATGATGTTATTGCCAGAGCTAAATTTTATAGACATCATGGTTACTAG

>Golden_hamster_LygA2

ATGCTACCCTCGGTGGTGTTCTGGGGACTTGTTGCCCTCATTGGCACTTCCAGGGGCTCACACCCATTCACGCATTCCATGAACCCTCATCTGCATCCCCGACTTTACCATGGCTGCTATGGAGACATCATGACCATGGAGACCTTTGGGGCCCCCTGTGACATAAATAACCTGTTGAACTGTGGGATCCACGGTTCGGAAATGTTTGCTGAGATGGATTTGAAAGCCATCACGCATTACCGTATCCTCATCAAAGAAGTGGGGCAGAGATACTGCATCGACCCAGCTATCATTGCAGCCATCATCTCCAGAGAAAGCCATGGAGGAGTTGTTCTGCAAAATGGCTGGGACCATAAGGGACAGAAATTTGGCTTGATGCAGCTTGATAAGACGCACCATCCTATTGGTTCCTGGGACAGCAAAGAACACCTTCTACAGTCTGTTGGGATTCTAGCAGAAAAAATTAAGGCAATCCAGAGAAAATTTCCCACATGGAACACAGCTCAGCACCTCAGAGGTGGTCTGGTTGCTTTTAAGTCAGGACTGGAAACCATTGTCACTCCTGAAGACATAGAAGTTGACTTAGTCGATGACGTTATTGCCCGAGCCAAATTCTATAAAAGACATGGCTTTTAG

>Chiru_LygA2

ATGCTATCTTTTGCCCTGTTTGGGGGCCTTTTTTTCCTCATTGGCGCTTCCTGGGGCTCACACTCCTTCACTCACACAATGAGTCCTCGCCTGCACTTCCGCCTGTACCATGGATGCTACGGCGACATCATGACCATGGAGACCCCTGGTGTCCCCTGTGATAACACCAGGATGATTGCCTGCGGCATCCGTGGTTCTGAGATGTTTGCTGAGATGGATTTGAAGGCCTTACAGTCTTATCAAATTCTGATCAAAGAAATTGGACTGCGGCACTGCGTGGACCCTGCTCTCATTGCAGCCATCATCTCTAGAGAAAGCCATGGTGGAACCATCCTGCTGGACGGCTGGGACCACACAGGACTTAAATTTGGCCTGATGCAGCTTGATAAAAACGTTCATCGTCCTGTTGGTACCTGGGATGGCAAAGAACACCTTTTGCAGGCTGTTGGGATTCTCACAGACAGAATTAAGGCAATCCAGAAAAGATTCCCCAGCTGGAGTGTGGCTCAGCACCTCAAAGGTGGTCTCTCAGCCTTCAAGTCAGGAACTGAAGCCATTGCCACCTCTGCAGACATACAGGCTGACTATGTCAATGATGTTTTAGCCCGAGCTAAGTTCTATAAGAAACATGGCTTCTAG

>Vole_LygA1

ATGTCTCCACTGTGGCTGCTTCTGGGTCTCCTGGCCTTGACTGGCTCCTCGGAAAGCAGCAATTGGGGATGCTACGGAAACATCCGAACCCTGAACACCCCAGGAGCATCATGCGTGATTGGAAAACGCTATGGCCTGCCTTACTGCGGAGTCCGTGCCTCTGAAAGGCTGGCTGAAATAGACAGGCCATATCTTCTGAGATATCAACCCACGATGAGAATCGTTGGCCGGAAATACTGTATGGATCCTGCAGTGATCGCTGGTGTCCTGTCAAGGGAGTCTCAGGGTGGCAATTTTGTGGTCGACATGGGCAACATGGGCAATGGAATCGGGGTGATACAGGACCCAAACTTCTACCCTCCCACATCTTGGAAGAGTGATTCCTGGGTTTCCCAGAAAACGGAGACTCTGGCATCTAAGATCAAAGAAGTCCAGGCTAGGTTTCCAAGCTGGAGCCCTGACCAGTACCTGAGAGGTGGTCTCTGTGCCTACAGTAAGGGTCCCAACTTTGTCCGAAGCAACCAGGATCTCAACTGTGACTTCTGCAATGATGTCCTTGCGAGAGCCAAATACTTCAAGAACCACGGCTTCTAG

>Vole_LygA2

ATGCTACCCTTGGCGGTGTTCTGGGCACTGGTTGCCCTCATTGGCACTTCCAGGGGCTCATACCCCCTCACTCATTCCATGAACCCTCAACTGCATGCCCGACTTTACCATGGCTGCTATGGAGACATCATGACCATGGAGACTTTTGGGGCCCCCTGTGACATAAACAATCTGATGAACTGTGGGGTCCACGGTTCCGAAATGTTTGCCGAGATGGACTTGAAAGCCATCCAGCCTTACCGTATCCTCATCAAAGAAGTGGGACAGAGATACTGCATCGACCCAGCTATCATCGCCGCCATCATCTCCAGAGAAAGCCATGGTGGAGTTGTCCTGCAAAATGGCTGGGACCATAGGGGACAGAAATTTGGTTTGATGCAGCTTGATAAAATGCACCATCCTATTGGCTCTTGGGACAGCAAAGAACACCTTCTACAGTCTGTTGGGATCCTAGCAGAGAAGATTAAGGCAATCCAGAGAAAATTTCCCACATGGAATTCAGCTCAGCACCTCAAAGGTGGTCTGGCTGCTTTTAAGTCAGGACTGGAAACCATTGTTACTCCTGCAGACATAGAAGCTGATTTAGTTGATGATCTTATTGCCCGAGCCAAATTCTATAAAAGACATGGCTTCTAG

>Rat_LygA1a

ATGTCTCCTCTGTGGCTGCTTCTGGGCCTCCTGGCTCTGACTGGCTCCTCTGAAAGCAGCAATTGGGGGTGCTATGGAAACATTCGCACCCTGGACACCCCAGGAGCATCCTGCAGGATTGGAAGACGCTATGGCCTGAGTTACTGTGGAGTCCGTGCCTCTGAACGGCTGGCTGAAGTAGACAGGCCATATCTTCTGAGATACCAACCCACCATGAGACTTGTTGGGCAGAAATACTGTATGGATCCTGCAGTGATCGCTGGTGTCCTGTCAAGGGAGTCTCCTGGTGGCAATTTTGTATTGGACATGGGCAACATGGGCAGTGGACTCGGGATGGTGAAGGAGTCAAAATTCTACCCTCCCACAGCTTGGAAGAGTGAGACCTGGGTTTCCCAGAAAACTCAGACTCTGACATCCAGCATCAAAGAGATCAAGACTAGGTTTCCAACCTGGACCACGGACCAGCACCTGAGAGGCGGACTCTGTGCCTACAGTAAAGGTCCCAACTTTGTCCGAAGCAACCAAGACCTGAACTGTGACTTCTGCAACGATGTCCTCGCGAGAGCCAAATACTTCAAGAACCATGGATTCTAG

>Rat_LygA2a

ACCCCTCGGTGGGTGTTCTGGGGACTTATTGCTCTCATTGGCACTGCCAGGGGCTCATATCCTGGGTTCACTCACTCCACAAACCCTCATGTGCACTCTCGTCTATACCATGGCTGCTACGGAGATGTCATGACCATGGAGACTTTTGGGGCTCCCTGTGACATAAACAATCTGATGAATTGTGGGATCCACGGTTCTGAAATGTTTGCCGAGATGGACTTGAAAGCCATAAAGCCTTACCGGATCCTCATCAAAGAAGTGGGACAGAGGCACTGCATCGACCCAGCCCTCATCGCTGCCATCATCTCCAGGGAAAGCCACGGCGGGGCTGTCCTGCAAAATGGCTGGGACCATAAAGGACAGAGGTTTGGCTTGATGCAGCTTGATAAAAACATGTACCATCCTATTGGTTCCTGGGACAGCAAGGAACACCTTCTACAGTCTGTTGGGATTCTGACAGAAAGAATTAAGGCACTGAAGAGGAAATTTCCTACCTGGAATGCAGCTCAGCACCTGAAAGGTGGTCTGACTGCTTTTAAGTCGGGGATGGAAACTATTGTCACTCCTGCAGACATAGAAGGCGACTTAGTGGATGACATCATTGCCCGTGCCAAATTCTATAAAAGACATGGCTTCTAG

>Rat_LygA1b

ATGTCTCCTCTGTGGCTGCTTCTGGGCCTCCTGGCTCTGACTGGCTCCTCTGAAAGCAGCAATTGGGGGTGCTATGGAAACATTCGCACCCTGGACACCCCAGGAGCATCCTGCAGGATTGGAAGACGCTATGGCCTGAGTTACTGTGGAGTCCGTGCCTCTGAACGGCTGGCTGAAGTAGACAGGCCATATCTTCTGAGATACCAACCCACCATGAGACTTGTTGGGCAGAAATACTGTATGGATCCTGCAGTGATCGCTGGTGTCCTGTCAAGGGAGTCTCCTGGTGGCAATTTTGTATTGGACATGGGCAACATGGGCAGTGGACTCGGGATGGTGAAGGAGTCAAAATTCTACCCTCCCACAGCTTGGAAGAGTGAGACCTGGGTTTCCCAGAAAACTCAGACTCTGACATCCAGCATCAAAGAGATCAAGACTAGGTTTCCAACCTGGACCACGGACCAGCACCTGAGAGGCGGACTCTGTGCCTACAGTAAAGGTCCCAACTTTGTCCGAAGCAACCAAGACCTGAACTGTGACTTCTGCAACGATGTCCTCGCGAGAGCCAAATACTTCAAGAACCATGGATTCTAG

>Rat_LygA2b

ATGGTACCCTCGGTGGTGTTCTGGGGACTTATTGCTCTCATTGGCACTGCCAGGGGCTCATATCCTGGGTTCACTCACTCCACAAACCCTCATGTGCACTCTCGTCTATACCATGGCTGCTACGGAGATGTCATGACCATGGAGACTTTTGGGGCTCCCTGTGACATAAACAATCTGATGAATTGTGGGATCCACGGTTCTGAAATGTTTGCCGAGATGGACTTGAAAGCCATAAAGCCTTACCGGATCCTCATCAAAGAAGTGGGACAGAGGCACTGCATCGACCCAGCCCTCATCGCTGCCATCATCTCCAGGGAAAGCCACGGCGGGGCTGTCCTGCAAAATGGCTGGGACCATAAAGGACAGAGGTTTGGCTTGATGCAGCTTGATAAAAACATGTACCATCCTATTGGTTCCTGGGACAGCAAGGAACACCTTCTACAGTCTGTTGGGATTCTGACAGAAAGAATTAAGGCACTGAAGAGGAAATTTCCTACCTGGAATGCAGCTCAGCACCTGAAAGGTGGTCTGACTGCTTTTAAGTCGGGGATGGAAACTATTGTCACTCCTGCAGACATAGAAGGCGACTTAGTGGATGACATCATTGCCCGTGCCAAATTCTATAAAAGACATGGCTTCTAG

>Tasmanian_devil_LygA1

ATGCTTATTTCCTTCCTGCTTGTGGGACTCGTTGCCCTTATTTACCCTACTGAAAGTAGTAATTGGGGATGCTATGGAAATATCAGAAACATCAATACCCCTGGAGCTTCATGTATAATTGGAAGACGACGAGGACTGAATTACTGTGGTGTTCGTGCTTCTGAAAGATTGGCTGAAATAGACTTGCCATATATACAGAGGTATCAGCCCACATTGAGGGTGATTGGCCAAAAATACTGTATGGATCCTGCTGTGATTGCTGGTATCATGTCTCGGCAGTCGCATGGAGGCAATGTCCTCGTCAGTGAGGGGAATATACCCAGTGGAAATGTTTTGGTGCAGACTGGTGGTATCCAGCACATTCCTACATCTTGGACCAGTGAAACCCAGGTTGCTCAGAATGTTGAAAGGCTAAATATGAGAATCAAAGAAATTCAGAGAAGATTCCCAACATGGACCACTGACCAGTATCTTCGAGGTGGACTTTGTGCCTACGATGAAGGGATAGGATATGTCAGAAGTAATCAAGACCTCGGATGTGACTTTTGCAATGATGTCCTGGCTCGAGCCAGATATTACAAGAGACATGGACTCTAA

>Tasmanian_devil_LygA2

ATGTTAGTTTCTGTCGCTTTTTTGGGCCTTGCTGTTCTCATGGTTACTTCTGAGAGCACTTACCCATATCCTCACCCAGTGAACCCTTACTCCCAGCCTCGGCTGTATCATGGTTGCTATGGTGATATCATGAGCATGGACACCCCAGGAGCCTCCTGTGCTATAGACAGATTGATCCACTGTGGGATCCGTGGCTCAGAAATGTTTGCTGAGATGGATTTGGTGCTTATGAAGAAATATCAAAATATAATAAAAAATGTTGGACAGAAACAATGTGTGGATCCTGCTTTGATTGCTGGAATTATCTCCAGGGAAACCCATGCTGGATCTGTGCTCCAAGATGGATGGGACCATCAGGGACTTAAATTTGGTCTAATGCAGCTTGATAAACATACTTATCATCCCACTGGAGCCTGGGACAGTGAAGAACACCTTATACAGGCTGTAAAGATCCTAACTGACAAAATTAAGGCAATCCAGAGAAAATTCCCAACCTGGACCATGACTCAACACCTGAAAGGTGGTCTCTCTGCCTACAAATCAGGGATTGATGCCATTGTAACCCCTAATGATGTAGAAAATGACTACACCAACGATATTCTTGCCCGATGTAAATTTTTTAAGAGGCATGGCTTCTAA

>Sheep_LygA2

ATGCTATCTTTTGCCCTGTTTGGGGGCCTTTTTGTCCTCATTGGCGCTTCCTGGGGCTCACACTCCTTCACTCACACAATGAGTCCTCGCCTGCACTTCCGCCTGTACCATGACTGCTACGGCGACATCATGACCATGGAGACCCCTGGTGTCCCCTGTGATAACACAAGGATGATTGCCTGCGGCATCCGTGGTTCTGAGATGTTTGCTGAGATGGATTTGAAGGCCTTACAGTCTTACCAAATTCTGATCAAAGAAATTGGACTGCGGCACTGCGTGGACCCTGCTCTCATTGCAGCCATCATCTCTAGAGAAAGCCATGGTGGAACCATCCTGCTGGATGGCTGGGACCACACAGGACTTAAATTTGGCCTGATGCAGCTTGATAAAAACATTCATCGTCCTGTTGGTACCTGGGATGGCAAAGAACACCTTTTGCAGGCTGTTGGGATTCTCACAGACAGAATTAAGGCAATCCAGAAAAAATTCCCCAGCTGGAGTGTGGCTCAGCACCTCAAAGGTGGTCTCTCAGCCTTCAAGTCAGGAACTGAAGCCATTGCCACCTCTGCAGACATACAGGCTGACTATGTCAATGATGTTTTAGCCCGAGCTAAGTTCTATAAGAAACATGGCTTCTAG

>Mouse_LygA1

ATGTCTCCACTGTGGCTGCTTCTAGGCCTCCTGGCCCTGACTGGCTCCTCTGAAAGCAGCAGTTGGGGATGCTATGGAAACATCCGCACCCTGGACACCCCAGGGGCCTCCTGCAGGATTGGAAGACGCTACGGCCTGACCTATTGCGGAGTCCGTGCCTCTGAACGGCTGGCCGAAGTTGACAGGCCATATCTTCTGAGACATCAACCCACCATGAGACTTGTTGGCCAGAAATACTGTATGGATCCTGCAGTGATCGCTGGTGTCCTGTCAAGAGAGTCTCCAGGAGGCAACTATGTGGTCGACCTGGGCAACATTGGCAGTGGACTTGGGATGGTGAAGGAGACAAAATTCTACCCTCCCACAGCTTGGAAGAGTGAGACCTGGGTTTCCCAGAAAACTCAGACTCTGACATCTAGCATCAAAGAGATCAAGACTAGGTTTCCAACCTGGACCGCCGACCAGCATCTGAGAGGTGGACTCTGTGCCTATAGTAAAGGTCCTAACTTTGTCCGAAGCAACCAGGACCTGAACTGTGATTTCTGCAATGACGTCCTTGCGCGAGCCAAATACTTCAAGGACCATGGCTTCTAG

>Mouse_LygA2

ATGGTGCCCTCGGTGGTGTTCTGGGGACTTATTGCTCTCGTTGGCACTGCCAAGGGCTCATATACTCACTCTGTGCACTCCATGAACCCTCATGTGCACCCTCGTCTGTACCATGGCTGCTATGGGGACATCATGACCATGGAGACTTTTGGGGCCCCCTGCGACATAAATAATCTGATGAACTGTGGGATCCACGGTTCGGAAATGTTTGCCGAGATGGATTTGAAAGCCATAAAACCTTACCGGATCCTCATCAAAGAAGTGGGGCAGAGACACTGTATTGACCCAGCCCTCATCGCTGCCATCATCTCCAGGGAAAGCCACGGCGGGGCTGTCCTGCAAAATGGCTGGGACCATAAGGGGCAGAGGTTTGGCTTGATGCAGCTTGATAAAAATATGTACCATCCTATTGGTTCCTGGGATAGCAAGGAACACCTTCTACAGTCTGTTGGGATTCTAACAGAAAGAATTAAGGCAATGAAGAGGAAATTTCCCACCTGGAATACAGCTCAGCAGCTGAAAGGTGGTCTGACTGCTTTTAAGTCGGGGATGGAAACTATTGTCACTCCTGCAGACATAGACGGTGACTTAGTGGATGATGTCCTTGCCCGGGCCAAATTCTATAAGAGACATGGCTTCTAG

>Goat_LygA2

ATGCTATCTTTTGCCCTGTTTGGGGGCCTTTTTGTCCTCATTGGCACTTCCTGGGGCTCACACTCCTTCACTCACACAATGAGTCCTCGCCTGCACTTCCGCCTGTACCATGGCTGCTACGGAGACATCATGACCATGGAGACCCCTGGTGTCCCCTGTGATAACACCAGGATGATTGCCTGCGGCATCCGTGGTTCTGAGATGTTTGCTGAGATGGATTTGAAGGCCTTACAGTCTTACCAAATTCTGATCAAAGAAATTGGACTGCGGCACTGCGTGGACCCTGCTCTCATTGCAGCCATCATCTCTAGAGAAAGCCACGGTGGAACCATCCTGCTGGATGGCTGGGACCACACAGGACTTAAATTTGGCCTGATGCAGCTTGATAAAAACATTCATCGTCCTGTTGGTACCTGGGATGGCAAAGAACACCTTTTGCAGGCTGTTGGGATTCTCACAGACAGAATTAAGGCAATCCAGAAAAAATTCCCCAGCTGGAGTGTGGCTCAGCACCTCAAAGGTGGTCTCTCGGCCTTCAAGTCAGGAACTGAAGCCATTGCCACCTCTGCAGACATACAGGCTGACTATGTCAATGATGTTTTAGCCCGAGCTAAGTTCTATAAGAAACATGGCTTCTAG

>Elephant_LygA1

ATGCCTGCGTGGTGGCTGCTCCTGGGCCTCGTAGCCCTCACTGACTTAACTGAAAGCAGCAATTGGGGATGCTATGGAAACATCCGGACCCTTGAAACCCCTGGGGTGTCTTGCAGTGTTGGAAGAGGACAAGGCCTTAACTACTGCGGAGTCCGTGCTTCTGAAAAGCTGGCTGAAATAGACATGCCACAGCTAGTGAAATACCAGCCCGCGATGCGAGTTGCTGGCCGGAAGTACTGCGTCGACCCTGCGCTCATAGCGGCCATCTTGTGCAGGCAGGCTCGCAGAGGCAACGTTCTGGTCGACGTGGGGAACGTGGACGATGGGGTCGGGGTGGTGCAGTACCCTGGTCTTCATGCTCCCACGTCTGGGATCAGCGAGTCCCAGGTTGCCCGGATGACTGAGGTCCTGATTGTTAAAATCAAAGAGATCCAGAGGAGGTTTCCAAGCTGGACCCCGGACCAGCATCTGAGAGGTGAACTCTGTTCCTACGATGGAGGTGTTGGCTATGTCAGAAGTAGCCAGGACCTGAGCTGCGACTTCTGCAATGATGTCCTTGCACGAGCGAAATACTACAAGAGACGTGGCTTCTAA

>Elephant_LygA2

ATGCTATCCTCTGTCGTGTTTTGGGGACTCATTGCCCTCATTGGTACTTCCAGGGGCTCCTACCCTTTCACTCACATGATGAACCCTCACCTGCATCCCCGTCTGTACCACGGGTGCTATGGGGACATCACCACCATGGACACCTCGGGTGCCACCTGTGATACAGACAGGGTGATCAACTGTGGGATCCGTGGTTCTGAAATGTTTGCTGACATGGATTTGAGGGCTATAAAGCTTTACCAGGTTATGATCAAAGATGTTGGGCAGAGGCTCTGCGTGGACCCAGCCCTTGTTGCAGCCGTCATCTCCAGGGAAAGCCACGGTGGAGCCGCCCTGCAGAGTGGCTGGGACCACAGTGGGCTGAAATTTGGTTTGATGCAGCTCGATAAGAAAATCCATGACCCCGTTGGTGCCTGGGACAGCAAAGAACACCTTTTTCAGGGTGTTGGGATTCTAACAGACAGAATTAAGGCAATCCAGAAAAAGTTCCCCACGTGGAGTGTGGCTCAACACCTCAAAGGTGGTCTTTCGGCCTTTAAGTCAGGAATCGAAACCATTGTCACCCCCGTGGACATAGACACCGACTACGTCAACGATCTTCTTGCCCGAGCTAAATTCTACAAAAGACATGGCTTCTAG

>Cow_LygA2

ATGCTATCTTTTGCCCTGTTTGGGGGCCTATTTGTCCTCATTGGCACTTCTAGGGGCTCACACTCCTTCACTCACACAATGAGTCCTCGCCTGCACTTCCGCCTGTACCATGGCTGCTACGGAGACGTCATGATCATGGAGACCCCTGGTATCCCCTGTGATAACACCAGGATGATTGCCTGTGGCATCCGTGGTTCTGAGATGTTTGCTGAGATGGATTTGAAGGCCTTACAGTCTTATCAAATTCTGATCAAAGAAATTGGACTGCGGCACTGCGTGGACCCTGCTCTCATTGCAGCCATCATCTCTAGAGAAAGCCACGGTGGAACCATCCTGCTGGATGGCTGGGACCACACAGGACTTAAATTTGGCCTGATGCAGCTTGATAAAAAAATTCATCATCCTGTTGGTACCTGGGATGGCAAAGAACACCTTTTGCAGGCTGTTGGGATTCTCACAGACAGTATTAAGGCAATCCAGAAAAAATTCCCCAGCTGGAGTGTGGCTCAACACCTCAAAGGTGGTCTCTCAGCCTTCAAGTCAGGAACTGAAGCCATTGCCACCTCCGCAGACATACAGGCTGACTATGTCAATGATGTTTTAGCCCGAGCTAAGTTCTATAAGAAGCGCGGCTTCTAG

>Opossum_LygA1

ATGCTTCTTTCCTGGCTGCTTCTGGGTCTGGCTGCCCTTATTTACCCTGCTGAAAGTAGTAATTGGGGATGCTACGGGAACATCAGAAATATCGATACTCCTGGAGCTTCATGTTTAATTGCAAGAAGACGAGGGCTGAATTTCTGCGGAGTCCGGGCTTCCGAAAGGCTGGCTGAAATGGACTTGCCGTACGTGCAGAGATATCAGCCCACACTGAGGCTAGTTGGCCGAAAATACTGTTTGGATCCTGCTGTGATCGCCGGCATCCTGTCCCGGGAATCACAAGGAGGCAATGTCCTTGTCAGCGGGGGGAATGTAGCCAGCGGAATTGGTTTGCCTGGTGGTATCCAGCAAATTCCTACATATTGGACCAGTGAAACGCGGGCTAGTCAGGTTTCCGAAAATCTGAATATGAGAATCAAAGAGATTCGGAGAAGATACCCAACGATCTCTGCTGACCAGTATCTGAGAGGTGGACTCTGTGCCTACAGTGAAGGACCAGGATATGTCAGAAGTAATCAGGACTTCAGCTGTGACTTTTGCAATGACGTCCTGGCTCGAGCCAGATATTACAAGGGTCATGGATTCTAA

>Opossum_LygA2

ATGTTAATTCCTGTCACTTTTTTGGGTCTTGCTGTCCTCATTGCTCCGTCTGAGGGCACTTATTCACATCCCCAACCCGTGAATCCCCCCTTCCAGCCTCATCTGTACCATGGTTGCTATGGTGATGTCATGAGAATGGATACCCCAGGAGCCTCTTGTAACACAGACAGATTGATCCACTGTGGGATCCGTGGCTCAGAACTGTTTGCTGAGATGGATTTGGCACTTATGATGAAATATCAAACTATGATCAAAACTGTTGGACAGAAACAATGTGTAGATCCTGCTTTGATTGCTGCAATTATCTCCAGAGAGACCCATGCTGGGTCCGTGCTACAAGATGGCTGGGACCATCAAGGACTTAAATTTGGTTTGATGCAGCTTGATAAACATACTCATCACCCCACTGGAGCCTGGGATAGTCAAGAGCACCTGACACAGGCTGTAAAGATCCTAACTGACACAATTAAGACAATCCAGAGAAAATTCCCAACATGGAGCATGAGTCAACACCTGAAAGGTGGCCTCTATGCCTACAGATCAGGGATTGATGCCCTTGTAACACCTACTGATGTAGAAAATGACTACACCAATGATATTCTTGCCCGATGTAAATTTTATAGGAGACACGGCTTCTAA

>Aardvark_LygA1

ATGTCTCCATTGTGGCTGCTTCTGGGCCTCCTTGCCCTTACTGCCTTATGTGAAAGTAGCAATTGGGGATGCTATGGAAATATCCGAACCATCGAAACTCCTGGAGCATCTTGTGGGGTTGGAAGACCACGAGGCCTCAACTACTGTGGAGTGCATGCTTCTGAAAGACTGGCTGAAATAGACATGCCACAGTTAGTGAGATATCAACCCATTATGCGAACTGTTGGCCAGAAATACTGCGTGGATCCTGCAGTCATTGCAGCCATCTTGTCCAGGCAGTCTCATGGAGGCAACGTTCTGGTCAATGCGGGGAATGCGGCTCATGGAATCCAAGACCCTGGCCATTTTGCTCCCACATCCTGGATCAGTGAGTCCCAGGTTGCCAAGATGACTGAGGTTCTGACTATTAGAATCAAAGAAATCCAAAGAAGGTTTCCAACCTGGACCCCAGATCAGTATCTGAGAGGTGGCCTCTGTTCTTACAATGGAGGTGTTGGCTATGTCAGAAGTAGCCAGGACCTGAGCTGTGACTTCTGCAATGATGTCCTTGCACGAGCCAAATACTACAAGAGACATGGCTTC

>Aardvark_LygA2

ATGCTATCCTCCATAGTGTTTTGGGGACTCATTGCCCTCATTGGTCCTTCCAAGGGCTCGTACCCTTTTACTCACATGATGAACCCTCACCTGCATCCTCGTGTGTACCATGGGTGCTATGGGGACATCACGACTATGGAGACCTCTGGGGCATCCTGTGATATAAGCCAGCTGATTAGCTGTGGGATCCGTGGTTCTGAAATGTTTGCTGAGATGGATTTGAAGGCCATAAAGACTTACCAAGTTATGATCAAAGAAGTTGGACAGAGGCACTGTGTGGACCCAGCCCTCATTGCAGCCATCATCTCCAGAGAAAGCCATGGTGGAGCTGCCCTGCAAGATGGCTGGAACCACAATGGACTTAAATTTGGCTTGATGCAGCTTGATAAGAAAATATATCACCCTGCTGGTGCCTGGGACAGCAAAGAACACCTTTTGCAGGCTGTTGGGATTCTAACAGACAAAATTAAGGCGATCCAGAAAAAATTCCCCATGTGGAGTGTGGCTCAGCACCTCAAAGGTGGTCTTTCAGCCTTTAAGTCAGGAATCGAAACCATTATCACCCCCATGGACACAGACACTGAGTTCGTCAATGATCTTCTTGGTCGAGCTAAATTCTATAAAAGGCATGGGTTC

>Yak_LygA2

ATGCTATCTTTTGCCCTGTTTGGGGGCCTATTTGTCCTCATTGGCACTTCTAGGGGCTCACACTCCTTCACTCACACAATGAGTCCTCGCCTGCACTTCCGCCTGTACCATGGCTGCTACGGAGACGTCATGATCATGGAGACCCCTGGTATCCCCTGTGATAACACCAGGATGATTGCCTGCGGCATCCGTGGTTCTGAGATGTTTGCTGAGATGGATTTGAAGGCCTTACAGTCTTACCAAATTCTGATCAAAGAAATTGGACTACGGCACTGCGTGGACCCTGCTCTCATTGCAGCCATCATCTCTAGAGAAAGCCACGGTGGAACCATCCTGCTGGATGGCTGGGACCACACAGGACTTAAATTTGGCCTGATGCAGCTTGATAAAAAAATTCATCATCCTGTTGGTACCTGGGATGGCAAAGAACACCTTTTGCAGGCTGTTGGGATTCTCACAGACAGTATTAAGGCAATCCAGAAAAAATTCCCCAGCTGGAGTGTGGCTCAACACCTCAAAGGTGGTCTCTCAGCCTTCAAGTCAGGAACTGAAGCCATTGCCACCTCCGCAGACATACAGGCTGACTATGTCAATGATGTTTTAGCCCGAGCTAAGTTCTATAAGAAGCGCGGCTTCTAG

>Pig_LygA2

ATGCTATCTGTTGTCCTGTTTGGGGGACTTTTCGCCCTCATTGGCACTTCCAGGGGCTCACAGTCCTTCACTTACTCCATGAACTCTCGCCTGCATCCCCGCCTGTACCATGGCTGCTATGGTGACATCATGACCATGGAGACCTCTGGTGCAGCCTGTGACATCAACAGGTTGATATCCTGCGGCATCCACGGTTCTGAAATGTTTGCTGAGATGGATCTGAGGGCCTTGAAGCCTTACCGAACGCTGATCAAAGAAGTCGGGCTGAGGCACTGTGTGGACCCAGCTCTCATTGCTGCCATCATCTCCAGAGAAAGCCATGGGGGCACCATCCTGCTAGATGGCTGGGACCACAGAGGACTTAAATTTGGCTTGATGCAGCTCGATAAAAAAATTCATCACCCGGTTGGTACCTGGGACAGCAAAGAACACCTTTTGCAGGCTGTTGGGATTCTAACAGACAGAATTAAGGCAATCCAGAAAAAATTTCCCACGTGGAGTGTGACTCAACACCTCAAAGGTGGTCTCTCAGCCTTCAAATTGGGAACTGAATCCATTGCCAGCCTCACGGACATAGAGGATGACCTCGTCAATGATATTTTAGCCCGAGCTAAATTCTATAAGAGACATGGCTTCTAG

>Alpaca_LygA2

ATGCTCTCTTTTGCCCTGTTTGGGGGACTTTTCGCCCTCATTGGCACTTCCAGGGGCTCACATACTTTCACTCATTCAATGAACTCTCGCCTGCATCCCCCCCTGTACCACGGCTGCTATGGTGACATCACGACCATGGAGACCTCTGGTGCCTCCTGTGATATTGGCAAGTTGATTAATTGCGGCATCCCTGGTTCTGAAATGTTTGCCGACATGGATTTGAGGGCCTTAAAGCCTTACCGGACTCTGATCAAAGAAGTCGGGCTGAGGCACTGCGTGGACCCTGCCCTCATTGCAGCCATCATCTCCAGAGTAAGCCACGGCGGAACTGTCCTGCTAGACGGCTGGGACCACACAGGAAATAAGTTTGGCTTGATGCAGCTTGATAAAAATATTCATCAACCTGCTGGCACCTGGGATAGCAAAGAACACCTTCTGCAGGCTGTTGGGATTCTAACGGACAGAATTAAGGCAATCCAGAAAAAGTTCCCCTCGTGGAGTGAGGCTCAGCACCTCAAAGGTGGTCTCTCAGCCTTCAAGTCAGGAGCTGAAGCCGTTGCCACCCTCAAGGACATAGACACTGACTTCGTCAATGATACTATGGCCCGCGCTAAATTCTTTAAGAGACATGGCTTCTAG

>Camel_LygA2

ATGCTCTCTTTTGCCCTGTTTGGGGGACTTTTTGCCCTCATTGGCACTTCCAGGGGCTCACATACTTTCACTCATTCAATGAACTCTCGCCTGCATCCCCCCCTGTACCACGGCTGCTATGGTGACATCACGACCATGGAGGCCTCTGGCGCCTCCTGTGATATTGGCAAGTTGATTAATTGCGGCATCCCTGGTTCTGAAATGTTTGCCGACATGGATTTGAGGGCCTTAAAGCCTTACCAGACTCTGATCAAAGAAGTCGGTCTGAGGCACTGCGTGGACCCTGCCCTCATTGCAGCCATCATCTCCAGATTAAGCCACGGCGGAACTGTCCTGCTAGACGGCTGGGACCACACAGGAAATAAGTTTGGCTTGATGCAGCTTGATAAAAATATTCATCAACCTGCTGCCACCTGGGATAGCAAAGAACACCTTCTGCAGGCTGTTGGGATTCTAACGGACAGAATTAAGGCAATCCAGAAAAAGTTCCCCTCGTGGAGTGAGGCTCAGCACCTCAAAGGTGGTCTCTCAGCCTTCAAGTCAGGAGCTGAAGCCGTTGCCACCCTCAAGGACATAGACACTGACTTCGTCAATGATACTATGGCCCGCGCTAAATTCTTTAAGAGACACGGCTTCTAG

>Wallaby_LygA2

ACTTCTGAGGACACTTCTCCATATCCTCCCCCTGTGAATTCCCACTTCCAGCCTCATCTGTACCATGGTTGCTATGGTGATATCATGAGTATGGACACCCCAGGAGCCTCCTGTGATATAGACAGATTGATCAACTGTGGGATCTCTGGCTCAGAAATGTTTGCTGAGATGGATTTGGTGCCTATGAAGAAGTATGAAACTATAATTAAAGATGTTGGACAGAAACAATGTGTTGATCCTGCTTTGATTGCTGCAATTATCTCCAGAGAGACCCACGCTGGGTCTGCGCTCCAAGATGGCTGGGACTATCAGGGACTGAAATTTGGCCTGATGCAGCTTGATAAACACACTTATCAGCCCACTGGAGCCTGGGACGGTGAAGAGCACCTTACACAGGCTGTAAGGATCCTAACTGACAAAATTAAGGCAATCCAGAGAAAATTCCCAACCTGGACCATGAGTCAACACCTGAAAGGTGGTCTCTCTGCCTACAGATCAGGGATTGATGACATTGGAACCCCTAGTGATGTAGAAAATGACTACACCAACGATATTCTTGCCCGATGTAAATTTTATAAGAGACATGGATTCTAA

>Chicken_LygC

ATGTCAGGCTGTTCTAATTTCTATGGGAACATAGCAAATGTTGAAACAACTGGTGCATCACAGAGAACTGCGAAGCCGGAAGGTCTGAGCTATGCAGGAGTTGCGGCTTCAGAGAAGATTGCTGAAAGAGATTTGAAGAATATGGACAAATATAAAGAAACTATTACAAAAGTGGCCAACAGCAAGTGCATTCCACCATCTTTGGTTGCTGCTGTTATCTCTCGAGAGTCACACGCTGGGACGGCACTGAAGGATGGCTGGGGTGACCACGGTAATGCATTTGGTTTAATGCAGGTTGACAAACGGTACCATAAACCTCATGGGGCATGGGACAGTGAAGAGCACATAAAACAAGGCACAGACATTTTGTGTCAGTCAATAACCGATATTCAGAAAAAATTCCCAACATGGAGTAAGGAACAGCAGCTCAAAGGTGGTATTTCAGCCTATAATGCAGGAACAAGAAATGTCCGGACCTATGAAGGAATGGATGTTGGCACAACACACGACGACTATGCCAACGATGTGGTTGCAAGAGCCAAGTTCTTTCAGAGAAATGG

ATACTGA

>Turkey_LygC

ATGTCAGGCTGTTCTAATTTCTACGGGAACATAGCAAATGTTGAAACAACTGGTGCATCAGAGAGAACTGCGAAGCCGGAAGGTCTGGGCTATGCAGGAGTTGCTGCTTCAGAGAAGATTGCTGAAAGAGATTTGAAGAACATGGACAAATATAAAGAAACTATCACAAAAGTGGCCAACAGCAAGTGTGTTTCACCATCTGTGGTTGCTGCTGTTATCTCTCGAGAGTCACACGCTGGGACAGCACTGAAGGATGGCTGGGGTGACCACGGAAATGCATTTGGTTTAATGCAGATTGACAAACGGTACCATCAGATTCATGGTGCATGGAACAGTGAAGAGCACATAAAACAAGGCACAGACATTTTGTGTCAGTCAATAAACGATATTCAGAAAAAATTCCCAACATGGAGTAAGGAACAGCAGCTCAAAGGTGGGATTTCAGCCTATAATGCAGGAACAAGAAATGTCCGGACCTATGAAGGAATGGATGTTGGCACAACACACAACGACTATGCCAACGATGTGGTTGCAAGAGCCAAGTTCTTTCAGAGACATGG

ATACTGA

>Chicken_LygB

ATGTGCCTGGTGCTCATGCTGCTGGGCCTCACCGCCCTCCTGGGTATCTGCCAGGGCGGCACGGGTTGCTACGGCAGTGTAAGCAGAATCGATACCACAGGGGCTTCATGCAGAACTGCAAAACCAGAGGGCTTAAGCTACTGTGGAGTTCGAGCTTCAAGAACGATTGCTGAGCGGGACCTAGGAAGTATGAATAAATACAAAGTACTCATTAAGAGAGTTGGCGAAGCCTTATGTATTGAGCCTGCTGTGATTGCTGGCATCATCTCTCGGGAATCTCACGCTGGCAAAATACTGAAGAATGGCTGGGGTGACCGTGGAAATGGATTTGGTTTAATGCAGGTTGACAAAAGATATCATAAAATTGAGGGAACGTGGAATGGAGAAGCCCATATCAGGCAGGGCACAAGAATACTTATCGACATGGTAAAGAAAATACAGAGAAAATTCCCACGCTGGACAAGGGATCAACAGCTAAAAGGTGGGATTTCTGCCTACAATGCTGGTGTTGGGAATGTCCGAAGCTATGAGAGAATGGACATCGGCACTCTCCATGATGACTACAGCAATGATGTGGTTGCTCGAGCCCAGTATTTCAAGCAGCATGGATACTAG

>Turkey_LygB

ATGCGCCTGGTGCTCACACTGCTGGGCTTCACCGCCCTCCTGGGTATCTGCCAGGCCCGCACGGATTGCTATGGCAATGTAAACAGAATTGATACCACGGGTGCTTCATGCAAAACTGCAAAACCAGAGGGCTTAAACTACTGTGGAGTTCAAGCTTCAAAAAAGATTGCTGAACGGGACCTAGGAAATATGAATAGATACAAAACACTCATTAAGAGAGTTGGCGAAGCATTATGTATTGAGCCTGCTGTGATTGCTGGCATCATCTCTCGGGAATCTCACGCTGGCACAATACTGAAGAATGGCTGGGGTGACAATGGAAATGGATTTGGCTTAATGCAGGTTGACAAAAGATACCATAAAATTGAGGGAACATGGAATGGAGAAGCCCATATCAGGCAGGGCACGAGAATACTTATTGACATGATAAAGAAAATACAGAGAAAATTCCCACGTTGGACAAGGGATCAACAGCTAAAAGGTGGGATTTCTGCCTACAACGCTGGTACTGGGAATGTCCGAAGCTATGAGAGAATGGACATCGGCACTCTCCATGATGACTATGCCAATGATGTGGTTGCTCGAGCCCAGTATTTCAAGCAGCACGGATACTAG

>Duck_LygB

ATGAGCCTGACGCTCGTGCTGCTGGGCCTTGCTGCCCTCCTGGGTGCTTGCCAGGGCCGCACGGATTGCTACGGCAGTGTAAACAGAATTGACACCACCGGGGCTTCATGCAAAACTGCAAAACCAGAGGGCTTAACCTACTGCGGAGTTCCAGCTTCAAAAAAGATTGCTGAACGAGACCTGAAAGCTATGGATAAATACAAAACACTCATTAAGAAAGTGGGTGAAAAGCTGTGTATTGAGCCCGCTGTGATTGCTGGTATCATCTCTCGTGAATCTCATGCTGGTAAAATACTCAAGGGCGGCTGGGGTGACAATGGAAATGGATTTGGCTTAATGCAGGTTGACAAAAGATACCATAAACTCCAGGGGACGTGGAATGGAGAAGCCCATATCACTCAGGGCACGACGATACTTATCAATTTTATAAAGAGAATACAGAAGAAGTTCTCAAACTGGACAAAGGACCAGCAGCTGAAAGGTGGGATTTCTGCCTACAATGCCGGAGACGGGAACGTCCGAACCTATGAGAGGATGGACATTGGCACTACCCATGACGACTACGCCAACGACGTGGTCGCTCGAGCCCAGTATTACAAGCAGCACGGATACTAG

>Chicken_LygA

ATGGTCACCGCGCTGCTCTTGCTGGGCCTTTCGGCCCTGTTTGCTCCATCCATGAGCTACAGTTGCTATGGCGATATCAGCGCTCTTCAGGCACCCACAATTTCCTGCACAGCCGTAAGAGCCCAGGACTGTGGCCTTGCTGCTCTGAGGAGGACGGTGGAAGCGGATGTCATCCGCCTGAGGAGATACGAGGTGCCCATTAAGAGAGTGGCCAGAAGGCTGTGCTTGGACCCAGCTCTCATCGCAGCCATCATCTCTCAGGAGAGCCGTGCTGGCCTGCTGCTGGACAACGGCTGGGACCAGGGCCGGCAGCGGTACGGCCTGATGCAGATTGACAGGAGGTATTATCAGCCCTTTGGGATGTGGGACAGCGAGGAGCACATAAACCAGTGTTCGAGCATGCTGGTGGCAGGAATCAACGAAGTGAGGGCAAGGCACCCTGCCTGGAGCTGGGACCAGCAGCTGCGAGGGGGAATTTGCACCTACCATGCAAGAGCCGGCAACCTCCAGATCTACGATGAAGACCCCTGCAGCAGAGACAACAACTATGTCAACAGCGTGATCAGGAGGGCCCAGTACCTCAAGAGAAATGGGTTCTAG

>Turkey_LygA

ATGGTCACTGCGCTGCTTTTGCTGGGCCTTTCGGCCCTGTTTGCTCCATCTATGAGCTACAGTTGCTATGGTGATATCAGTGCTCTTCAAGCACCCACAATTTCCTGCACAGCCGTAAGAGCTCAGGACTGCGGCCCTGCTGCTCTGAGGAGGACGGTGGAAGCGGACGTCATTCGCTTGAGGAAATACGAGGTGCCCATTAAAAGAGTGGCCAGAAGGCTGTGCCTGGACCCGGCGCTCATTGCTGCCATCATCTCTCAGGAGAGCCGTGCCGGCCTGCTGCTGAACAATGGCTGGGACCAGGGCCAGCAGCGGTACGGCCTGATGCAGATTGACAGGAGGTATTATCAGCCCTTTGGGACGTGGGACAGTGAGGAGCACATAAACCAGTGTTCGAGCATGCTGGTGGCAGGAATCAACGAAGTGAGGGCAAGGCACCCTGCCTGGAGCTGGGACCAGCAGCTGCGAGGGGGAATTTGCACCTACCATGCAAGAGCCGGCAACCTCCAGATCTATGATGAAGACCCCTGCAGCAGAGACAACAACTATGTCAACAGTGTGATCAGCAGGGCCCAATACTTCAAGAGAAATGGGTTCTAG

>Duck_LygA

ATGGTCCCCACGCTGCTCTTGCTGGGCCTTTCGGCCCTGATCGCTCCGTCTGCGAGTTACAGTTGCTATGGCGATATAAATGCTCTTCAAGCCCCCACGATTTCCTGCACAGCCGTGAGAGCCCCGGACTGCGGCATTGCTGCTGTGAGGAGGACGGCGGATGCGGACATCATTCGCCTGAGGAAATACGAGATCCCCATTAAGAGAGTCGCCAGGAACCTGTGCCTGGACCCGGCGCTCATCGCTGCCATCATCTCGCAGGAGAGCCGTGCCGGCCTGCTGCTGGACAACGGCTGGGACCAGGGCCGGCAGAGGTTCGGCCTGATGCAGATCGACAGGCGCTACCACCAGCCTTACGGGACGTGGGACAGCGAGGAGCACATCAACCAGTGTTCGACCATGCTGGTGCTTGGGATCAATGAAATGCGGGTGAGGCACCCTACCTGGACCTGGGACCAGCAGCTGAGAGGGGGAATCTGCGCCTACCGCGCAAGAATCGGCAACATCCAGGTCTACGACGAAGACCCCTGCGGCAGAGACAACTCCTACGTCAACAGCGTGATCGGGCGGGCCCAGTACTTCAAGAGGCATGGGTTCTAG

>Zebra_finch_LygA

ATGATCCCCGCACTGCTGTTGCTGGGTCTTTCAGCCCTTGTTGCTCCATCCATGAGCTACAGTTGCTATGGTGATATAAGTGCTCTTCAAGCCCCCACGATCTCCTGTACACCTGCAAGAGCCTCTGATTGTGGATATGCCATGATACGGAGGACTGCTGAGGCAGACCTCACACGCCTGAGGAGATACGAGATCCCAATTAAGAGAGTAGCCAGAAACCTCTGCTTGGACCCAGCGCTCATCGGAGGCATCATGTCCCAGGAGAGCCGTGTCGGCCTGCTCCTGGACAACGGCTGGGACCAGGGACGCCAGAAGTACGGCCTGATGCAGATCAGCAGGCAGCAACTGCAACCTTATGTGGCGTGGGATAGTGAAGAACACATAAATCAATGCTCAAATATCCTGGTTCTTTCCATTAATGAAGTACGGGCAAGACATCCTACCTGGACCTGGGACCGGCAGCTGAGAGGGGGAATCTGCACCTACCATGCAAGAATGGGCAACCTCCAGGTCTACGAGGAGGACCCATGCAGCAGCAACTACAACTACGTCAACAGCGTGATTAGGCGAGCCCAATACTTTAAGAGAAATGGGTTCTAG

>Ostrich__LygB

ATGCACCTGATGCTTGTGCTGCTGGGCCTTGCTGCCCTCCTAGGTACATCTCAGAGCCGAACTGGCTGCTACGGTGATGTAAACAGAGTTGACACCACTGGGGCTTCGTGTAAAAGTGCAAAGCCAGAGAAATTAAACTACTGCGGAGTTGCAGCTTCAAGAAAGATTGCTGAACGGGACCTACAAAGTATGGATAGATATAAAGCTCTCATCAAGAAAGTTGGCCAAAAACTATGTGTCGATCCGGCTGTGATTGCTGGCATTATCTCTCGAGAATCTCATGCTGGCAAAGCACTGAGGAATGGCTGGGGGGACAATGGAAATGGATTTGGTTTAATGCAGGTTGACAGAAGATCACATAAACCTGTGGGAGAATGGAATGGAGAAAGACATCTTATGCAGGGCACAGAAATACTTATCTCGATGATAAAGGCAATACAGAAAAAGTTCCCACGCTGGACAAAGGAACAACAGCTGAAAGGTGGGATTTCTGCCTACAATGCTGGTCCTGGGAATGTCCGAAGCTATGAAAGAATGGATATCGGCACTACTCATGATGACTATGCCAACGATGTGGTAGCACGAGCCCAGTATTATAAGCAACATGGATACTAG

>Emu_LygB

ATGCATCTGATGCTTGTACTGCTGGGCCTCGCTGCCCTCCTGGGTACATCTCAGAGCCAGACTGGCTGCTACGGTGTTGTAAACAGAATTGACACCACTGGGGCTTCATGTGAAACTGCGAAACCAGAGAAATTAAACTACTGCGGAGTTGCAGCTTCAAGAATGATTGCAGAAAGGGACCTACGAAGTATGGATAGATATAAAACTCTCATTAAGAAAGTTGGTCAAAAACTGTGTGTCGATCCAGCTGTGATTGCTGGCATTATCTCTCGAGAATCTCATGCTGGCAAAGCACTGAAGAACGGCTGGGGTGACAATGGAAATGGATTTGGTTTAATGCAGGTTGACAAAAGATCACATACACCTGTGGGAGAATGGAATGGAGAAAGACATCTTACACAGGGCACAGAAATACTTATCTCAATGATAAAGAAAATACAGAAAAAGTTCCCACGCTGGACGAAGGAACAACAGCTGAAAGGTGGGATTTCTGCCTACAATGCTGGTTCTGGGAATGTCCGCAGCTATGAAAGAATGGATATTGGCACTACCCATAATGACTATGCCAACGATGTGGTTGCACGAGCCCAGTATTATAAGCAACATGGATACTAG

>Pigeon_LygC

ATGTCAGGCTGTTTGGGTCATTATGGGAATATACTGAATGTTGAAACAACTGGAGCTTCAGAGGCAACTGCGAAACCAGAAGGTCTGAGCTACTCAGGAGTTCTTGCCTCAGAGAAGATTGCAGAAAAAGATTTAAAGAACATGGAGAAATACAAAGCCAAGATTACAGAAGTTGGCAACAGCAAGTGTGTTGATCCAGCTGTGATTGCTGGTATTATCTCTCGAGAGTCACATGCTGGGTCAGTGCTGAAAGACGGCTGGGGTGACCATGGAAATGCATTTGGTTTAATGCAGGTTGACAAACGGTACCATAAGATCGTCGGGTCATGGGACAGTAAAGAGCACATAGCGCAAGGCACAGAGATTTTATGCGGGATGATAAACGAAATTGAGAGGAAATTCCCAACTTGGACAAAGGAACAGCAGCTCAAAGGTGGGATCTCAGCCTATAACGCGGGAGCAAACAATGTCCGGAGCTACGACAGGATGGATATTGGCACAACACACCATGACTATGCCAATGACGTGGTTGCAAGAGCCAAGTTTTATAAGAGAAATGGATATTGA

>Pigeon_LygB

ATGCTCGTGCTGCTTGTCCTCACTGCCCTCCTGGGTGTCTCCCAGGGCCGCACGGATTGCTATGGCAGTGTAAGCAGAATTATAACCCCTGGGGCTTCGTGCAAAACTGCAGCGCCAGAGGGTTTATCCTACTGTGGAGTTAAGGCTTCAGAAAAGATTGCTGAACGGGACCTAGGTGCTATGAATCGATATAAAACACTCATTAAGAAAGTTGGTGAAAAACTCTGCATTGAACCAGCCCTGATAGCTGGCATCATCTCCCGGGAATCTCATGCTGGCAAAGTACTGAAGAATGGCTGGGGTGACAATGGAAATGGATTTGGTTTAATGCAGGTTGACAAAAACTCACATAGACCTGTGGGACAATGGAACAGCGAAGCTCATCTTATGCAGGGCACAAACATACTTATCTCTATGATAAGGGCAATGCAGAGGAAGTTCCCACGCTGGACAAAGGATCAAAAGCTGAAAGGTGGGATTTCTGCCTACAATGCTGGTGCTGGTAATGTTCGAACATATGACAGAATGGACATCGGCACCACTCACAATGACTACTCCAATGATGTGGTTGCACGAGCCAAGTATTACAAGAAACATGGATACTAG

>Finch_LygA

ATGATCCCTGCACTGCTGTTGCTGGGTCTTTCAGCCCTTGTTGCTCCATCCCTGAGTTACAGTTGCTATGGTGATATAAGTGCTCTTCAAGCCCCCACGATCTCCTGTACACCTGTAAGAGCCTCTGACTGTGGATATGCCATGATACGGAGGACGGCTGAGGCAGACCTGCCGCGCCTGAGGAGATACGAGATCCCAATTAAGAGAGTAGCCAGAAACCTGTGCTTGGAGCCAGCGCTCATCGGTGCCATCATGTCCCAGGAGAGCCGTGTGGGCCTGCTCCTGGACGACGGCTGGGACCGTGCGCGCCAGAAGTACGGCCTGATGCAGATCAGCAGGCAGCAAGTGCAACCTTCTGTGCCGTGGGATAGTGAAGAACACATAAATCAGTGCTCAAATATCCTGGTTCTTTCCATTAATGAAGTACGGGCAAGACATCCTACCTGGTCCTGGGACCGGCAGCTGAGAGGGGGAATCTGCACCTACCATGCAAAAATGGGCAACGTCCAGGTCTATGAGGCAGACCCATGCAGCAGCGACTACAACTACGTCAACAGCGTGATTAGGAGAGCCCAGTACTTTAAGAGAAATGGGTTCTAG

>Pigeon_LygA

ATGATCCCCACGCTGCTCTTGCTGGGCATTATGGCCCTTGTTGCTCCATCCATGAGTTACAGTTGCTACGGCGATGTAAGTACTCTTCAAGCCCCCATGGTCTCCTGTTCAGCCGTAAGAGCCCCAGACTGTGGACTTGCCATGATACGGAGAACTGCTGAGGCAGACATCGTACGCCTGAGGAAATATGAGATTCCGATTAAGAGAGTAGCCAGAAACCTGTGCTTGGACCCGGCGCTCATTGCTGCCATCATCTCGCAGGAGAGCCGTGCTGGTCTGCTCCTGGACAACGGCTGGGACCAGGAACGGCACAAGTACGGCTTGATGCAGCTTGGCAGGCAGCAACAGCAATCTTTCGGAATGTGGGATAGTGAAGAACACATAAATCAGGGCTCAACTATCCTGGTTCTTTCAATTAATGAAGTACGGGCAAGACATCCTACCTGGACCTGGGACCAGCAGCTGAGAGGGGGAATCTGCACCTACCGTGCAAAAATGGGCAACCTCCAGGTGTATGAGGACGACCCGTGTGACAGAGACAACTACTACGCCAACAGCGTGATTAGGCGAGCCCAGTACTTTAAGAGACATGGGTTCTAG

>Peregrine_Falcon_LygA

ATGATCCCCACACTGCTCTTGCTGTGCCTTATGGCCCTTGTTGCTCCATCCATGAGCTACAGTTGCTATGGTGACATAAGTGCTCTTCAAGCACCTCCAGTCTCCTGTACAGCCGTAAGAGCCTCAGACTGTGGACTTGCCACATTACGGAGGTCTGCTGAGGCAGACGTCATCCGCCTGAGGAGGTATGAGGTCCCAATTAAAAGAGTAGCCAGAAACCTGTGCTTGGACCCAGCGCTCATCGCTGCCCTCATCTCGCAGGAGAGCCGTGTTGGCCTGCTCCTGAACAACGGCTGGGACCAGGAACGGCAGAAGTATGGCTTGATGCAGCTTGACGGGCAGCAATACCACCCTTTTGGATTGTGGGATAGTGAAGAACACATAAATCAGTGCTCAACTATCCTGGTTCTTGCAATTAATGAAGTACGGGCAAGGCATCCTACCTGGACGTGGGACCAGCAGCTGAGAGGGGGAATCTGCACCTACCGTGCAAAAATGGGCAACATCCAGGTCTACGAGGAAGACCCATGCAACAGGGACAACTACTATGTCAACAGCGTGATTAGGCGAGCCCAGTATTTCAAAAGACACGGGTTCTAG

>Saker_falcon_LygA

ATGATCCCCACACTGCTCTTGCTGTGCCTTATGGCCCTTGTTGCTCCATCCATGAGCTACAGTTGCTATGGTGACATAAGTGCTCTTCAAGCGCCTCCAGTCTCCTGTACAGCCGTAAGAGCCTCAGACTGTGGACTTGCCACAATACGGAGGTCTGCTGAGGCAGACGTCATCCGCCTGAGGAGGTATGAGGTCCCAATTAAAAGAGTAGCCAGAAACCTGTGCTTGGACCCAGCGCTCATCGCTGCCCTCATCTCGCAGGAGAGCCGTGTTGGCCTGCTCCTGAACAACGGCTGGGACCAGGAACGGCAGAAGTATGGCTTGATGCAGCTTGACGGGCAGCAATACCACCCTTTTGGATTGTGGGATAGTGAAGAACACATAAATCAGTGCTCAACTATCCTGGTTCTTGCAATTAATGAAGTACGGGCAAGGCATCCTACCTGGACGTGGGACCAGCAGCTGAGAGGGGGAATCTGCACCTACCGTGCAAAAATGGGCAACATCCAGGTCTACGAGGAAGACCCATGCAACAGGGACAACTACTATGTCAACAGCGTGATTAGGCGAGCCCAGTATTTCAAAAGACACGGGTTCTAG

>Sparrow_LygA

ATGATCCCTGCACTGCTGCTGCTGGGTCTTTCGGCCCTTGTTGCTCCATCCCTGAGTTACAGTTGCTATGGTGATATAAGTGCTCTTCAAGCCCCCACGGTCTCCTGTACACCTGTAAGAGCCTCTGACTGTGGATATGCCATGATACGGAGGACGGCTGAGGCAGACCTGCCACGCCTGAGGAGATACGAGATCCCAATTAAGAGAGTAGCCAGAAACCTGTGCTTGGATCCAGCTCTCATCGGTGCCATCATGTCCCAGGAGAGCCGTGTGGGCCTGCTCCTGGACAACGGCTGGGACCGCGCGCGCCAGAAGTACGGCCTGATGCAGATCAGCAGGCAGCAAGTGCAACCTTCTGTGACGTGGGATAGTGAAGAACACATAAATCAGTGCTCAAATATCCTGGTTCTTTCCATTAATGAAGTACGGGCGAGACATCCTACCTGGTCCTGGGACCGGCAGCTGAGAGGGGGAATCTGCACCTACCATGCCAGAATGGGCAATGTCCAGGTCTACGAGGCAGACCCGTGCAGCAGCGACTACAACTACGTCAACAGCGTGATTAGGAGAGCCCAGTACTTTAAGAGAAATGGGTTCTAG

>Tit_LygA

ATGATCCCCACACTGCTGTTGCTGGGTCTTTCAGCTCTTGTTGCTCCCTCTGTGAGTTCCAGTTGCTATGGCGATATAAGAGCTGTACAAGTCCCCATGGTCTCCTGTACACGAGATCCTGCAAGATCCTCTGATTGTGGATATGCCATGATACAAAGGACTGCAGAGCCAGACCTCGTACGCCTGAGGAGATACGAGATCCCAATTAAGAGAGTAGCCAGAAATCTGTGCTTGGACCCAGCGCTCATCGGTGCCATCATGTCCCAGGACAGCCGTGTCGGCCTGCTCCTGGACAACGGCTGGGACCAGGGACGGCAGAAGTACGGCCTGATGCAGATCAGCAGGCAACTGCAACCTTATGCAGTGTGGGATAGTGAAGAACACATAAATCAAGGCTCAAATATGCTGGCTCTTTCCCTTAATGAAGTACGGGCAAGACATCCTACCTGGACTTGGGACCGGCAGCTGAGAGGGGGTATCTGCACCTACCATGCAAGAATGGGCAACCTCCCTGTCTACGAGGCAGACCCATGCAGCAGAGACTACAGCTATGCCAACAATGTGATTAGGCGAGCCCAGTACTTTAAGAGACATGGGTTCTAG

>Flycatcher_LygA

ATGATCCCTGCACTGCTGTTGCTGGGTCTTTCAGCCCTTGTTGCTCCATCCATGAGTTACAGTTGCTATGGTGATATAAGTGCTCTTCAAGCCCCTACCATCTCCTGTACACCTGTAAGAACCTCTGATTGTGGGTATGCCATGATAAGGAGAACTGCTGAGGCAGACCTCGTACGCCTGAGGAGATACGAGATCCCAATTAAGAGAGTAGCCAGAAACCTGTGCTTGGACCCAGCGCTCATCGGTGCCATCATGTCCCAGGAGAGCCGTGTTGGCCTGCTCCTGGACAACGGCTGGGACCGGGGACGGCAGAAGTACGGCCTGATGCAGATCAGCAGGCAGCAACTGCAGCCTTATGTGGCGTGGGATAGTGAAGAACACATAAATCAGTGCTCAAATATCCTGGTTCTTTCCATTAATGAAGTACGGGCAAGGCATCCTACCTGGACCTGGGACCGGCAGCTGAGAGGGGGAATCTGCACCTATCATGCAAAAATGGGCAACGTCCAGGTCTACGAGGCAGACCCATGCAGCAGAGACTACAACTACGTCAACGGCGTGATTAGGCGAGCCCAGTACTTTAAGAGAAACGGGTTCTAG

>Budgerigar_LygA

ATGATTTCTACACTGCTCTTGCTGGGCCTTATGGCCCTTGTTGCTCCATCCATGAGTTACAGTTGCTATGGTGATATAAGTGCTCTTCAAGCCCCACTGATCCCCTGTACAGCTGTAAGAGCCTCAGATTGTGGACTTGCCCTGGTACGGAGCTCTGCTGAGGCAGACATCATACGCCTGAGGAAATACGAGGTCCAGATTAAGAGAGTAGCCAGAAACCTGTGCCTGGAGCCTGCACTCATCGCTGGCATCATCTCGCAGGAAAGCCGTGTCGGCCTGCTCCTGGACAATGGCTGGGACCAGGAACGGCACAGGTACGGCTTGATGCAGCTTGGTGGGCATCAACAGCCTTTTGGACTGTGGGATAGTGAAGAACATATAAATCAGTGCTCAACTATCCTGGTTCTTTCAATTAATGAAGTACGGGCAAGACATCCTACCTGGACCTGGGACCAGCAGCTGAGAGGGGGAATCTGCACCTACCGTGCAAAAATGGGCAACTTCCAGGTCTACGAGGATGACCCCTGTGACAGAGACAGCTACTACGTCAACAGTGTGATTAGGAGAGCCCAGTACTTTAAGAGACACGGGTTCTAG

>Anole_lizard_LygC

ATGGCAGCTTGCGCTTATGGCAATATTATACATGTTGATACTACTGGAGCGTCCGCTGAAACTGGAAAACAAGAAGGCCTGAGTTATGGTGGGGTTCCAGCTTCAGAAAAAAATGCAGAGAGAGATCTAAAGAATCTGGAGAAATATAAGACAAAGATTATGAATGTCAGTAGAAAAACAGGTATTGATCCAGCTCTAATTGCTGCTATAATCTCCCGAGAATCTCATGCTGGAACTCTCCTGAAGAATGGCTGGGGTGACCATGGAAATGGATTTGGTTTGATGCAGGTTGACAAACGATTCCATAAACCCACAGGCACATGGGATAGTGAGGAACATATGACTCAGGCTACCAGCATTCTGTGTTCCATGATAAAGGACATTGAAAAAAAATTTCCGCAGTGGACTAAGGAACAACAGCTCAAAGGTGGAATTTCAGCCTACAATGCAGGTGTTAACAATGTCAAAACTTATGACCGAATGGATATAGGCACAACAAAGAATGATTATGCCAATGATGTTGTTGCAAGAGCTAAGTTTTACAAAAGAAATGGATACTAA

>Burmese_python_LygC

NCTCATTCTTCTTATGGTAACATAATGGCTGTGGATACCACTGGAGCTTTTCTTGAAACTGGAAAACAAGAAGGCTTGAGTTATGGGGGAGTTGCAGCTTCAGAAATAATTGCGAAGAGAGATCTAAACAATCTAGAGAGATATAAAACGAAGATTAAGCATGTCAGTGAAAAGACAAATATTGATGCAGCTGTGATTGCTGCTATAATGTCTCGAGAATCACATGCTGGAACTGTCCTAGAAAATGGTTGGGGTGACCATGGAAATGGATTTGGATTAATGCAGGTGGATAAAAGATACCATACACTAGTTGGCACCTGGGACAGTGAGGAACACATTAATCAGGCTACCCTCATTCTCTGTAGCATGATACAGGAGATTAAAAAAAAATTTCCATCATGGACAAAGGAACAGCAACTCAAAGGTGGAATTTCAGCCTACAATGCAGGACCTAAAAATGTCCAGAGCTACGATAGAATGGATATAGGCACTACAAAGAATGACTATGCCAATGATGTTGTTGCAAGAGCCAAGTTTTACAAAACAAGTGGATATTAA

>Painted_turtle_LygB3

ATGCTACTAACGCTGATGATTCTGGGCCTTGCTGCCCTCATTGGTACTTCTGAGAGTCAGACTGGATGCTATGGTGTCATAAACAAGGTTGATACCACTGGGGCTTCCTGTAGAACTGCAAAAGCAGAACGCTTACCCTACTGTGGAGTTCGTGCTTCAACAACGATTGCACTGAGAGACTTGCGTGCTATGAACAATTATAAAACCATCATTAAGAGCGCTGGAAGAAAAAAATGTGTGGATCCAGCTGTGATTGCTGGTATCATCTCTCGAGAGTCGCATGCTGGAAAGGCCCTAAAGGGTGGCTGGGGTGATCGTGGAAATGGATTTGGTTTGATGCAGGTTGATAAACGCTACCATAAATTGGTTGGACGATGGAATAGTGAGGCACATGTCCTTCAGGGGACAGGCATCCTTGTTAATATGATTAAGGGAATCCGGAAAAAGTTCCCCAGATGGACAAAGGAACAACAACTGAAAGGAGGGATTTCTGCCTACAATGCAGGACTAAGAAACGTCCAGTCCTATGATAAAATGGATATTGGCACAACCGGCAATGACTATGCCAATGATGTCGTTGCACGAGCCAAGTATTATAAGAGAAACGGATACTGA

>Painted_turtle_LygB1

ATGCTACTAACGCTGATGATCCTGGGCCTTGCTGCCCTTTTTGGTACTTCTGAGAGTCAGACTGGATGCTTTGGTAACATAAACAACGTTGATACCNACTGTAGAACTACAAAAGCAGAATGCTTACCATACTGTGGAGTTCCTGCATCAGAGAAGATTGCTGAGAGGGACTTAAATAATATGAACAAATATAAAGCCATCATTAAGAGCGCTGGCGGAAAAAAATGTGTAGATCCAGCTGTGATCGCTGGTATCATCTCTCGGGAGTCACATGCTGGAACTCTCCTAAAGGCTGGCTGGGACGATAACGGCAATGCATTTGGTTTGATGCGGGTTGATAAAAAACACCATAGTATTGTTGGANNCGGTGAAGATCATCTCCTTGATGCTACACAAATCCTTATTGGTATGATTAAGGGAATCCAGAAAAAATTCCCCAGATGGACAAAGGAACAACAGCTGAAAGGTGGGATTTCTGCCTATAATGCAGGACTAGGAAACGTCCAGACCTATCCAAACATGAATATTGGCACAACCCACAATGACTATGCCAATGATGTTGTTGCACGAGCCAAGTATTATAAGAGAAACGGATACTGA

>Chinese_softshell_turtle_LygB3

ATGGGTCTCACTGGTTCTTCTGAAAGTGAAATTGAATGCAATGGAAACATAAATGACGTTGATACCACTGGGGCGTCCAGTACAACTGCAAGACCAGAAGGTTTAAACTATTCTGGAGTCCGTGCTTCAGAGGAGATTGCTCGGAAGGACTTGGATAGAATGAAAGGATATAAACCCATGATTATTAGTGCTGCCAGAAAACACAATATGGATCCAGCTGTGATTGCTGGTATCATCTCCCGAGAGTCACATGCTGGACATGTCCTAGAGAATGGCTGGGGTGATCATGGAAATGCATTTGGTTTGATGCAGGTTGATAAACGCTACCATACCCCAGTCGGATCATGGAATAGCCAGGAACATCTCGATCAGGGGACAGGCATCCTTGCTGATATGATTAACACAATTGAAGATAAGTTCCCTGAATGGACCACGGAACAACAGCTGAAAGGTGGGATTTCTGCCTACAATACAGGACCAGGAAACGTCCATAATTATCCTGGAGTGGATAATGGCACAACTCACCAGGATTATGCCAATGATGTAGTTGCACGAGCCAAGTTTTATAAGAGAAATGGATATTGA

>Chinese_softshell_turtle_LygB1

ATGCTACTCACACTGATGATTCTGGGCCTCATTGGTACTTCTGAAAGTCAGACTGGATGCTATGGTGACATAAACAAAGTTGATACCACAGGGGCTTCCTGTAAAACTGCAAGAGCAGAAGGCCTAAGCTACTGTGGAATTAATGCTTCAGCAAAGATGGCGGAGAAAGACTTACCTACTATGAATAATTATAAAGCCATCATTAAGAACGCTAGCAGAGCAATTTGTATGGATCCAGCTGTGATCACTGGCATCATCTCCCGAGAGTCAAATGCTGGAACCCTCCTAAAGGATGGCTGGGATGAACTTGGAAATAAATTTGGTTTGATGCAGATTGATAAACACAAACATCAACTGTTTGGACCATGGAATAGCAAAGTGCACCTCATTCAGGGCACAGGCATCCTTGCTGATAATATTAAATTAATCCAGAAAAAATTCCCCAGATGGACCAAGGAACAACAGCTGAAAGGTGGAATTTCTGTCTATAATGCAGGACTAGAAAATGTCCAATCCTATGATAAAACAGATATTGGTACAACCAACAATGACTATGCCAACGATATAGTTGCACGAGCCAAGTATTTTAAGAGAAATGGATACTGA

>Chiese_softshell_turtle_LygB4

ATGGTACTCACGCTGATGATTCTGGGCCTTGCTGCCCTTTTTGGTACTTCTGCGAGTCAGACCAAATGCTATGGTGACATAACCAAGGTTGATACCATTGGGGCTTCCTGTAAAACTGCAAAAGCAGAAGGCTTAAGCTTCTGTGGAGTTCAAGCGTCAGAAAAGATCGCTGAGAAGGACTTAAATAATATGAACAAATACAAAGCCATCATTAAGGGCACTGGCCTAAAAACATGTGTGGATCCAGCTCTGATTGCTGGTATTATCTCTCGAGAGTCTCATGCTGGAGCTTCCCTGAAGAATGGCTGGGGTGATAATGGAAATGCTTTTGGTTTGATGCAGGTTGATAAAAACAATCATAAAATTGTTGGACGGTGGAATGATGAAGACCACCTGCTTGATGCAACACGAATCTTTATTGGCATGATAAATGGAATCAAGGCAAAGTTCCCCAAATGGACAATAGAACAACAGCTGAAAGGTGCGGTTTCTGCCTACAATGGAGGAATAGGAAATGTCCGAAGTTATGATAATATGGATATTGGCACACCTGGCAATGACTATGCCAGCGATGCCACTGCACGAGCCAAGTTTTTCAAAAAACAGGGTTACTGA

>Chinese_softshell_turtle_LygA

ATGCTACTAACACTGATGTTTCTGGGTCTTGCCGCCCTTGTTGCTCCTTCTGCAAGTTATGGATGTTATGGTGATTTACATACTATGGAAACCCCTGTGATTTCCTGCAGGCCTGTACAAACCCCAACTTGTGGTGTGCTTACGGCACAAAAGATTGCTGAAATAGATATTGTACGTCTAAGGGCCTATCAAGCCCCTATTATGAGGGTTGCCAGAAAGACGTGCTTAGATCCAGCACTGGTTGGTGCAATCATATCTGTGGAGAGTCGTGTTGGAGCTGCTCTTCATGATGGCTGGAACCGTGAAAGAAACCGATTTGGCTTGATGCAGATTCCTGTAAACTACAATTTGGTTCCTGTAGCCTGGAACAGTGAAGATCATATAGCTCAAGGTGCAAATATTCTAGTTACTGGGCTAGAGGATATAAAGAGAAGGTTCCCTACCTGGCAATGGCAACAACACCTCAGAGGTGGGATTTGTTACTATTATAACAAGGTTAGGAACATCCAGGTCAATGACGGAATGGACATTTGCAATTCGAACGATGATTACGTTAATAATGTTATTATTCGAGCCAAGACTCTGCAGAATAATGGATTTAGAATTTTAGAGTAA

>Chinese_alligator_LygB2

ATGTATCTGACACTAATCATTTTGGGCCTAGCTGCCCTTATTGACACTTCCAATCAGTCTGGATGCTATGGTGATATAAACAGAGTTGATAGCACTGGGGCATCTTGTCAAACTGCAAAACCAGAACGCTTAAAATACTGCGGAGTTCCAGCTTCTCAAAAGATTGCTGAGGGGGATCTAACCAGAATGAATAAATATAAAGACATCATTAAAAGTGCTGGCAGAAAACAGTGTGTGGATCCAGCTGTGATTGCTGGCATCATCTCTAGAGAGTCACATGCAGGAGCTGCCCTAACCGCTGATGGCTGGGGTGACGGGGGAAATGCCTTTGGTTTGATGCAAGTTGATAAGAGGTATCATACACCCGTTGGGAAATGGAACTCTGAGGCTCATCTCATCCAGGGCACACAAATCCTTGTGGGTATGATTAAGGAAATCCAGAGAAAGTTCCCAAGGTGGACTAAAGAACAACAGCTGAAAGGTGGGATTTCTGCCTACAATGCAGGACCTCGAAATGTACAGACCTATCAAGGAATGGATATTGGCACAACCCACAATGATTATGCCAATGATGTAGTTGCACGAGCCCAGTTTTACAAGAAAAATGGATACTAA

>Chinese_alligator_LygB1

ATGCATCTGCCACTGATCATTCTGGGCCTAGCTGCCCTTTTTGACACTTCCAATCAGTCTGGATGCTATGGTGATATAAACAGAGTTGATACCACTGGGGCGTCTAGTCAAACTGCAAGACCAGAAGGTTTAAACTATGCTGGAGTCCGGGCTTCTGAAACGATTGCTGCAAAGGACCTAATCAGAATGAATAAATACAAAGATTTAATTCAAAGAGCTGGCAGCAACCAGTGTGTGGATCCAGCTGTGATTGCTGGCATCATCTCTAGAGAGTCACATGCAGGAGCTGCCCTAACCGCTGATGGCTGGGGTGACAGGAGAAATGCCTTCGGTTTGATGCAAGTTGATAAGAGGTATCATAAACTCGTTGGGGAATGGAACTCTGAGGCTCATCTCATCCAGGGCACACAAATCCTTGTCGGTATGATTAAGGCAATCCAGAGAAAGTTCCCAAGGTGGACAAAGGAACAACAGCTGAAAGGTGGGATTTCTGCCTACAATGCAGGACCTGGAAATGTCCGGACCTATGAAAAAATGGATATTGGCACAACCCACAATGATTATGCCAATGATGTAGTTGCACGAGCCAAGTTTTACCAGAAAAAAGGATTCTAA

>Chinese_alligator_LygB3

ATGCATCTGCCACTGATCATTCTGGGCCTAACTGCCTTTATTGACATTTCCTTCAGTCAGTCTATATGCTATCGCAATATAAATGGAGTTGATACCACTGGGGCATCTTGTCAAACTGCAAGATCAGAAGGCTTAATATATTGTGGAGTTCCAGCTTCTGAAAAGATTGCTGAGGGGGATCTAACCAGAATGAATAAATATAAAGGCATCATTAAAAGTGCTGGCAGAAAACTGTGCGTGGATCCAGCTGTGATTGCTGGCATCGTCTCTAGAGAGTCACATGCAGGAGCTGCCCTAACCCACGATGGCTGGGATGAAAGGAGAAATGGTTTTGGTTTGATGCAAATTGATAGACGGTCTTATCCTCAACTTACTCGACCATGGAACTCCGAAGCTCATCTCATTCAAGGCACACAAATCCTTGTGAAAATGATTACGGCAATCCAGAGAAAGTTCCCAAGGTGGACTAAAGAACAACAGCTGAAAGGTGGGATTTCTGCCTACAATGCAGGACCTCGAAATGTCCAGACTTATGCTGATATGGATATTGGCACAACCCACAATGATTATGCCAATGATGTAGTTGCACGAGCCCAGTTTTACAAGAAAAATGGATACTAA

>Chinese_alligator_LygC

ATGTGCAGATTTAGGGGGCATATCTCCCATAATGAGTTGAAGGTTTTCTGGAAGCATGCAAACCTTTCTGAACACTACGGTAAATTAATGGATGTCGATACAACTGGGGCTTCCATTGAAACTGGAAAACAAGAAGGTCTAAGCTATGGAGGAGTGACTGCTTCAGAAAAGATTGCTGAAAGAGATTTAAAGAATCTGCAAAAATATGAAACCAAGATTAAGAACGTTGGTAAAAAACTGGGTGTAGATCCAGCTCTGATTGCTGCTATCATCTCCCGAGAGTCTCATGGTGGAATAGTCCTGAAGGACGGCTGGGGTGACAGAGGAAACGGATTTGGTTTAATGCAGGTTGATAAACGACACCATGAAATTGTTGGTACGTGGGACAGTGAAGAGCATATTACGCAAGGTACAGAGATTTTGTGTGGGATGATAAAAGACATCCAGAAGAAATTCCCACAATGGACAAGAGAACAACAGCTAAAAGGTGGGATTTCTGCCTATAATGCAGGGCCTAAGAACATTCAGAGCTATGAGAGAATGGACATTGGCACAACTAAGAATGACTATGCCAATGACGTTGTTGCAAGAGCCAAATTTTATAAGAGAAATGGATACTGA

>Chinese_alligator_LygA

ATGTTTCTTGCACTGTTGATTCTGGGCCTTTCTGCCCTTATTGCTCCTTCTGCCAGTCAAAGTTGCTATGGTGATATAACTAGCCTTCAAACTCCGGTAATATCCTGTGGACCGGTAAAAACTATAGGCTGTGGTGTTTCTGCTTCGGAAAAAATTGCTGAAATTGACATAATACATCTAAGGTCATATAGAACCATTATTAGGAGAGTTGCCAAAAGCCTGTGTTTAGAACCATCATTGATTGCTGCAATCATCTCTGTAGGGAGTCGTGTCGGTCATCTACTAGAGAATGGCTGGGACCATCAGAGAGTTAGGTTTGGCTTGATGCAGATTGACAGAAGATACTATCCCATTAGGGGAACATGGAACAGTGAAGAACATATAATTCAAGGATCAACTATTTTAACCATTGCACTTAAAACTGTGCGGAAAACATACCCTAGGTGGACCTGGGCACAACAGCTAAGAGGGGCACTTTGTGCCTATCATTCGAGGGACTGGAAAATCCAGGTCTATGATGGAAGAGACTATTGCAGTGGAGGCAATGATTATGTTAATAATGTTATCATTCGAGCAAAGTATTTCAAGAGAATCGGATTTTAA

>American_alligator_LygB2

ATGTATCTGACACTAATCATTTTGGGCCTAGCTGCCCTTATTGACACTTCCAATCAGTCTGGATGCTATGGTGATATAAACAGAGTTGATAGCACTGGGGCATCTTGTCAAACTGCAAAAGCAGAAGGCTTAAAATACTGCGGAGTTCCAGCTTCTCAAAAGATTGCTGAGAAGGATCTAACCAGAATGAATAAATATAAAGACATCATTAAAAGTGCTGGCAGAAAACAGTGTGTGGATCCAGCTGTGATTGCTGGCATCATCTCTAGAGAGTCACATGCAGGAGCTGCCCTAGATGCTAATGGCTGGGGTGACGGGGGAAATGCCTTTGGTTTGATGCAAGTTGATAAGAGGTATCATAAACCCACTGGGGCATGGAACTCTGAGGCTCATCTCATCCAGGGCACACAAATCCTTGTGGGTATGATTAAGGAAATCCAGAGAAAGTTCCCAAGGTGGACTAAAGAACAACAGCTGAAAGGTGGGATTTCTGCCTACAATGCAGGACCTCGAAATGTACAGACCTATCAAGGAATGGATATTGGCACAACCCACAATGATTATGCCAATGATGTAGCTGAACGAGCCAAGTTTTACAAGAAAAATGGATACTAA

>American_alligator_LygB1

ATGCATCTGCCACTGATCATTCTGGGCCTAGCTGCCCTTATTGACACTTCCAATCAGTCTGGATGCTATGGTGATATAAACAGAGTTGATACCACTGGGGCGTCTAGTCAAACTGCAAGACCAGAAGGTTTAAGCTATGCTGGAGTCCGGGCTTCTGAAACGATTGCTGCAAAGGACCTAATCAGAATGAATAACTATAAAGGTTTAATTCAAAGAGCTGGCAGCAACCAGTGTGTGGATCCAGCTGTGATTGCTGGCATCATCTCTAGAGAGTCACATGCAGGAGCTGCCCTAACCGCTGATGGCTGGGGTGACGGGGGAAATGCCTTCGGTTTGATGCAAGTTGATAAGAGGTATCATAAACTCGTTGGGAAATGGAACTCTGAGGCTCATCTCATCCAGGGCACACAAATCCTTGTGGGTATGATTAAGGCAATCCAGAGAAAGTTCCCAAGGTGGACAAAGGAACAACAGCTGAAAGGTGGGATTTCTGCCTACAATGCAGGACCTGGAAATGTCCGGACCTATGAAAAAATGGATATTGGCACAACCCACAATGATTATGCCAATGATGTAGTTGCACGAGCCAAGTTTTACCAGAAAAAAGGATTCTAA

>American_alligator_LygB3

ATGCATCTGCCACTGATCATTCTGGGCCTAACTGCCTTTATTGACACTTCCTTCAGTCAGTCTAGATGCTATCGCAATATAAATGGAGTTGATACCACTGGGGCGTCTTGTCAAACTGCAAGATCAGAAGGATTAATATACTGTGGAGTTCCAGCTTCTCAAAAGATTGCTGAGAGGGACCTAACCAGAATGAATAAATATAAAGGCATCATTAAAAGTGCTGGCAGAAAACTGTGCGTGGATCCAGCTGTGATTGCTGGCATCATCTCTAGAGAGTCACATGCAGGAGCTGCCCTAACCCACGATGGCTGGGATGAAAGGAGAAATGGTTTTGGTTTGATGCAAATTGATAGACGGTCTTATCCTCAACTTACTCGACCATGGAACTCCGAAGCTCATCTCATTCAAGGCACACAAATCCTTGTGAAAATGATTACGACAATCCAGAGAAAGTTCCCAAGGTGGACTAAAGAACAACAGCTGAAAGGTGGGATTTCTGCCTACAATGCAGGACCTCGAAATGTCCAGACTTATGCTGATATGGATATTGGCACAACCCACAATGATTATGCCAATGATGTAGTTGCACGAGCCCAGTTTTACAAGAAAAATGGATACTAA

>American_alligator_LygA

ATGTTTCTTGCACTGTTGATTCTGGGCCTTTCTGCCCTTATTGCTCCTTCTGCCAGTCAAAGTTGCTATGGTGATATAACTAGCCTTCAAACTCCGGTAATATCCTGTGGACCGGTAAAAACTGTAGGCTGTGGTGTTTCTGCTTCGGAAAAAATTGCTGAAATTGACATAATACATCTAAGGTCATATAGAACCATTATTAGGAGAGTTGCCAAAAGCCTGTGTTTAGAACCATCATTGATTGCTGCAATCATCTCTGTAGGGAGTCGTGTCGGTCATCTACTAGAGAATGGCTGGGACCATCAGAGAGTTAGGTTTGGCTTGATGCAGATTGACAGAAGATACTATCCCATTAGGGGAACATGGAACAGTGAAGAACATATAATTCAAGGATCAACTATTTTAACCATTGCACTTAAAACTGTGCGGAAAACATACCCTAGGTGGACCTGGGCACAACAGCTAAGAGGGGCGCTTTGTGCCTATCATTCGAGGGACTGGAAAATCCAGGTCTATGATGGAAGAGACTATTGCAGTGGAGGCAATGATTATGTTAATAATGTTATCATTCGAGCAAAGTATTTCAAGAGAATCGGATTTTAA

>Xenopus_laevis_Lyg

ATGTTCTCTGAGATTTTCCCATTTGTCCTCCTGCTATGCAGTGTAAGTGCATCAGGACGATTCGGCGACATAAACAAAGTTCAGACCTCAGGAGCTTCATGCCGAACAGCTAAACAAGACAAGCTGACAATCTGTGGAGTCCAGGCTTCTGAGACTATGGCGCAGACAGACCTAACAAGGATGAACAGATACAAGTCTATAATTCAGTCAGTGTCTCAGAAGAAAGGCATGGATGCAGCTTTAATTGCTGGAATCATATCTCGGGAATCACGCGCCGGAAATGTTCTTGTTAATGGGTGGGGTGACCATGGCAATGCTTTTGGTCTCATGCAGGTTGATAAAAGGTTCCATACAGTTACTGGGGCCTGGAACAGCGAAGAGCATGTTACACAAGGAACGGACATTTTGATCGGCATGTTTGACTCCATCAGAAGAAAATTTCCTACATGGTCAACAGAACAGCATCTGAAAGGTGCTATTGCAGCATATAATGCTGGTCCAGGAAATGTCATCAGCCTTGATGTGGACACTCGCACTACAGGAAAAGACTACGCCAATGATGTTGTAGCTCGAGCAAAATTTTATAAAAAGAGGGGATACTAA

>Xenopus_tropicalis_Lyg2

ATGTTCTCTGAGATTTTCCCATTTGTCGTTCTGCTATGCAGTGTAAGTGCATCAGGACAATATGGTGATATCAACAAAGTTCCGACCTCAGGAGCTTCATGCCGGACAGCAAAACAAGACAAGCTGACAGTGTGTGGTGTCCAGGCTTCTGAGCGTATGGCACAGACAGACCTAACAAGGATGAACAGATACAGGTCTATAATTGAATCTGTGTCTCGGAAGATGGGCATGGATGCAGCTTTAATTGCTGGAATCATATCTCGGGAATCTCGAGCTGGAAATGTTCTTATAAACGGGTGGGGTGACAATGGCAATGCCTTCGGTCTCATGCAGGTTGATAAAAGGTTCCATAAAATTTCTGGGGCCTGGAACAGCGAAGAGCATGTTACACAAGGGACCGAGATTCTGATCGGCATGTTTGCATCCATCAAAAGAAAATTTCCTCAATGGTCAACAGAGCAGCACCTGAAAGGTGCTGTTGCAGCATATAATGCTGGTCCAGGAAATGTAATCAGCAATGATGTAGATGTTCGCACTACAGGGAAAGACTACGCCAATGATGTTTTAGCTCGAGCAAAGTTTTATAAAGGGAGGGGATACTAA

>Axolotl_Lyg

ATGCTGGCTCTACTCAGCGCTCTGTGTCTAGCTGGGTCCATTGGTCTGTCCGCTGCATCTGGCTGCTATGGCAATATAATGGATGTCCCCACCACTGGGGCTAGTTGCCTCACTGCAAGCCAAGACAATCTACCATACTGCGGCGTTGCAGCGTCCCAACAGATGGCAGCAACTGACCTCCCTGATATGAACCAGTACAAAGAAAAGATTTTGGCTGTTGCGCAGAACTTGTGCATGGATGGAGCAGTGATTGCCGGGATCATTTCCCGTGAGTCGCGTGCGGGGGCGGTCCTGCAGAATGGATGGGGCGACAACGGACATGCGTTTGGTCTAATGCAGATCGACATTCGTTGGCACTCAATTGAGGGGGCCTGGAACAGTCAAGAGAACATCAATGAGGGCACAGGGATCCTGATCAACATGATTGTAGCAATTTCAGACAAGTTTCCAAGCTGGTCTGTGAATGACAACCTGAAAGGAGGAATTGCTGCATATAATGCTGGTCCTGGGAACATCTACAGCTACTCTCAAGTAGATCAGTACACAACGGACGGGGACTACTCCAATGATGTGGTTGCCAGAGCCCAGTATTACAAGACTCAGGGTTACTAG

>Coelocanth_Lyg1

ATGGCTTGTATTTATGGCAATGTTATGAACATTGACACTACTGGAGCATCCCAGGCAACAGCAAAGCAGGACAAACTAAAGGAGGATGGAGTGCCAGCCTCCCACAAGATGGCTGAAAACGATTCTGGTAGGATGTGCAAATATAAAACAAGAATTCTTGAAGTGGGACGTGCTAAGCAAATGGATCCAGCTGTCATTGCAGCAATCATTTCCCGTGAATCTCGTGCTGGAGCTGCCTTACATGATGGATGGGGAGACCATGGCAATGGTTTTGGCTTAATGCAGGTTGATAAACGCTATCACACCCCTGTGGGAGCCTGGGACAGTGAGGAGCATATTTCTCAGGGAACTCAGATATTGATTGATATGATTACCAGTATCCAGAAGAAATTTCCAGGGTGGACCCTGGACCAGCAGATGAAAGGTGGGATCTCAGCCTATAATGCTGGTGTTGGCAATGTCCGAACCTATAACAAGATGGATGTTGGTACCACTGGAGGAGATTATGCAAATGATGTTGTTGCAAGAGCTCAGTGGTATAAGAGCAATGGATATTAA

>Coelocanth_Lyg2

NCAAATAATTATGGCAATGTTATGAACATTGACACTACTGGGGCATCCAAGGCAACAGCAAAGCAGGACAAACTAAAGGAGGGTGGAAAGTTAGCGTCCCGCAAGATGGCTGAAACCGATTCTGCACGGATGGAAAAGTACAAAGATATAATTGTTAAAGTGGGAAAAGCTAAAAATATTGATCCAGCTGTCATTGCAGCAATCATTTCCCGCGAAACTCGTGCTGGAGGAGTTCGCTTAACTAAAGGATGGGGAGACCATGGCAATGGTTTTGGCTTAATGCAGGTTGACAAACGCTCTCACACCCCTGTGGGAGCCTGGGACAGTGAGGAGCATATTTCTCAGGGAACTCAGATATTGATTGATATGATTACCAGTATCCGGAAGAACTTTCCAAGGTGGACCCTAGACCAGCAGTTGAAAGGTGGGATCTCAGCCTATAATGCTGGTGTTGGCAATGTCCGAACCTATAACAAGATGGATGTTGGTACCACTGGTGGAGATTATGCAAATGATGTTGTTGCAAGAGCTCAGTGGTACAAAGACAATGGATATTAA

>Japanese_Flounder_LygF1

ATGTCTTATGGACAGATACGTTTGGTTGAGACCAGTGGAGCTTCAGGGGCAACTTCTCAGCAGGACAACCTGGGATACTCAGGTGTAAAAGCATCACACAAAATGGCAGAGATAGACAGTGGGAGAATGAGTAAATACAAGAGCAAAATCAATAAAGTTGGACAGAGCTACGGAATCGAACCGGCTCTCATTGCTGCCATCATCTCGAGAGAGTCCAGGGCTGGAAATCAACTGAAAGACGGCTGGGGAGACTGGAACCCACAGAGGCAGGCGTACAACGCCTGGGGACTGATGCAGGTTGATGTTAATCCCAACGGTGGTGGACACACTGCTGTAGGTGGATGGGACAGTGAGGATCACCTCCGCCAAGCCACTGGGATCTTGGTTACATTTATTGAGCGAATCAGAACCAAGTTTCCTGGCTGGAGCAAAGAGAAGCAGCTGAAAGGAGGGATAGCAGCCTACAACATGGGGGATAAAAATGTGCATTCCTATGAGGGAGTGGATGAAAACACAACAGGACGAGACTACTCCAATGATGTCACAGCCAGAGCTCAGTGGTACAGAGACAATGGGTACAGCGGCTGA

>Turbot_LygF1

ATGGGTTATGCAAATATCAAGGACGTTCAAACCACTGGAGCTTCCTGGAAAACGGGCCAGCAGGACAAACTGGGATACTCAGGTGTGGAGGCATCACACACTATGGCAGAGACTGACAGTGGCAGAATGAGTAGGTACAAGTCCAAGATCTTCAATGTGGGACAAAAGTGTGGAATCGACCCGGCTCTCATTGCTGCCATCATCTCAAGAGAGTCCAGGGCTGGAAATGTTCTGCATGACGGCTGGGGAGACTGGAACCCACACAGGAACGCGTACAACGCCTGGGGACTGATGCAGGTTGATGTTAATCCGAGTGGAGGTGGACACACTGCTGAAGGTGCATGGGACAGTGAGGTACACCTCTGCCAGGCCACCGGGATCTTGGTTGGTTTCATCGGCCGAATCCGCAATAAATTTCCTGGCTGGAGCGGAGAGCAGCACCTGAAAGGCGGGATAGCGGCCTACAACATGGGCGATGGAAATGTCCATTCCTACGCTGAAGTGGATGCGAACACAACAGGTGGAGACTACTCCAATGATGTTGTTGCCAGAGCTCAGTGGTACAAAAGGAATGGGTTTTAA

>Brill_LygF1

ATGGGTTATGCAAATATCAAGGACGTTCAAACCACCGGAGCCTCCTGGCAAACGGCCAAGCAGGACAAACTGGGATACTCAGGTGTGGAGGCATCGCACACTATGGCAGAGACTGACAGTGGCAGAATGAGTAAGTACAAGTCCAAGATCTTCAATGTGGGACAGACGTGTGGAATCGACCCGGCTCTCATTGCTGCCATCATCTCCAGAGAGTCCAGGGCTGGAAATGCTCTGCATGACGGCTGGGGAGACTGGAACCCACACAGGAATGCGTACAATGCCTGGGGACTGATGCAGGTTGATGTGAATCCGAGTGGAGGTGGACACACTGCTAAAGGTGCATGGGACAGCGAGGAGCACCTCTGCCAAGGCACTGGGATCTTGGTTCATTTCATCGGCCGAATCCGCAATAAATTTCCTGGCTGGAGTGGAGAGCAGCACCTGAAAGGCGGGATAGCGGCCTACAACATGGGCGATGGAAATGTCCATTCCTACGCTGAAGTGGATGCGAACACAACAGGTGGAGACTACTCCAATGATGTTGTTGCCAGAGCTCAGTGGTACAAAAGGAATGGGTTTTAA

>Mandarin_fish_LygF1

ATGGGTTATGGAAACATCATGAGGCTTGAAACTACTGGAGCTTCATGGGAAACAGCTCAGCAGGACAGTCTGGCATACTCAGGTGAGAGGGCATCACACACCATGGCAAAGACTGATGCGGGCAGAATGGAAAAGTACAGGTCTAAAATCAACAGTGTGGGAGCTAAATATGGAATCGATCCAGCTCTAATCGCTGCCATCATCTCCAGAGAGTCCAGGGCCGGAAATGCCCTACATGATGGCTGGGGAGACTATGACTCAAAGAGAGGAGCGTATAACGGCTGGGGACTGATGCAGGTTGATGTTAATCCAAACGGAGGTGGACACACTGCACAGGGCGCATGGGACAGTGAGGAACACCTCCGCCAAGGCACCGAGATCTTGGTTCATTTTATCAATCGGATCCGCAACAAATTTCCTGGCTGGAGCACGGAGCAGCAGCTGAAAGGAGGGATAGCAGCTTACAATATGGGGGATGGAAACGTCCATTCCTATGAAAACGTGGATGAGAACACAACAGGTAAAGACTACTCCAATGACGTCGTTGCCAGAGCTCAGTGGTACAAAAACAACGAAGGCTTTTAA

>Knifejaw_LygF1a

ATGAGTTACGGAAACATCATGGATGTTGAAACTAGTGGTGCCTCAGAGCGAACAGCTAAGCAGGACAAGCTGGGATACACAGGTGTGAGGGCATCACACACCATGGCAGAGACTGATGCTGGCAGAATGGAAAAGTACAGGTCTAAGATCAACACAGTGGGAGGTAAATATGGAATCGATCCGGCTCTCATTGCTGCCATCATCTCCAGAGAGTCCAGGGCTGGAAATACACTAAAGAATGGCTGGGGAGACTGGAACCCAAAAAGAGGAGCGTATAATGCCTGGGGACTGATGCAGGTTGACGTTAATCCAAGCGGAGGTGGACACACCGCACGGGGAGAATGGGACAGTGAGGAACACCTCTGCCAAGGCACCGAGATCTTGGTTGATTTCATCAAACGTATCCGCAACAAATTTCCTGGGTGGAGCTCGGAGCAGCAGCTGAAAGGAGGGATAGCAGCCTACAACCTGGGGGATGGAAACGTCCGTACCTATGAAAACGTGGATGGCGGCTCGACAGGTGGAGACTACTCCAATGATGTTGTTGCCAGAGCTCAGTGGTACAAAAACAACAAAGGCTTTTAA

>Seabass_LygF1

ATGGCTTATGGAAATATCATGAGAGTTGAAACTACCGGTGCTTCATGGCAAACATCTCAGCAGGACAGTCTGGGATACTCAGGTGTGAAGGCATCACACACCATGGCACAGACTGATGCAGGCAGAATGGAAAAGTACAGGTCTAAAATCAACAAAGTAGGAGGAAGTTGTGGAATTGATCCCGCTCTCATTGCTGCCATCATTTCCAGAGAATCCAGGGCTGGAAATGCACTCACTAATGGCTGGGGAGATGGGGGTAATGCCTGGGGACTGATGCAGGTTGATGTTAATCCAAATGGAGGTGGACACACTGCACAGGGTGCGTGGGACAGTGAGGAACACCTCAGGCAAGCGACCGGGATCTTGGTTAATTTTATCAAACGGATCCGCAACAAATTTCCTGGCTGGAACACAGAACAGCAGCTTAAAGGAGGGATCGCAGCCTACAATATGGGGGATGGAAATGTCCATTCCTATGAACGTGTGGATGGCTGCACAACAGGTGGAGACTACTCCAATGATGTTGTTGCCAGAGCTCAGTGGTACAAAACTAATAAAGGCTTTTAA

>Knifejaw_LygF1c

ATGGGTTACGGAAACATCATGATGGTTGAAACTACTGGTGCCTCATGGCAAACAGCTCAGCAGGACAGGCTGGGATACTCAGGTGTGAGGGCATCACACACTATGGCAGAGACCGATGCTGGCAGAATGGAAAAGTACAGGTCTAAAGTCAACACAGTGGGAGGTAAATATGGAATCACTCCGGCTCTCATTGCCGCCATCATCTCCAGAGAGTCCAGGGCTGGAAATACACTAGAGAATGGCTGGGGAGATTCACATAACGCCTGGGGACTGATGCAGGTTGATGTTAATCCACACGGAGGTGGACACACTGCACGGGGAGCATGGGACAGTGAGGAACACCTCTGCCAAGCCACCGAGATCTTGGTTTATTTTATCAAACGGATCCGCAACAAATTTCCTGGCTGGAGCTCGGAGCAGCAGCTGAAAGGAGGGATAGCAGCCTACAACATGGGGGACGGAAACGTCCATTCCTATGAAAACGTGGACGGTAGCACAACAGGTGGAGACTACTCCAATGATGTTGTTGCCAGAGCTCAGTGGTACCAAACCAACGGAGGCTTTTAA

>Croaker_LygF1

ATGGGTTATGGAAACATTATGAGGGTTCAAACTACCGGTGCATCAGAGAAAACATCTCAGCAGGACAAACTGGGATACTCAGGTGTGAAGGCATCACAAGCAATGGCAGAATTAGATGCAGGCCGAATGGAAAAGTACAGATCTAAAATCAACAGTGTTGGACGTAGATATGATATCGATCCAGCTCTCATCGCTGCAATCATCTCCAGAGAATCTAGGGCTGGAAATGCACTAACTAATGGATGGGGAGACTATAGCCCAGCGAGAGGACAATACAACGCCTGGGGACTGATGCAGGTTGATGTCAATCCGCAGGGAGGTGGACACACTGCAAAGGGCGCGTGGGACAGTGAGGAACACCTCTGCCAAGCTACTGGGATCTTGGTTCATTTCATCAAAGTGATTCGCAACAAATTTCCTGGCTGGAGCACAGAGGAGCAGCTGAAAGGAGGGATAGCAGCATACAATATGGGGGATGGAAGTGTCGAGGACAGAGATGTGGATAAAAACACAACAGGTAGCGACTACTCCAATGATGTTGTTGCCAGAGCTCAGTGGTACAAAAACAATAAAAACTATTAA

>Grouper_LygF1

ATGGGTTATGGAAACATCATGAATGTTGAAACTACTGGTGCATCATGGCAAACGGCTCAGCAGGACAAGCTGGGATACTCAGGTGTGAGGGCATCACACACCATGGCAAACACTGACTCAGGCAGAATGGAGAGGTACAGGTCTAAAATCAACTCCGTGGGAGCAAAATACGGAATCGATCCAGCTCTGATTGCCGCCATCATCTCCGAAGAGTCCAGGGCTGGAAATGTATTACATGATGGCTGGGGAGACTATGACTCAAACAGAGGAGCGTACAACGCCTGGGGACTGATGCAGGTTGATGTTAATCCAAATGGAGGTGGACACACTGCGCGGGGTGCATGGGACAGTGAGGAACACCTCTCTCAAGGCGCAGAGATCTTGGTTTATTTTATTGGACGCATCCGCAACAAGTTTCCTGGCTGGAACACGGAGCAGCAGCTGAAAGGAGGAATAGCAGCCTACAATATGGGGGATGGGAACGTCCACTCTTATGATAATGTGGATGGCAGAACAACAGGTGGAGACTACTCCAATGATGTTGTTGCCAGAGCTCAGTGGTACAAAACCCAGAAAGGCTTCTAA

>Senegalese_sole_LygF1a

ATGAGTTATGGAAGCATCGTGGATATTACAGCCTCTGGAGCGTCATGGCAAACTGCTCAGCAGGACAAGTTGGGATGCCAAGGGGTGGATGCATCACGCACCATGGCGAAGACTGACAGTGAGAGAATGAGGAAGTACAAGACAAAAATTGAAAATGTGGGACGTAAATATGGGATAGAGTCTGCTCTCATTGCTGGCATCATCTCCAGAGAGTCCAGGGCCGGCAATGTATTACATAATGGCTGGGGAGACTGGAATCCAAGCAGAGGAGCGTATAACGCCTGGGGGCTGATGCAGGTTGATGTCAATCCAAATGGAGGTGGACACACTGCTCGCGGTGAATGGGACAGCGAGGAACACCTCTGCCAAGGCGCTGAGATCTTGGTTTATTTCATTGGCCGAATCCGTAATAAATTTTCAGGCTGGAGCAGAGAGCAGCAGCTGAAAGGAGCGATTGCAGCCTACAACATGGGGGATGGAAATGTCCACTCCTATGATAAAGTGGATGAAAAAACAACAGGGAAAGACTACTCCAATGATGTTGTTGCCAGAGCTCAGTGGTACAAAGACAATGGCTACAACAGCTGA

>Starry_flounder_LygF1

ATGTGCAGGTTATGGAAACATCCGATCGTTGAGACCAGTGGAGCTTCATGGCAAACTGCTCAGCAGGACAGCCTGGGATATTCAGGTGTAAATGCATCACACACAATGGCACAGACGGACATGGGCAGAATGAGTCAGTTCAAGTCCAAAATCATGAGAGTCGGTCAGAGACACGGAGTCGACCCTGCTCTCATTGCTGCCATCATCTCGAGAGAGTCTAGGGCCGGAAATGCACTTCAAGGCGACTGGAGAGACTTTCACAACGCTTGGGGACTGATGCAGCTTCATGTTAATCCCAATGGAGGTGGACACACTGCTAAAGGTGGATGGGACAGTGAGGAACACCTCAACCAAGGCACTGAGATCTTGGTTAATTTTATTAACCGAATCAGCAATAAGTTTCCTGGCTGGAGCAGAGAGCAGCAGCTGAGAGGAGGGATAGCAGCCTACAACATGGGGGATGGGAATGTGCATTCCTATAGTGGAGTAGATCAACACACAACCGGTGGAGACTACTCCAATGACGTCACAGCCAGAGCTCAGTGGTACAAAAACAATGGGTACTGA

>Tongue_sole_LygF1

ATGGGAGGACTTTTTTCATCTTCTGCTTCCACGACTTCCTCGACTTCCTCCTCCTCTTCAAATTCCTCTGGAAACATAAACAGGATACCTACTAGTGGAGCTTCCTGGAAGACTGCTCAACAGGACAAACTGGATTATAAGGGTGCAAAAGCTTCACAGACTCTGGCTGAGACCGACAGTGATAGGATGAAGAAGTACAGAGACAAAATCATCAGAGTTGCCAATGAGACCGGAATCCAACCGTGTCTTATTGCTGCCATCATCTCCAGAGAATCCAGAGCTGGAAAAGCTCTGAAAAATGGCTGGGGAGACTGGAGCCCAAAAAGACAGGCATGGAACGCCTGGGGACTGATGCAGGTGGATGTTAATCCAGAGGGTGGTGGACATACACCCAGAGGTGCCTGGGACAGTGAGGAACATATTCTCCAGGGCACAGAAATATTAATAAGTTTTATTGGAAAAATCAGGAAGAAATTTCCCAAGTGGTCAAAAGAGCACCAACTAAAAGGAGCCATCGCAGCCTACAATCAGGGGGATGGAAAAGTTCATTCTTTTGAAAATGTAGATGAAAATACAACAGGGAAGGACTACTCCAATGATGTTATTAGCAGAGCTTTGTGGTACCAAAGAAATGGTTATAAAAACTGA

>Perch_LygF1

ATGGGTTATGGAGACATCATGAGGGTTCAAACTTCTGGAGCTTCATGGCAAACTGCTAAACAGGACAGGCTGGGATACTCCGGTGAGCGTGCATCACACACCATGGCAAAGACTGACAGGGACAGAATGAATATGTACAAGTCAGCAATCGTCAGAGTGGGAAACGCACATGAAATCGATCCAGCTCTCATCGCTGGCATCATCTCCAGAGAGTCCAGGGCTGGAAATGCACTGGTCGGAGGCTGGGGAGACCACGGCAAAGCCTGGGGACTGATGCAGGTTGATGTTACTCCAGGTGGAGGTGGACACACCCCTAAGGGTGACTGGAACAGCGAGGAACACCTCCACCAAGGAACTGAGATCCTGGTTTATTTTATCAACCGGATCCGCCAGAAATTTCCCGGCTGGAGCAGAGAGCAGCAGCTGAAAGGAGGGATAGCGGCCTACAATATGGGGGATAGAAACGTGCACTCCTATGACAACGTGGATGAAAACACAACAGGGAAAGACTACTCCAATGATGTTGTTGCCAGAGCTCAGTGGTACAAAAGCAACGGCTTTTAA

>Platyfish_LygF1

ATGAGTTATGGAGACATCAACAGAGTTGAAGCTTCAGGTGCCTCTGAGAAGACCTCTCAGCAGGACAGACTGGGTTATTTTGGCGTGAGGGCATCAGAAACCATGGCCCAAACGGATTCTGGGAGAATGAATAAGTACAAATCAAAAATCACCAGAGTGGGAAGTCAGTCTGGTATCGATCCTGCCCTTATTGCTGCCATCATCTCCAGAGAGTCCAGAGCTGGAAACGTCCTGCATGACGGTTGGGGGGATCACGGTAACGCCTGGGGACTCATGCAGGTGGACATTAGACATCACCGTAAGGAAGGAGACTGGGACAGCGAGGAGCATCTCCGCCAAGCTACAGGGATCCTTGTTCATTTCATCAAAAGGATTCAGAACAAGTTCCCGAACTGGAGCAAAGAGCAGCAGCTGAAAGGGGGAATAGCGGCCTACAACACGGGGGATGGTAATGTCCATTCGTATGAAAATGTGGATGAAAAAACGACTGGCAAAGATTACTCCAATGATGTTGTTGCCAGAGCCAAGTGGTACAAAAGAAACGGTTTCTAA

>Seabream_LygF1

ATGAGTTATGGAAACATCATGGATGTTGAAACTACTGGTGCCTCAGCAGAAACAGCTAAGTCGGACGGGCTGGGATACACAGGAGTGAAAGCATCACACAAGATGGCAGAGACTGACGCCGACAGAATGGAGAAGTACAAATCTAAAATCAACAGTGTTGGAGTTAAATATGGAATTGATCCAGCTATCATCGCTGCCATCATCTCCAGAGAGTCCAGGGCTGGAAATCAACTAAAGGATGGCTGGGGAGATGGGGGTAACGCCTGGGGACTGATGCAGGTTGACGTGAATCCACAAGGAGGTGGACACACTAAACGGGGCGCATGGGACAGTGAGGAACACCTCTGCCAAGGCGCGGAGATCTTGGTCGATTTTATCAAACAGGTCACCAAAAAATTACCTAGCTGGACCAAGGAGCAGCAGCTGAAAGGAGGGATAGCAGCCTACAATTTTGGGGTTAAAAATGTCCAGACCGTTGCAGGTGTTGATGTTGGCACAAACCATGGAGACTACTCCAATGATGTTGTTGCCAGAGCTCAGTGGTACAAAACCAATAAGGGCTATTAA

>Medaka_LygF1

ATGTTTGCAGACATCAGGAATGTTTCAACCACAGGAGCCTCGGCTCAAACAGCCCGACAGGACAAGCTGACGGTGTCAGGTGTGAGTGCATCACACGCCATGGCGCAGACTGACGCCGGCAGAATGATGAGATATAAGGACAAGATCACAGCGGTGGGACACAGGCTTGGGGTTGATCCAGCTCTGATTGCCGGCATCATCTCCAGGGAGTCCAGGGCCGGAAACGTCCTGCAGAATGGCTGGGGGGACCATGGAAACGGCTGGGGACTGATGCAGGTTGATGTCAATCCTCGTGGAGGTGGACACAAGGCTGAGGGAGCGTGGGACAGCGAGGAGCATCTCTCCCAGGCCACCGGGATCCTCACTCATTTCATTCATCGGATGGAGAAGAAATTCCCAAACTGCTCCAAAGAGCAGCAGCTGAAAGGAGGCATCGCCGCCTACAACACTGGAGACGGACGAGTGCACTCCTGCGACGACGTGGATGCCCACACAACTGGCAAAGATTACGCCAATGATGTTGTCGCCAGAGCTCAGTGGTACAAGAACCACGGCTACTAA

>Medaka_LygF2a

NAATACAGAAACCTGGAAGAAATGAGGACAACCGGAGTCTCTGCTGTTACCGCCACGGCGAACAACTTAACCCGTGAAGGAGATCTAGTTCTCGGGGTGGAAGGATCCGAAATATTGGCCAAAAAAGACCTCAAGCCGATGTCGAAATACCGGAACCAAATCATGAACGTTGGACGCAAGCTCCGCCTCCACCCGGCTCTGATCGCCGCCATGATCTCCAAACAGTCGAACGCTGGACAGCAGCTCAAGCCCGATGGCCACGGAATGCACGACGCCAACTCTTACGGTCTCATGCAGATCAACAGGAAGTTCCACGCCGTGAAGGGCGAGCCGTTCAGCGAGGACCACATCGACGAGGGCTCCACCTACCTGATCCACCTCATCAAGACCGTGACCAACTGGAGGCCGGATTGGAGCAGAGAGCAGCACCTGAAAGGAGCGCTGGTCTGCTACATGGTGGGCCTGGAGAAGGAGAAGCTGAACTACGACGGAGAGCTGGACCAACAGACGCCCACGCGGGACTTCGCCAACGACGTCATCGCCCGGGCGCAGTTCTACGCCGAGAACGGTTTC

>Takifugu_LygF1a

ATGCCTTACGGAAAGATAGAGGATATAAAAACTAGTGGGGCCTCAGATGTGACGGCTGCACAGGATGGATTAAAGGAAGGAGGCTGGAAGAGCTCACACAGAATGGCAGAGATTGACTCAAATAGAATGGAAAATTACAGGACCATTATCAACGAAGCCGGACGGCAGTGTGACGTAGATCCAGCTGTCATTGCTGGAATCATCTCCAGAGAGTCCAGGGCTGGAAATCAGCTGATTAATGGCTGGGGAGATCACGGCAAAGCCTTTGGACTGATGCAGATTGATGTGACTCCACCTCCAAATGGAGGCGGTCACACACCAGTTGGAACGTGGGACAGTTTGGAACACTTGATCCAAGCCACCGAAATCCTGGTTGAGTTTATCGAAAGGATCAAGACTAAATTTCCTCGTTGGAATGCAGACCAACACCTGAAAGGAGCTTTAGCTGCCTACAATAAGGGTGAGAAAAATGTCGAGTCTTATGCAAGTGTCGATGCCAAAACAACAGGGAAGGACTACTCCAATGATGTCGTTGCCAGAGCTCAGTGGTACAAAAGCAACATGGGGTTTTAA

>Oriental_sole_LygF1

ATGACTTATGGCGGCATCGTGGTTATTACAGACTCTGGAGCGTCGTGGCATACTGCTGAGCATTACAAGTTGACATGCCAAGGTGTGGATGCATGTCGCACCATTGCGAAGACTCACAGTGAAAGAATCAGGAACTACAAGACGGAAATTGAAGGTGTGGGACGTATATATGGCATAGAGTCGACTCTCACTGCTGCCATCAGCTCCCGAGAGTCCAGGGCCCGCTATGTAGTACATGATGGCTGGGAGGACTGGAATCCAAGCAGACAGGCCTATAATGCGTGGGGACTGATGCAGGTTGATGTCAATCCAAATGGAGGTGGACACACTGCTCGAGGTGAATGGGACAGCGTGGAACACCTCTGCCAAGCCACTGAGATCTTGGTTTATTTCATTGACCAAATCCGTAAGAAAATTTCAGACTTGAGCAAAGAGCAACAGCTGAAAGGAGCGATTGCAGCCTACAATGCCGGGGATAGAAAAGTCCATTCCTATGCTAAAGTGGAAGAAAACACAACTGGGATAGACTACTCGGATGATGGTGTTGGCAGAGCTGAGTGGTACGAACACAATCGCTTCAACGGCTGA

>Cod_LygF1b

ATGGGAATTAAAGAGTTCAAGCCAATCTCCAAGATATTCTGGGGGATAATTCTACTTACTTCACTCAATTTCTGTCCAGGAATTCATTCTGTAGGGTACGGAGACATCACGCAGGTAGAAACGTCCGGAGCGTCCAGCAAAACTTCGCGACAGGATAAACTGGAATACGACGGTGTAAGGGCATCGCATACAATGGCTCAAACCGATGCTGGGAGAATGGAAAAATACAAGTCCTTCATTAACAATGTGGCGAAAAAGCATGTTGTTGACCCAGCTGTCATCGCTGCCATCATCTCCAGAGAGTCCCGTGCCGGGAACGTCATCTTCAACACTACCCCCCCTGGTTGGGGGGACAATTATAATGGCTTTGGACTGATGCAGGTTGATAAGAGATACCACGAACCGAGAGGAGCTTGGAACAGTGAGGAGCATATTGACCAAGCCACTGGGATCCTGGTTAATTTCATTCAACTGATCCAGAAGAAGTTCCCCAGCTGGAGCACAGAACAGCAGCTGAAGGGAGGGATCGCAGCCTACAACACTGGAGACGGTAGAGTCGAATCTTATGAGAGTGTGGACTCACGCACCACAGGAAAAGACTACTCCAATGATGTGGTTGCCAGAGCCCAGTGGTACAAAAAAAACGGGTTTTAG

>Cod_LygF1d

ATGCGACAAGGGTACGGAGACATCATGAGGGTAGAAACATCTGGAGCGTCCAACAAGACCGCCGGAGCTGACAGGCTGACAGGTGGTGTACAGGCATCGCGAGAAATGGCCAACCATGATTTGGCTTGTATGAGAACATATAAGACCATCATTGGGAACGTGGCGCGCAGACGTAATGTTGACCCAGCTCTCATCGCTGCCATCATCTCCAGAGCAACCCGGGGTGGTGCCGCCATCTCCGGCACCAATGGTTGGGGGGACAATGGTAATGGCTTTGGACTGATGCAGGTTGATAAGAATTGGCACCAACCAAGAGGAGCCTGGAACAGTGAGACGCACCTGGACCAAGCCACTGAGATCCTGGTTGATATGATTAGTGTTGTCCGGGGGAAGTTCCCCGGTTGGAGCCCGGAACAGCACCTGAAGGGAGCGATTGCGGCCTACAACATGGGAGAACGTGTCCTAGAATATGCCGATGTGGACCATCTTACCACAGGCAACGACTACTCCTCTGATGTCGTTGCCAGAGCCCAGTTTTACAAACAAAACGGGTTTTAG

>Takifugu_LygF1b

ATGCCTTACGGAAACATAATGAGCATAGAAACAAGCGGTGCCTCCGCTACGACGGCTGCGGGGGACAGACTTGGGCCAGGCATACAAGGCTCGCGCGAAATGGCAAGGATTGACTTGGAGAGAATGAAAAAGTACAAGAGCATTATCAGACAAGCTGGACAGAAGTGTGATGTAGATCCAGCTCTCATTGCTGGCATCATCTCCAGAGAGTCCAGGGCTGGAAATCAGCTGGTTAATGGCCGGGGAGATCACGGCAGGGCCTTTGGACTGATGCAGATTGATCCCCAAAATAGTGGAATTACACCAGTTGGAAGTTGGGACAGTGTGGAACACCTGATCCAGGCCACCAAAATCCTGCTCTCTTTCATCGACGTGATCAAGAACAAATTTCCCAGCTGGAATGCAAACCAGCATCTGAAAGGAGCTATAGCCGCCTACAACATGGGTGATCAAAATGTCCGCTCCTATGAAAACGTTGATGCCGCCACAACTGGGCGGGATTACTCCAACGATGTCGTTGCCAGAGCTCAGTGGTACAAGCGCAACATGAGCTTTTAA

>Salmon_Lyg1

ATGGGAGTTGGAGTTGGAATTGGTGGATTCATTACATTCTTGACTTTAGCCTCTTGTTTTGGAGACATTACAAAGGTTGACACCAGTGGGGCCTCAGAGATAACCGCTAGACAAGACAAGCTAACTCTCCAAGGGGTGGATGCCTCTCATAAACTGGCTGAGCATGACCTGGTGAGGATGAACAAGTACAAGGAGCTCATCACCAGGGTGGGGCAGAAGCATGGCCTGGACCCAGCTATCATCGCTGGCATCATCTCCAGAGAGTCCCGGGCCGGGTCAGCGCTGGACCATGGCTGGGGAGATCATGGGAAAGGCTTTGGGCTCATGCAGGTTGACAAGCGCTACCACAAGATAGTGGGGGCATGGGACAGTGAAAAGCATATCAGTCAAGGCACTGAGATTCTCATTGAGTTTATCCGAAGGATCCAGGCTAAATTCCCTGCGTGGCCCAAGGAGCACCAGCTGAAAGGGGGAATTTCAGCCTATAATGCTGGGGACAAAAATGTCCGCACCTATGAACGAATGGACGTGGGCACCACTGGGGGTGACTATTCCAATGATGTTGTTGCCAGATCTCAATGGTTCAAAAGCCAGGGTTATTGA

>Pike_Lyg

ATGGGCTCCATTGGAGACATTACTAAGATTGACACCAGTGGTGCCTCAGAGAGAACAGCTAGACAAGACAAGCTTGCTGTCCAAGGAGTGGATGCATCTCACAATCTGGCTAGAATTGACTCAATGAGAATGAAACAGTATAAGGACCTCATCACCAGGGTGGGGCTGAAGCATGGCCTGGACCCAGCTATCATTGCTGGCATCATCTCTAGAGAGTCCCGGGCGGGGGCTGCATTGGATCGTGGCTGGGGAGACAAGGGGAATGGCTTTGGGCTTATGCAGGTTGACAAGCGTTTCCACAAGATAGTAGGGGCGTGGGACAGTGAAGAACATGTCAGTCAAGGCACTGGGATTCTTATTGAGTTCATACATAGGATCCAGGCGAAATTCCCTTCATGGCCCAAGGAGCACCAACTGAAAGGGGGTATTTCAGCCTATAATGCTGGGGACAAAAATGTGCGCACCTATGAGCACATGGACGTGGGGACCACTGGAGACGATTACTCCAATGACGTCGTCGCCAGAGCCCAGTGGTTCAAAAGCCAGGGTTATTGA

>Zebrafish_Lyg1

ATGGCATGCATTTATGGAGACATCATGAAAGTAGGCACCACTGGTGCATCAAAGAAGACAGCAGAACAGGACAAACTATCTGTAACGGGTGTTGAAGCCTCTAAAAAGCTGGCAGAGCATGATCTGGCCCGGATGGAGAAATACAAAACTAAAATCATTAATGTTGGTAGGGCAAAGCAGATGGACCCGGCTGTAATTGCTGCTATCATATCCAGAGAGTCCAGAGCTGGAGCCATTTTGAAGGATGGATGGGGTGACCACGGCAATGGTTTTGGTCTCATGCAGGTCGACAAGCGCTATCACACTCCAGTAGGTGCATGGGACAGCGAACAGCATCTCACACAAGCTACTGAGATACTGATTGGTTTTATAAAAGAGATCAAAGAAAAGTTTGCCAAGTGGAGCCAAGAACAATGCTTTAAAGGTGGAATATCAGCGTATAATGCAGGTGTGAAGAATGTTCAGACATATGAGCATGTGGATGATCACACCACAGGCCATGACTATGGCAATGATGTTGTTGCCCGAGCCCAGTGGTTCAAAAGCAAAGGATACTGA

>Smelt_Lyg

ATGGCTAGTCTTTTTGGCGACATCATGAAAGTGGAGACCAGTGGCGCTTCAGAGAAAACTGCCAAACAAGACAAGCTGACTGTCAAAGGGGTGGAGGCATCCCACAAGATGGCGGAGCATGATATGAAGAGAATGACCCAGTACAAGAGCATGGTGCAAACGGTGGGCCATTCAAAAGGCCTGGATCCGGCCATCATAGCTGGAATCATGTCCAGGGAGTCCCGAGCCGGTGCTGCTCTGGTCAACGGCTGGGGAGACCATGGCAACGGCTTTGGTCTTATGCAAGTCGACAAGCGTTTCCATAAGCTAGTTGGCGAGTGGGACAGCAAGGAGCATGTGAACCAGGCAACAGGGATTCTTGTTGATTTTATCAACACAATCCAGAAGAAGTTCCCTAAATGGCCAAAGGAACACCAGCTGAAGGGGGGAATATCGGCCTACAATGCAGGGGCCAAAAATGTCCAAACCTATGAGCGTATGGATGTGGGCACCACCGGGGATGACTATTCCAATGACGTGGTGGCCCGGTCACAGTGGTTCAAAACTCAAGGCTTCTAA

>Trout_Lyg1

ATGGGGGTTGGAGTTGGAGTTGGAATTGGTGGACTCATTACATTCTTGACTTTAGCCTCTTGTTTTGGAGACATTACAAAGGTTGACACCAGTGGGGCCTCAGAGATAACCGCTAGACAAGACAAGCTAACTGTCCAAGGGGTGGATGCCTCTCATAAACTGGCTGAGCATGACCTGGTGAGGATGAACAAGTACAAGAAGCTCATCACCAGGGTGGGGCAGAAGCATGGCCTGGACCCAGCTATCATCGCTGGCATCATCTCCAGAGAGTCCCGGGCTGGGGCAGTGCTGGACCATGGCTGGGGAGATCATGGAAATGGCTTTGGGCTCATGCAGGTTGACAAGCGCTACCACAAGATAGTGGGGACATGGGACAGTGAGGAGCATATCAGTCAAGGCAGTGAGATTCTCAAAGAGTTTATCCGAAGGATCCAGGCTAAATTCCCTGCTTGGCCCAAGGAGCACCAGCTGAAAGGGGGAATTTCAGCCTACAATGCTGGGGACAAAAATGTCCGCACCTATGAGCGAATGGACGTGGGCACCACTGGGGGTGACTATTCCAATGATGTTGTTGCCAGATCTCAATGGTTCAAAAGACAGGGTTATTGA

>Zebrafish_Lyg2

ATGGGCATTCCGGTGATACTTACCATGTATTTTCTAGCATGCATTTATGGAGATATCATGAAAATAGACACCACTGGGGCATCAGAGGTGACAGCAAAACAGGACAAGTTAACTGTAAAGGGAGTTGAAGCCTCTAAAAAACTGGCTGAGCATGATCTGGCCCGAATGGAACAATACAAGTCCAAAATCCTCAAAGTTGCCCGAGCAAAGCAGATGGACCCGGCTGTGATTGCTGCCATCATATCCAGAGAGTCCAGGGCTGGAGCGGCACTGAAGGATGGATGGGGTGACCACGGCAATGGCTTTGGTCTCATGCAGGTTGACAAACGCTACCACAAACTGGTAGGTGCGTGGGACAGCGAGGAACATCTCACACAAGGAACTGAGATACTCATTGGTTATATTAAAGATATTAAAGCAAAGTTTCCCACATGGACCAAGGAGCAATGCTTTAAAGGTGGAATATCAGCGTATAATGCAGGTGTGAAGAACGTGCAAACATATGAGCGCATGGATGTGGGCACCACAGGCGGTGATTACGCTAATGATGTTGTTGCCCGAGCCCAGTGGTTCAAAAGTAAAGGTTACTGA

>Zebrafish_Lyg3

ATGGGTAACACAGAATCTGGCATTGACATCATGGATATAGACACCAAGGGTGCATCACCGGTGACTGCCAGTCAAGACAAATTAACCGTAAAGGGGGTTGAAGCCTCTAAAAAACTGGCTGCAGCTGATTTCGTCCGGATGGAGAAATACAAGTGTAAAATCTTCAAAGTTGCCAAAGAAAAGGACGTGGACCCAGCTCTGATTGCTGCCATCATATCCAGAGAGTCCAGAGCCGGACACACTTTGTTGAAAGGATGGGGCGACCACGGCAATGGTTTTGGCCTCATGCAGGTTGACAAGCGCTATCACACTCCAGTAGGTGCATGGGACAGTGAACAGCATCTCACACAGGCTACGGAGATACTCATTGACTTCATTAGGAAAATTAAAGAAAAGTTTCCTAGATGGTCCCTTGAGCAATGTGTTAAAGGTGGAATAGCAGCCTACAATGCTGGTGTGAATAAAGTGAAGTCATATGAGGACGTGGATGAGGGCACCACAGGCCGTGATTATGCCAATGATGTTGTTGCCCGAGCCCAGTGGTTCAAAACTGTAAAAGACGGTTACTTGTTATAA

>Channel_Catfish_Lyg

ATGGCTGGCATTTTTGGAGACGTCACGAAGATCGACACGACTGGGGCATCGGAAATAACAGCCAAGCAGGATAAACTCACTGTTAAAGGGGTGGAAGCCTCAAACAAACTGGCTGAGCATGATCTGAAGAAGATGGAGCAGTACAAGAGCATCATCACCAAAGTTGGCAGAGCTAAGAAGATCGACCCAGCTGTGATCGCAGGCATTATATCCAGAGAGTCGAGGGCTGGAGCAGCGCTTGTTGATGGCTGGGGAGACCACGGGAACGGCTTCGGACTCATGCAGGTTGACAAGCGCCACCACACTCCAAAAGGAGCCTGGAACAGTGAGGAGCACGTCACCCAAGCCACCGAAATTCTAATAGAATCCATTCAAGCAATTCAAAAGAAATTCCCCAGCTGGTCCAAGGAGCATCAGTTAAAAGGAGGAATCTCAGCCTACAACGCTGGCCCAGGAAATGTCCGCACGTATGAGAAAATGGACAGAGGCACCACAGGAGATGATTATGCCAATGATGTGGTGGCCCGGTCTCAGTGGTTTAAACGCAATGGCTACTAA

>Grass_carp_Lyg

ATGGCGTGCATTTATGGAGACGTCATGAAAATAGACACCACGGGGGCATCAGATTCGACAGCAAAACAGGACAACTTAACCGTAAAGGGTGTTGAAGCCTCCAGAAAACTAGCTGAGCATGATCTGGCCCGGATGGAGAAATACAAGAGTATAATCATTAAAGTTGGAAGAGCAAAGCAGATGGACCCGGCTGTGATTGCTGCCATCATATCCAGAGAGTCCAGAGCCGGAGCCGCTCTGATAGATGGGTGGGGCGACCATGGCTATGCCTTTGGCATCATGCAGATTGACAAACGCTATCACACTCCAGTAGGTGCATGCGACAGTGAGCAGCATATCACACAAGGAACTGAGATACTCATTGGCTTCATTAAAGAAATTAAAGCAAAGTTTCCCCAGTGGACTCAAGAGCAATGCTTTAAAGGGGGGATATCAGCCTACAACGCAGGTCCGGGGAACGTCCGTACATACGAGCGCATGGACGTGGGAACTGCAGCCGGCGATTACTCCAATGATGTTGTGGCCCGAGCCCAGTGGTACAAAAGCAAGGGTTACTGA

>Common_carp_Lyg

ATGGCATACATTTATGGAGACACCATGAAAATAGACACCACCGGTGCTTCAGAGGCAACAGCAAAACAAGACAAATTAACCATTAAGGGGGTTGAAGCCCCTAAAAAACTGGCTGAGCATGATCTGGCCCGGGGGGAGAAATACAAGAACATGATCACTAAAGTTGGCAAAGCAAAAAAGATGGATCCGGCTGTGATTGCTGCTATGATATCCAGAGAGTCCAGAGCTGGAGCCGTCCTGAAGAATGGATGGGAACCCGCAGGCAATGGTTTTGGCCTTATGCAGGTTGACAAACGCTCCCACACTCCGGTTGGTGCATGGGACAGTGAGCAGCATGTCACACAAGCTACAGAGATACTCATTGGCTTCATTAAAGAAATTAAAGTAAATTTTCCCAAGTGGACACAAGAGCAATGCTTTAAAGGGGGAATAGCAGCCTATAACAAAGGTGTGAGTAGAGTCACTTCGTATGAGAATATCGATGTCAAAACCACAGGACTAGATTACTCCAGTGATGTTGTTGCCAGAGCCCAGTGGTTCAGAAGCAAGGGTTACTGA

>Catfish_Lyg

ATGGCTGGCATTTTTGGAGACGTCACAAAGATCGACACGACTGGGGCATCGGAAAAAACAGCCAAGCAGGATAAACTCACTGTTAAAGGGGTGGAAGCCTCAAATAAACTGGCTGAGCATGATCCGAAGAAGATGGAGCAGTACAAGAGCATCATCACCAAAGTTGGCAGAGCTAAGCAGATCGACCCAGCTGTGATCGCAGGCATTATATCCAGAGAGTCAAGAGCTGGAGCAATTCTTGTGAATGGCTGGGGAGACCACGGCAACGGTTTCGGACTCATGCAGGTTGACAAGCGCCACCACACTCCAAAAGGAGCCTGGAACAGTGAGGAGCACGTCACCCAAGGCACCGAAATTCTAATAGAATCCATTAAAGCAATTCAAAAGAAATTCCCCAGCTGGTCCAAGGAGCATCAGTTAAAAGGAGGAATCTCAGCCTACAACGCTGGCCCAGGAAACGTCCGCACGTATGAGAACATGGACAGAGGCACCACAGGAGATGATTATGCCAATGATGTGGTGGCCCGGTCTAATTGGTTTAAACACAATGGCTACTGA

>Cavefish_Lyg1

ATGGCTTGCATATTTGGAGACGTAATGAAGATCGACACTACTGGAGCGTCTGAGAAAACAGCACGCCAGGACAAACTAACAGTTAAGGGGGTGGAAGCCTCGAATAAGCTGGCCGAGCACGACCTGAAGAGAATGCAGCAGTACAAGTCCATCATCACCAGAGTTGGGGCAGCGAAGCAGATGGATCCAGAAGTGATTGCTGCCATCATATCCAGAGAGTCGAGGGCCGGAGCTGCTCTTGTGGACGGCTGGGGAGATCACGGCAATGCTTTCGGACTCATGCAGATCGACAAGCGCTACCACACTCCGAAAGGGGCCTGGAACAGTGAAGAGCACGTCACACAGGGCACTGAGATTCTGATCGATCTAATTCAGAAAATTCAGAAAAAGTTCCCCAGCTGGCCCTTAGAGCACCAATTTAAAGGAGGAATAGCAGCCTACAACGCTGGCGTCGGGAACGTTCGCACATACGAGCGGATGGACATCGGGACCACGGATGACGATTACTCTAACGATGTGGTCGCCAGGGCCCAGTGGTTTAAACGCAACGGCTACTGA

>Tetraodon_LygF2

NAGTATGGCCACACAACCTGCCTGGAAACGAGCGGAGCTTCTGAAGACACCGCCATCGCTAATGGACTGAGAGTCCGAGGAACCGAAGCCTCCAACATCCTGGCAGCCAAAGACCTGAAGAAGATGAAGAAGTTCAAAGATGACATCACCAGTGTTGGACAAAGACTCGGCGTGGAGCCGGCCCTGATCGCCGCCATCATCTCCCGCCAGTCGCAGGCTGGAACCAACCTGAGCACCAGCGGCTACGGCGTCTCTGACCCCAACTGCTTTGGCCTCATGCAGATCAACAAGCATTACCACGCTGTCAAGGGGAACGCGTACAGCAGCGAGCACATCGACCAGGGGGTCACCTTCCTGATCCAGCTCATTAAGACCATGAGGCGCACCAGACCCGACTGGAGCAAAGAGCAGCAGCTGAAAGGTGCGCTGGCCTGTTACGTGGCAGGAGAGGAGCGAGTCCTGGCCTTGTCGTACGAGGACTTGGACAGCGTGACGCCCAGCAAGGACTTCAGCAGCGACGTGGTCGCCAGAGCTCACTGGTTCGCCCAGAACGGCTTCTGA

>Tetraodon_LygF1

GTAGCTTACGGAAACATAATGAAAATAAGCACAACCGGTGCGTCTGCTGTGACGGCTAGACAGGACAGACTTGAACCAGTAAAAGGCGTGGCTGCGTCAGAAAAAATGGCACAGATTGATGTAGAGCGGATGAGAAGATACACCGGTATAATCAGCAGAGCAGCCCAGCAGTGTGACGTGGATCCAGCTCTCATCGCGGGGATCATCTCCAGAGAGTCCAGGGCTGGAAACCAGCTGGACAACGGCTGGGGAGATAACGGCAAGGCCTGGGGGCTGATGCAGGTGGATGTTACCCCAAACGGGGGTGGACACACACCAGTTGGAAACTGGGACAGTTTGGAACACCTGGTACAGGCCACCAATATCCTTGTCTACTTTATCGGAAGGATCAGGGACAAATTTCCTACATGGGATGCAAACCAGCACCTGAAAGGAGCTATAGCTGCCTACAATATGGGTGATGGGAATGTCGAGCCCGGTAAAGACGTCGACGCCAACACGACTGGGGGGGACTACTCTAATGACGTGGTTGCCAGAGCTAAGTGGTATAAGCGAAACTTGAGTTTTTAA

>Stickleback_LygF1

NGTTATGGAGACATCATGAAGGTTCCAACTACTGGAGCGTCGCTGCAAACTGCTAAGCAGGACAAGCTGACCTACGCAGGTGAGAAGGCGTCACATACCCTGGCACAGACGGATAAGAACAGAATGGAAAAGTACAGGTCTAAAATCAACACAGTGGGAGCAAAGTATGGAATCGATCCAGCTCTCATTGCTGCCATCATCTCCAGAGAGACCAGGGCTGGAAATTGCTTACAAGGAGGCTGGGGAGACGGCGGCAATGCCTGGGGACTGATGCAGGTTGTTGACGTCAATCCAAATGGAGGTGGACACACTAAACGCGGTGACTGGGACAGTGAGGAACATCTCTCCCAAGCCGCAGAGATCTTGGTTCATTTTATCGGACGCATCCGCAACAAATTTCCCAGCTGGAGCGCAGAGCAGCAGCTCAAAGGTGGGATAGCTGCCTACAACATGGGAGATGGAAACGTCCATTCCTATGATCAAGTTGATGCCAACACGACAGGAAGAGACTACTCCAATGACGTTGTGGCCAGAGCGCAGTGGTACAAAAACAATGCAGGCTTTTGA

>Lamprey_Lyg

NGAGGGAGTGGAACCGGTTGCTATGGTGACCTGATGAACATTGACACAACGGGAGCTTCGATTAAGACTGCCAAACAAGACAAGCTGAGCGAGAGGGGAGTGGCCGCATCAAAGAAGCTGGTGAACGCCGACCTGATGCGCCTCAAGAACTACAAGACCATCATCGTGGCCACCGCCAATGCAAAGTGCATGGACCCGGCTGTCATCGCGGCCATCATCTCTCGCGAGTCGCGTGCCGGCGCTCTGCTCGTTAAAGGCTTTGGCGATAACGGAAATGGCTTCGGACTCATGCAGGTGGACAAGCGCTTCCACAACATAGTGGGCACGTGGGACAGCGAAGACCACTTGAAGCAGGGCACGGGCATCCTCATCGACATGATCAGTGCCATCAAGAAGAAGTTCCCTAACTGGACCCAGGACCAGCAGATGAAAGGAGGCATCTCGGCATATAACGCTGGCACGCGGAACGTGGGGTCGTATGCCAACATGGACATCGGCACGACGGGCGACGACTACGCCAACGACGTGGTCGCCAGGGCCCAGATGCTCCGTGACAACGGCCACTTCTAG

>Florida_lancet_Lyg

ATGCTGTTCGTTGTGCTCTCGGCCTTTGTGGCAGTCGCTGCCGCATCAGGAAACTATGGAAACATCATGGCGGTGGACACTACCGGTGCCAGCGCACAGACCGCCAGCCAGGACGGCATCGGCTACGGAGGTACCAGTGCCTCCCAGCAGATGGCCAGGACAGACCTCAACCGCCTGAACACCTACAAATCGAAGATCTATAACGCCGCCAGCGCCAAGAACATGGACCCCGCCGTCATCGCCGCCATCATCAGCCGTGAGTCCCGCGCAGGCGCAGCACTGGCCTCGGACGGAACAGGGGATAATGGGAACGGATACGGGCTCATGCAGGTGGACATCCGTTACCACACCCCCCAGGGCGGTCCGTACACCACGACTCACATCAAGCAGGGAACCCAGATCCTCATCGATACCATCAACTGCGTCAAGCGCAACCACCCTGGCTGGAGCACCGAGATGGCCCTCAAAGGCGGAATCTCCGGGTACAACGCCGGGTGCGGTAACGTGCAGACCTACAACGGGATGGACATCGGTACTACCGGGGATGACTACGGGAATGACGTGGTCGCGCGCGCGCAGTGGCTGAAGAGGAACGGCTACAACTGA

>Japanese_lancet_Lyg

ATGATGTTTCTGGTGATTTTGGCCATTGTGGGAGTCGCCACTGCAGATGATTGGCAGTGCACAACCAGCGTTGCAGCTAACGGCCAGAACGGCCAGTGCGCACACGTCGACACCTGCCCGTACCACTACTTCGTCTCCAACAAGTGTCCCAGCTACGGAAATGACGTCAAGTGTTGCTACAACTGCCACCTGGGCGGATGCTCTATCTCCGGTGGCAGTTCAGGAGGCACTGGTGGAAGTGGAAGTGGAAGCTACGGAAACATCATGGAAGTGGACACAACTGGAGCTAGCTCACAGACGGCTAGCCAAGATAACATCTGGTACAGCGGTGTTAGCGCCTCCCACCAGCTGGCCAGTAACGACTTGGGCCGCCTGAACAACTACAAGTCGCAGATTTTTGACGCCGGAAACGCCAAGAACATGGACGCCGCCGTCATCGCCGCCATCATCAGCCGTGAGTCACGCGCAGGCGCAGCACTGGCCGCGGACGGAACAGGAGATAATGGAAACGGATTCGGGCTCATGCAGGTGGACTACCGTTACCACACTCCTGCAGGCGGTCCGTACAGTACCGAGCACATGATGCAGGGAACTCAGATCCTCATCGACACCATCAACTGTGTGAAGCGCAACCACCCTAACTGGAATGACAACATGACCCTCAAGGGCGGAATTTCCGGGTACAACGCTGGCTGTGGTAACGTGCAGACCTACGCCGGGATGGACGGAGGCACTACCGGTGAAGACTACGCCAATGATGTCGTCGCGCGCGCCCAGTGGCTGAAGGGACAAGGCTTCTAG

**Figure S7. Coding sequences for lysozyme *g* from diverse vertebrates**. Full-length and near full-length lysozyme *g* coding sequences used in this analysis are presented in fasta format with shortened species names. Sequences are from Tables S1 and S2. Ns are added to the beginning of some sequences to generate correct reading frames.
