# Supplementary material for: Evolution of the vertebrate goose-type lysozyme gene family
Source: BMC Evol Biol. 2014 Aug 29;14:188. doi: 10.1186/s12862-014-0188-x (PMC4243810; doi:10.1186/s12862-014-0188-x)
Supplement: Additional file 11: Figure S8. — Phylogeny of vertebrate lysozyme g genes. [file 12862_2014_188_MOESM11_ESM.pdf]

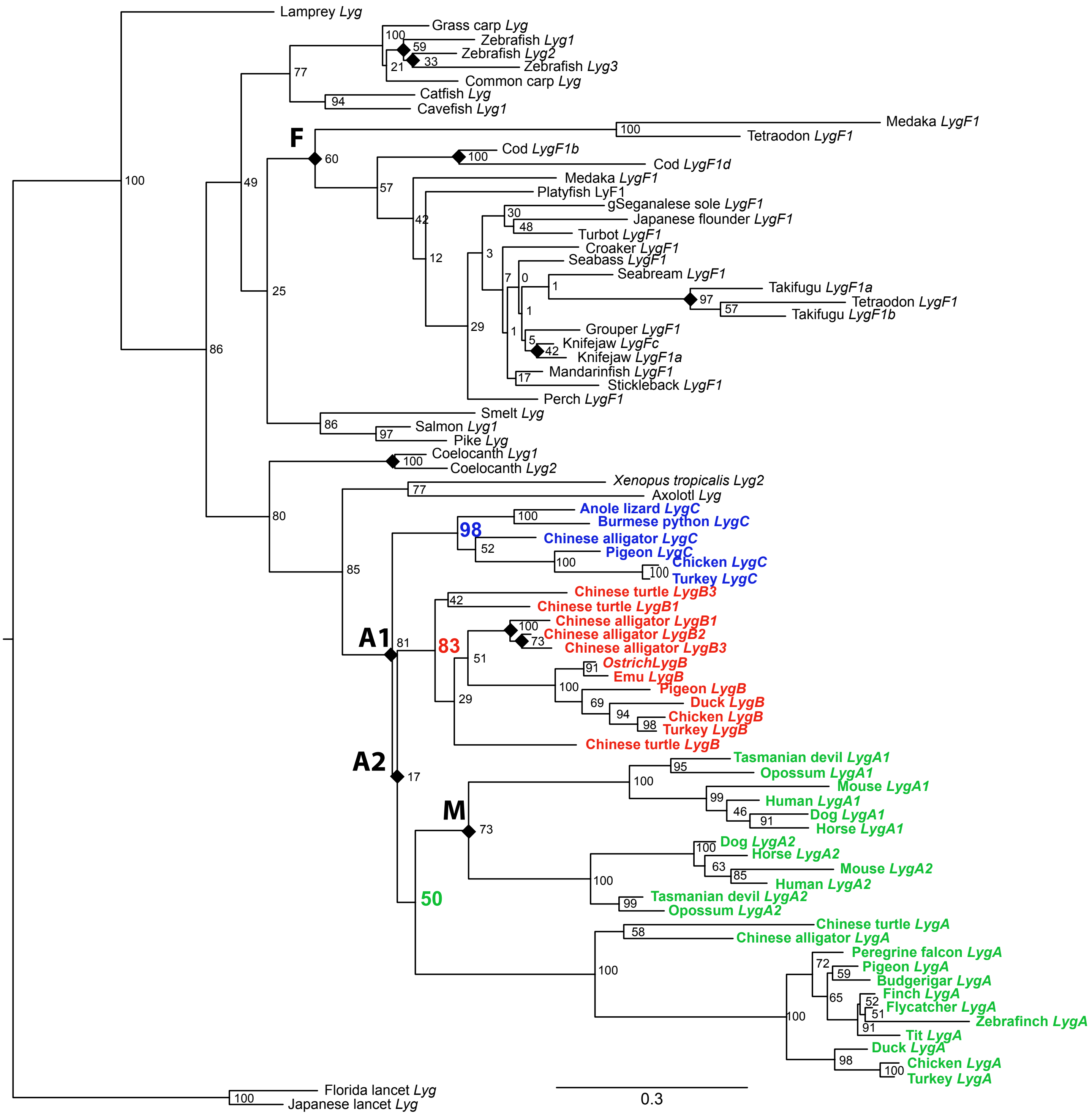

Figure S8. Phylogeny of vertebrate lysozyme *g* sequences. Phylogeny of lysozyme *g* sequences from diverse vertebrate species generated by Maximum likelihood. Phylogeny was rooted with sequences from lancets. Numbers at the nodes are the proportion of bootstraps supporting the nodes. Branch lengths are proportional to the amount of inferred change, with the scale bar at the bottom. Diamonds indicate gene duplication events. **A1** and **A2** refer to the duplications in the ancestor of mammals, birds and reptiles (amniotes), **M** to the duplication on the mammalian lineage, and **F** the duplication in an early teleost fish lineage. Orthologs in amniotes of the chicken *LygA* gene are labeled in green, chicken *LygB* in red, and chicken *LygC* in blue.
