# Supplementary material for: Evolution of the vertebrate goose-type lysozyme gene family
Source: BMC Evol Biol. 2014 Aug 29;14:188. doi: 10.1186/s12862-014-0188-x (PMC4243810; doi:10.1186/s12862-014-0188-x)
Supplement: Additional file 12: Figure S9. — Phylogeny of mammalian lysozyme g genes. [file 12862_2014_188_MOESM12_ESM.pdf]

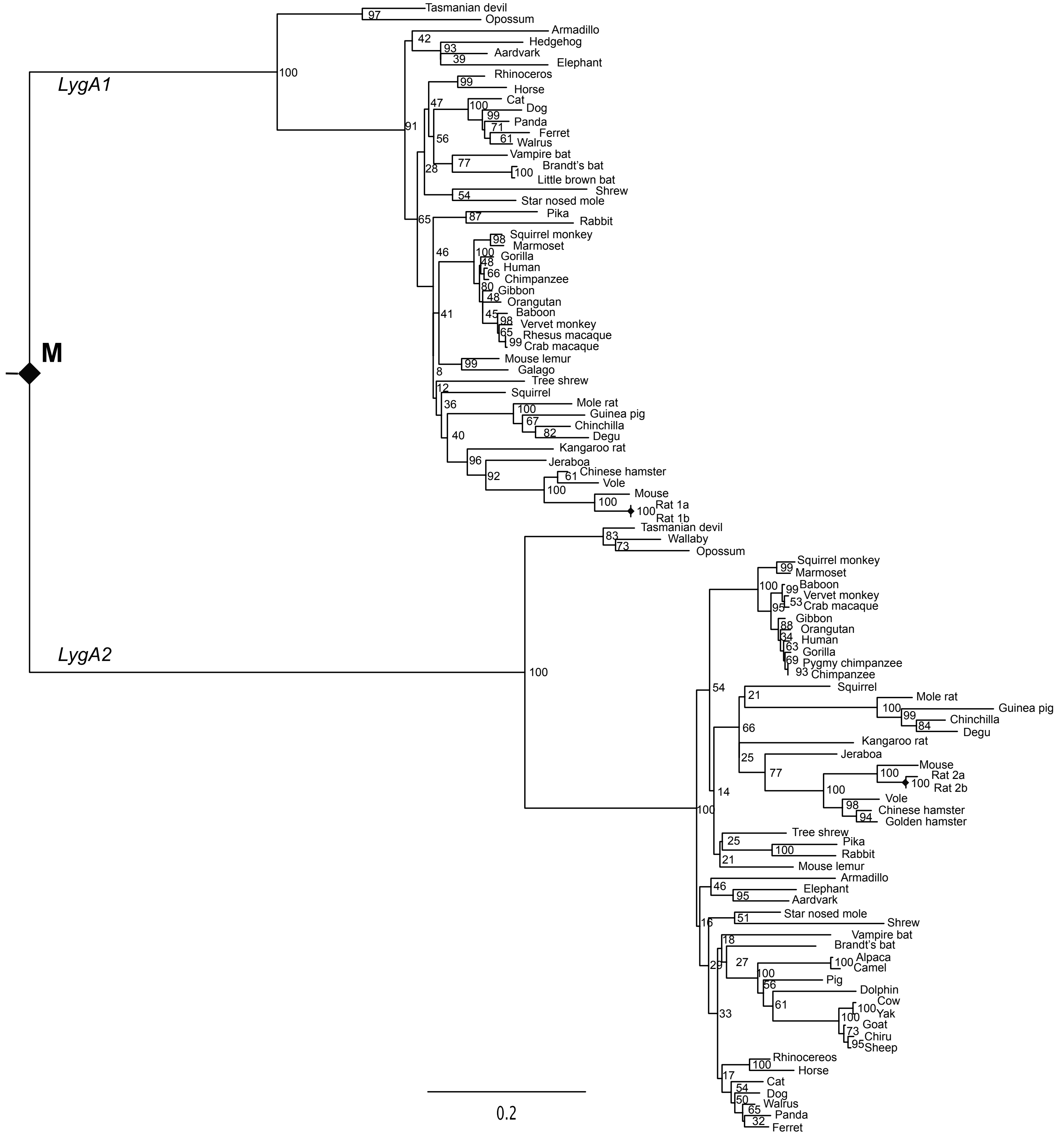

Figure S9. Phylogeny of mammalian lysozyme *g* sequences. Phylogeny of lysozyme *g* sequences from diverse mammalian species generated by Maximum likelihood. *LygA1* genes are in the upper portion of the tree, while *LygA2* benes are the lower part. Numbers at the nodes are the proportion of bootstraps supporting the nodes. Branch lengths are proportional to the amount of inferred change, with the scale bar at the bottom. Diamonds indicate gene duplication events. **B**, duplication on the early mammalian lineage.
