# Supplementary material for: Evolution of the vertebrate goose-type lysozyme gene family
Source: BMC Evol Biol. 2014 Aug 29;14:188. doi: 10.1186/s12862-014-0188-x (PMC4243810; doi:10.1186/s12862-014-0188-x)
Supplement: Additional file 13: Figure S10. — Phylogeny of fish lysozyme g genes. [file 12862_2014_188_MOESM13_ESM.pdf]

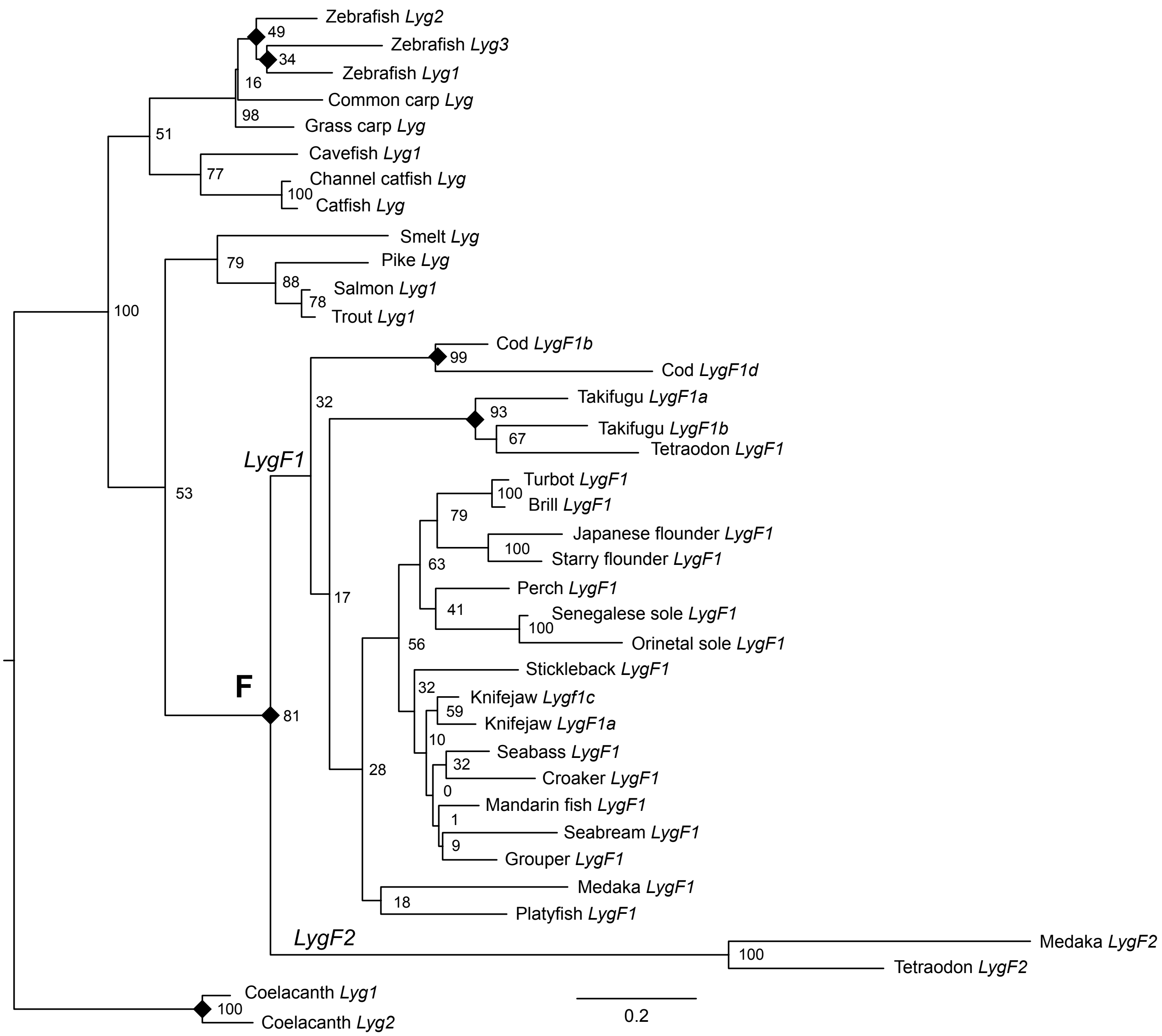

Figure S10. Phylogeny of fish lysozyme *g* sequences. Phylogeny of lysozyme *g* sequences from diverse fish species generated by Maximum likelihood. Phylogeny was rooted with the Coelacanth sequences. Numbers at the nodes are the proportion of bootstraps supporting the nodes. Branch lengths are proportional to the amount of inferred change, with the scale bar at the bottom. Diamonds indicate gene duplication events. **F**, duplication on an early teleost fish lineage leading to the *LygF1* and *LygF2* (labled on ancestral lineages) genes.
