# Supplementary material for: Evolution of the vertebrate goose-type lysozyme gene family
Source: BMC Evol Biol. 2014 Aug 29;14:188. doi: 10.1186/s12862-014-0188-x (PMC4243810; doi:10.1186/s12862-014-0188-x)
Supplement: Additional file 14: Figure S11. — Phylogeny of tetrapod lysozyme g genes. [file 12862_2014_188_MOESM14_ESM.pdf]

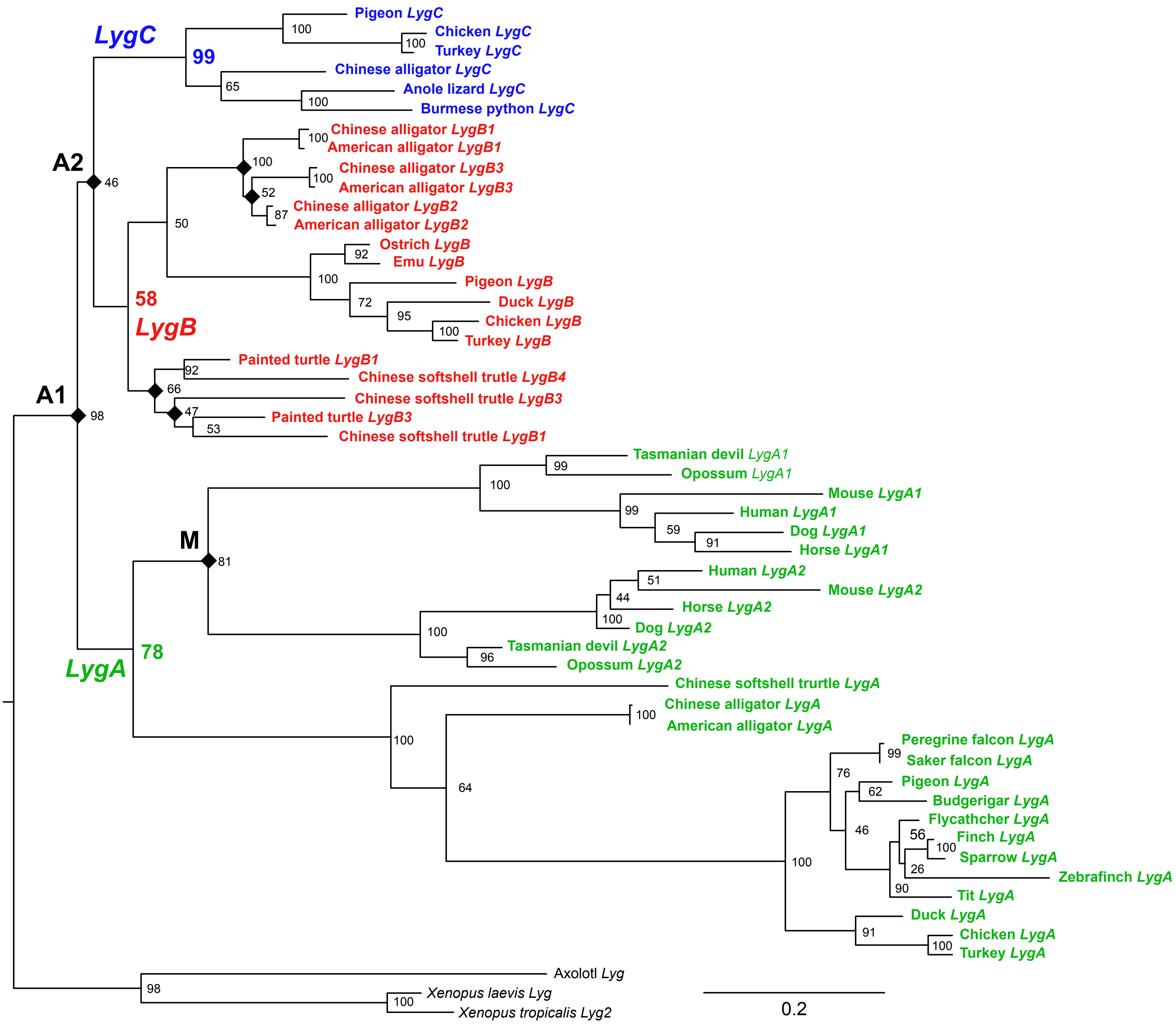

Figure S11. Phylogeny of tetrapod lysozyme *g* sequences. Phylogeny of lysozyme *g* sequences from diverse tetrapod species generated by Maximum likelihood. Phylogeny was rooted with the Amphibian sequences. Numbers at the nodes are the proportion of bootstraps supporting the nodes. Branch lengths are proportional to the amount of inferred change, with the scale bar at the bottom. Diamonds indicate gene duplication events. **A1** and **A2** are the duplication events in the ancestor of mammals, birds, and reptiles (amniotes), and **M** is the duplication on the early mammalian lineage. Orthologs of the chicken *LygA* gene are labeled in green, chicken *LygB* in red, and chicken *LygC* in blue.
